# Mechanistic Investigation of Ni-Catalyzed Reductive Cross-Coupling of Alkenyl and Benzyl Electrophiles

**DOI:** 10.1021/jacs.3c02649

**Published:** 2023-06-26

**Authors:** Raymond F. Turro, Julie L.H. Wahlman, Z. Jaron Tong, Xiahe Chen, Miao Yang, Emily P. Chen, Xin Hong, Ryan G. Hadt, K. N. Houk, Yun-Fang Yang, Sarah E. Reisman

**Affiliations:** † The Warren and Katharine Schlinger Laboratory for Chemistry and Chemical Engineering, Division of Chemistry and Chemical Engineering, California Institute of Technology, Pasadena, California 91125, United States; ‡ Arthur Amos Noyes Laboratory of Chemical Physics, Division of Chemistry and Chemical Engineering, California Institute of Technology, Pasadena, California 91125, United States; § College of Chemical Engineering, Zhejiang University of Technology, Hangzhou, Zhejiang 310014, China; ∥ Department of Chemistry, Zhejiang University, Hangzhou, Zhejiang 310027, China; ⊥ Department of Chemistry and Biochemistry, University of California, Los Angeles, California 90095, United States

## Abstract

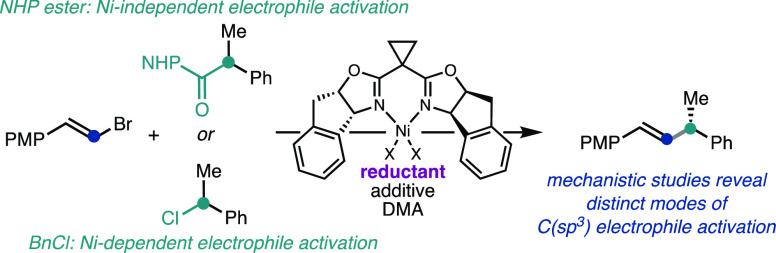

Mechanistic investigations of the Ni-catalyzed asymmetric reductive alkenylation of *N*-hydroxyphthalimide (NHP) esters and benzylic chlorides are reported. Investigations of the redox properties of the Ni-bis(oxazoline) catalyst, the reaction kinetics, and mode of electrophile activation show divergent mechanisms for these two related transformations. Notably, the mechanism of C(sp^3^) activation changes from a Ni-mediated process when benzyl chlorides and Mn^0^ are used to a reductant-mediated process that is gated by a Lewis acid when NHP esters and tetrakis(dimethylamino)ethylene is used. Kinetic experiments show that changing the identity of the Lewis acid can be used to tune the rate of NHP ester reduction. Spectroscopic studies support a Ni^II^–alkenyl oxidative addition complex as the catalyst resting state. DFT calculations suggest an enantiodetermining radical capture step and elucidate the origin of enantioinduction for this Ni-BOX catalyst.

## 1 Introduction

Ni-catalyzed reductive cross-couplings (RCCs) of organic electrophiles have emerged as useful reactions for C(sp2)–C(sp3) bond formation.^1^ These reactions provide direct access to cross-coupled products from readily available organic electrophiles, such as halides, precluding the need to pregenerate an organometallic coupling partner. The use of a metal powder (Mn^0^, Zn^0^) or an organic electron donor such as tetrakis(dimethylamino)ethylene (TDAE)^2^ provides reducing equivalents to render the system catalytic in Ni. Ni-catalyzed RCC reactions can also be driven electrochemically using either sacrificial anodes or paired electrolysis systems.^3^ A key challenge in the development of these reactions is achieving selectivity for the cross-coupled product over possible homo-coupling products; this requires a catalyst that oxidatively adds each electrophile in sequence or a catalyst system with mechanistically distinct modes of activating each coupling partner. Despite this challenge, several different Ni catalysis systems have been developed that afford high selectivity for cross-coupled products.^1,4,5^

**Figure 1:**
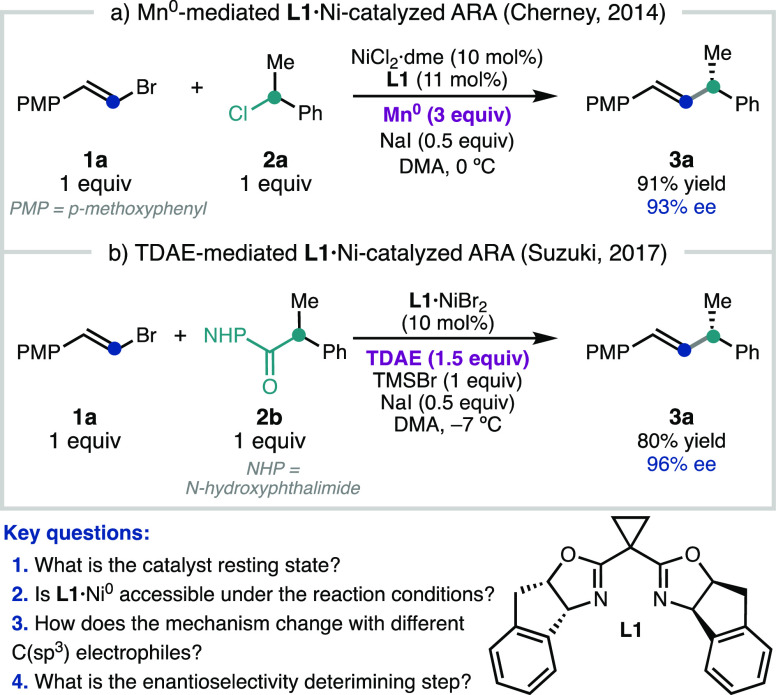
Ni-catalyzed asymmetric reductive cross-coupling between alkenyl and benzylic electrophiles.

Our lab has developed several Ni-catalyzed asymmetric reductive alkenylation (ARA) reactions (Figure 1), which leverage the intermediacy of C(sp^3^) radicals to enable stereoconvergent, enantioselective bond formation.^6−8^ In 2014, we reported an ARA between benzylic chlorides and alkenyl bromides using cyclopropyl-containing IndaBOX ligand **L1** and Mn^0^ as the terminal reductant (Figure 1a).^6^ We subsequently developed a related ARA that uses the same ligand (**L1**), but employs redox-active *N*-hydroxyphthalimide (NHP) esters as the C(sp^3^) coupling partner.^7^ In this case, TDAE was used as the reductant, and trimethylsilyl bromide (TMSBr) was identified as a key additive (Figure 1b). In addition to chiral ligand **L1** being optimal for both reactions, the use of DMA as solvent and NaI as an additive was shared between the two transformations. Given their similarities, we identified this pair of transformations as well suited for investigating the mechanism of Ni-catalyzed RCCs and how the mechanism might change depending on the C(sp^3^) coupling partner.

Since many RCCs use heterogeneous terminal reductants, the mechanisms of these reactions have been difficult to elucidate. Nonetheless, insightful studies of reductive arylation have been disclosed by the groups of Weix^9^ and Diao;^10^ these systems have primarily focused on reactions in which catalytically relevant Ni^II^(aryl)X complexes can be isolated and characterized. Diao and coworkers have also recently investigated bi(oxazoline)^10a,10b^ and pyridine-oxazoline^10c^ ligands in reductive arylation; however, mechanistic studies of reductive alkenylation and of Ni-catalysts supported by chiral bis(oxazolines) such as **L1** are lacking.^11^ Here, we report our mechanistic investigations of two **L1**·Ni-catalyzed ARA reactions. In this study, we sought to (1) determine the kinetic driving forces and resting state for the homogeneous reaction of alkenyl bromide **1a** with NHP ester **2b**; (2) investigate the redox properties of the **L1**·Ni^II^X_2_ precatalysts and determine whether **L1**·Ni^0^ is accessible using common reductants; (3) interrogate the mechanism of electrophile activation for both **2a** and **2b**; (4) use computational methods to understand the enantioselectivity determining step. These studies have revealed that chloride **2a** and NHP ester **2b** are activated through distinct mechanisms and provide insights that can guide the optimization of reaction conditions for Ni-catalyzed RCC reactions.

## 2 Results and Discussion

### 2.1 Reaction Kinetics of TDAE-Mediated RCC

**Figure 2:**
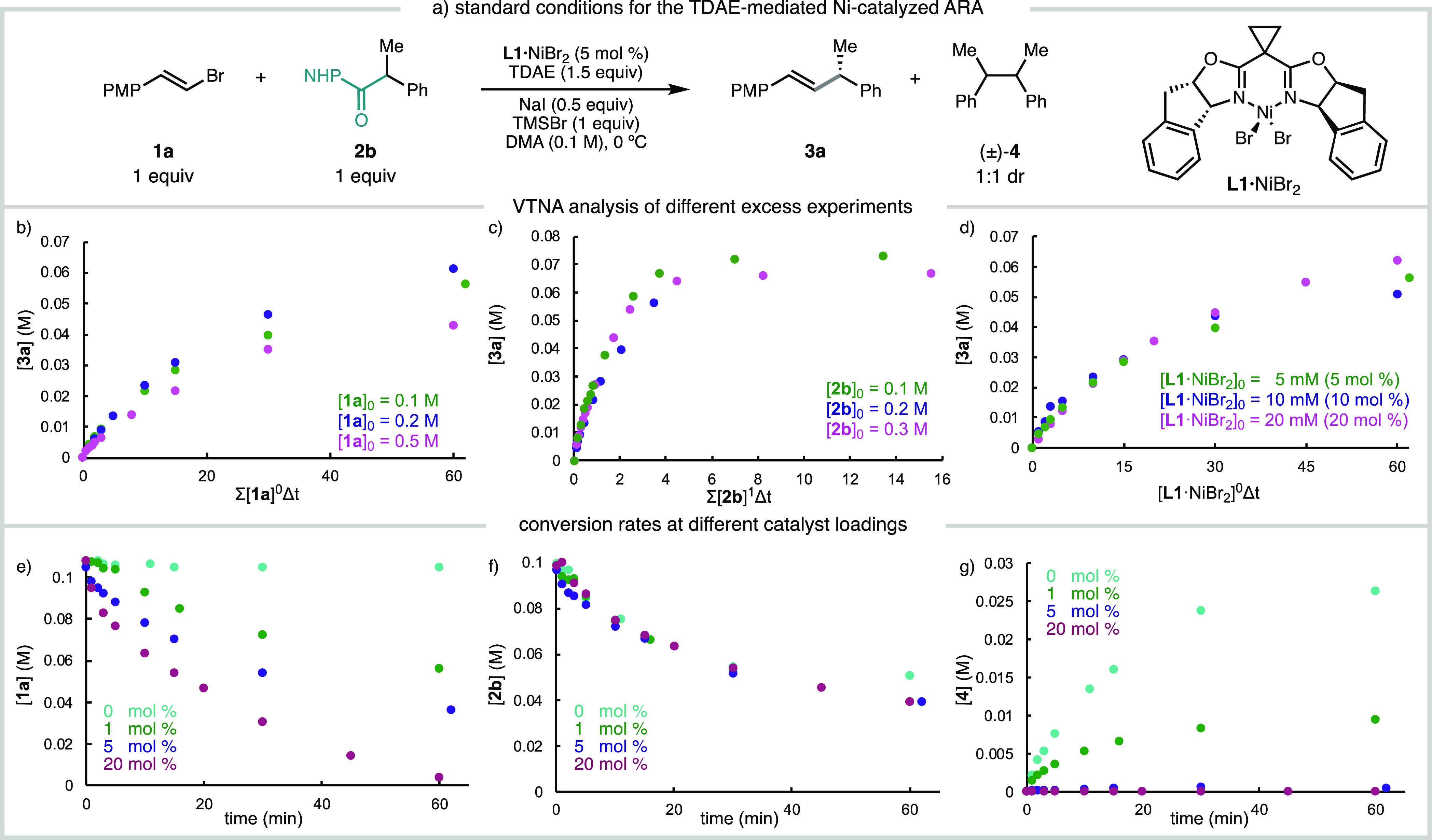
(a) Standard conditions for different excess experiments on TDAE-mediated ARA. Different excess VTNA experiments with varying initial concentrations of (b) alkenyl bromide **1a**, (c) NHP ester **2b**, and (d) catalyst **L1**·NiBr_2_. Impact of [**L1**·NiBr_2_] on the rate of conversion of (e) alkenyl bromide **1a**, (f) NHP ester **2b**, and (g) byproduct **4**. Concentrations determined vs dodecane internal standard using GC-FID.

Since the TDAE-driven **L1**·Ni-catalyzed ARA^6^ is homogeneous and does not suffer from an induction period, we initiated our mechanistic investigation by determining the kinetic orders in **1a**, **2b**, and Ni under standard reaction conditions (Figure 2a). For this, we employed variable time normalization analysis^12^ (VTNA) to analyze the results of different excess experiments (Figure 2b–d). These experiments revealed a first order rate dependence on the concentration of NHP ester **2b** (Figure 2c). The rate dependence on [**1a]** appears to be 0th order at concentrations similar to the standard conditions (0.1 and 0.2 M **1a**, Figure 2b); however, a fractional inverse rate dependence is observed at higher concentrations of **1a** (Figure 2b, Figure S27). Moreover, at higher [**1a]**, minor amounts of dienyl homodimer are observed; the slight inverse rate dependence is proposed to derive from this off-cycle pathway. We note that inverse order in C(sp^2^) electrophile has been observed previously by Weix et al. for a related (bpy)Ni-catalyzed RCC of aryl and alkyl halides.^9a^ Interestingly, there is an apparent 0th order rate dependence on **L1**·NiBr_2_ at loadings similar to the optimized conditions (5 and 10 mol %, Figure 2d); however, a positive rate dependence develops at low catalyst loadings (<1 mol %). The observation that the catalyst loading does not influence the rate of product formation has not been previously reported for Ni-catalyzed RCC reactions.^9b,10a^

To further investigate this unusual rate dependence, the concentration of **1a**, **2b**, and homodimer **4** were monitored over time, at different concentrations of Ni (Figure 2e-g). The conversion of alkenyl bromide **1a** shows a clear rate dependence on the concentration of Ni (Figure 2e). In contrast, the rate of conversion of NHP ester **2b** is independent of [Ni]: even in experiments where **L1**·Ni^II^Br_2_ is omitted, **2b** is consumed at the same rate as when using 20 mol % Ni (Figure 2f). Correspondingly, as the concentration of Ni decreases, the yield of cross-coupled product **3a** decreases and the yield of homocoupled product **4** (formed as a 1:1 diastereomeric mixture) increases (Figure 2g). These data are consistent with generation of a cage-escaped benzylic radical from **2b** by a non-Ni-catalyzed process. This represents a distinct mode of NHP ester activation for the **L1**·Ni-catalyzed RCC in comparison to the (bpy)Ni-mediated coupling of NHP esters reported by Weix et al.^13^ and Baran et al.,^14^ in which a (bpy)Ni^I^–Ar is proposed to reduce the NHP ester by single electron transfer (SET). In a preprint article, Rousseaux reported a similar finding, in which the combination of Zn^0^ and TMSCl initiates decarboxylation of an NHP ester as a part of a Ni-catalyzed RCC process.^15^

### 2.2 Reduction of Ni(II) Precatalyst and Oxidative Addition of Alkenyl Bromide

**Figure 3:**
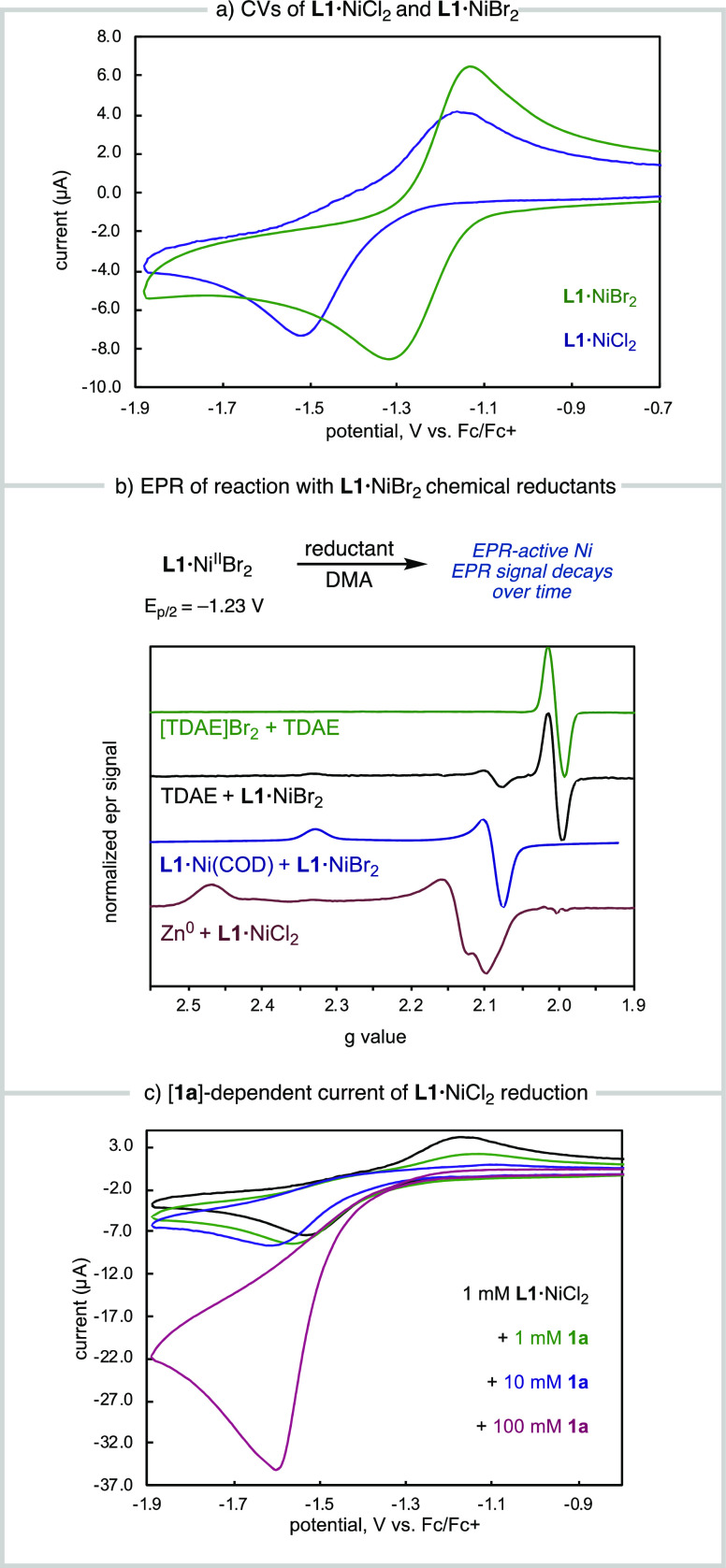
Reduction of precatalysts **L1**·NiX_2_ used in ARA reactions and reactivity of reduced species with **1a**. (a) 1 mM **L1**·NiBr_2_ and **L1**·NiCl_2_, 0.1 M TBAPF_6_ in DMA, ν = 100 mv/s, V vs Fc^0/+^; (b) −77 K X-band (9.4 GHz) perpendicular EPR spectra; for detailed instrument parameters and simulations, see SI Section 7.1. (c) 1 mM **L1**·NiCl_2_ with sequential additions of **1a** (0–0.1 M), 0.1 M TBAPF_6_ in DMA, ν = 100 mv/s. V vs Fc^0/+^.

Given the kinetic data (*vide supra*), we sought to investigate the reduction of the Ni precatalyst and the ability of the resulting species to oxidatively add the alkenyl bromide. We first used cyclic voltammetry (CV) to determine the reduction potentials of **L1**·Ni^II^Br_2_ and **L1**·Ni^II^Cl_2_; these complexes (isolable as crystalline solids) catalyze the reductive alkenylations of both benzylic chlorides and NHP esters in comparable yields and slightly improved ee relative to in situ catalyst generation.^6,7^ Electrochemically, **L1**·Ni^II^Cl_2_ and **L1**·Ni^II^Br_2_ exhibit irreversible reduction waves at *E*_p/2_ = −1.47 and −1.23 V vs Fc^0/+^, respectively (Figure 3a). These reduction events have a large peak separation with the corresponding oxidation events, suggesting that a chemical change, such as halide loss, occurs rapidly upon one electron reduction. More detailed electrochemical studies of these precatalysts, performed by Hadt and coworkers,^16^ support a single-electron reduction event to give a **L1**·Ni^I^X·DMA species. Notably, these studies suggest that reduction to **L1**·Ni^0^ does not proceed within the solvent window of DMA.

To verify the ability of TDAE to reduce **L1**·Ni^II^Br_2_ to **L1**·Ni^I^Br, a solution of **L1**·Ni^II^Br_2_ in DMA was treated with TDAE (*E*_1/2_ = −1.1 V vs Fc^0/+^); the resulting solution was frozen and analyzed by electron paramagnetic resonance (EPR) spectroscopy. A strong signal at g = 2.02 is assigned to the organic TDAE^+•^ radical,^17^ and the weaker signal (g_1_ = 2.07, g_2_ = 2.08, g_3_ = 2.330) is assigned to a reduced **L1**·Ni^I^Br species (Figure 3b, SI Section 7.3). The same **L1**·Ni^I^Br signal is observed when **L1**·Ni^II^Br_2_ is reduced with Ni(cod)_2_. When Zn^0^ is used as the reductant, more pronounced changes are observed, which could potentially arise from the interaction between a **L1**·Ni^I^ species with the Zn^II^ formed upon oxidation (Figure S69).^18^ We note that electrochemical and spectroscopic studies by Hadt and coworkers suggest that DMA can bind to both **L1**·Ni^I^ and **L1**·Ni^II^ redox states.^16^ Given the strong variation of EPR signals and speciation of **L1**·Ni^I^ species observed herein, no formal assignments of the EPR signals are provided. Nevertheless, these data support the presence of **L1**·Ni^I^X species forming from reduction under cross-coupling reaction conditions, and the nature of these species is clearly dependent on the reaction conditions.

A time course of the Zn^0^ reduction of **L1**·NiCl_2_ revealed that the observed EPR signals decrease over time (Figures S72–S74) in concert with a change in the corresponding UV–vis–NIR spectra (Figures S75 and S76), and the terminal EPR-silent mixture was catalytically inactive. Attempts to isolate **L1**·Ni^I^X complexes were unsuccessful; this might be due to the formation of **L1**·Ni^I^ oligomers in the absence of electrophiles **1** and **2** or due to the difference in stability between DMA-bound and unbound species.^10a,19,20^

To test whether the putative **L1**·Ni^I^Cl species formed upon reduction of **L1**·Ni^II^Cl_2_ can react with alkenyl bromide **1a**, a series of CV studies were performed in the presence of **1a** (Figure 3c). A concentration-dependent increase in current was observed as [**1a**] increased, which was accompanied by a loss of reoxidation current. Taken together, these studies are consistent with reaction between **L1**·Ni^I^X and alkenyl bromide **1a**.

### 2.3 Catalyst Resting State

At this stage, we sought to determine the resting state of the Ni catalyst under the reaction conditions. If a Ni^I^ or Ni^III^ intermediate were the resting state, then it could be observable by EPR. The Ni-catalyzed reaction of **1a** and **2b** was performed using 2 mol % **L1**·NiBr_2_ under otherwise standard conditions, and aliquots were removed, filtered, and frozen in an EPR tube. No signal corresponding to a metal-based radical was observed by EPR; instead, a signal consistent with an organic radical was observed, which decreased in intensity over time (Figure S77). This species was assigned as the TDAE radical cation by comparison to an independently prepared sample (Figure S67) and previously reported spectra.^16^ Although this does not rule out a Ni^I^ or Ni^III^ resting state, we sought to investigate other possibilities.

**Figure 4:**
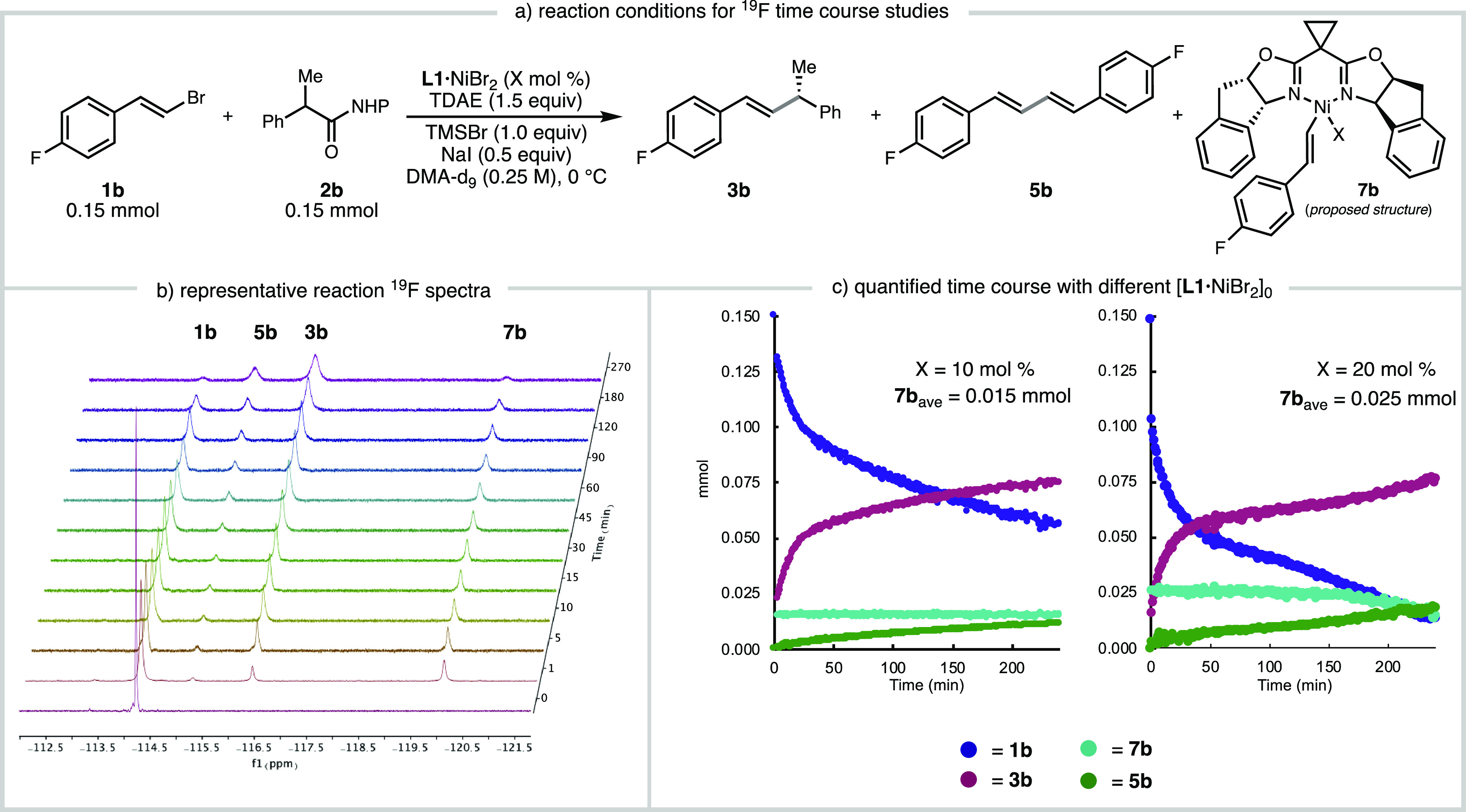
Reaction monitoring by ^19^F NMR tracking the consumption of **1b** and the formation of **1b**-derived species. The reaction was run at 0 °C using C_6_F_6_ as an internal standard.

Given the rapid reaction of **L1**·NiCl with alkenyl bromide **1a** (Figure 3c) and prior RCC mechanistic studies,^9b,10a^ we hypothesized that the catalyst resting state likely resides after oxidative addition of the C(sp^2^)-electrophile. To monitor the reaction by *in situ*^19^F NMR, ^19^F-labeled alkenyl bromide **1b** was used and all alkenyl bromide-derived species were tracked over the course of the reaction (Figure 4a). Upon the addition of TDAE (0. 23 mmol, 1.5 equiv) to a solution of **1b** (0.15 mmol), **2b** (0.15 mmol), **L1**·NiBr_2_ (0.015 mmol, 10 mol %), TMSBr (0.15 mmol), and NaI (0.075 mmol) in DMA-*d*_9_, several new signals appeared that were assigned to product **3b** (δ = −116.5 ppm), homocoupled diene **5b** (δ = −115 ppm), and a new species (broad signal at δ = −120 ppm, Figure 4b).^21^ This species persisted throughout the reaction, maintaining steady concentration corresponding to 15 μmol or 10 mol %, which is the concentration of **L1**·NiBr_2_ used in the reaction. When this experiment was repeated with 20 mol % **L1**·NiBr_2_, the concentration of this species corresponded to 17 mol % (25 μmol) for the first 2.5 h of catalysis and then decreased as the reaction approached the last few turnovers, eventually disappearing at the end of the reaction (Figure 4c). Although attempts to isolate this species or prepare it independently have been unsuccessful due to its instability, we propose that this intermediate is the diamagnetic Ni^II^ oxidative addition complex **7b**.^22^ We note that Diao and coworkers reported a dimagnetic (phen)Ni^II^ArBr resting state, observable by ^1^H NMR, for a Ni-catalyzed 1,2-dicarbofunctionalization reaction.^10b^

### 2.4 Mechanism of NHP Ester Activation

**Figure 5:**
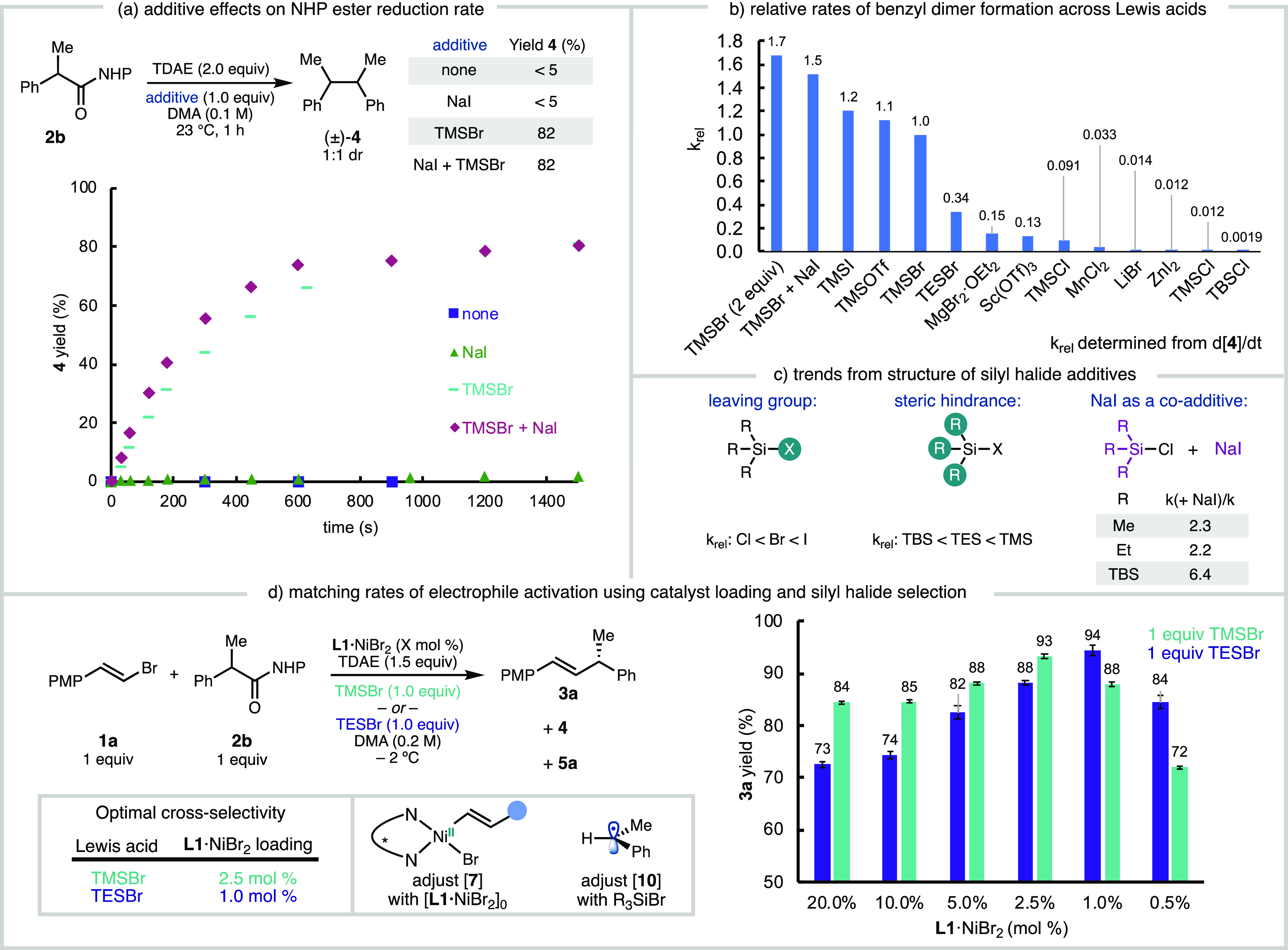
Studies investigating the influence of Lewis acids on the rate of radical generation from TDAE reduction of **2b**. Yields and time course data analyzed by GC-FID with *n*-dodecane internal standard.

Given that the kinetic studies revealed that the NHP ester **2b** is not reduced by Ni, we hypothesized that it is instead reduced by TDAE. To test this hypothesis, NHP ester **2b** was treated with TDAE in DMA and the formation of homodimer **4** was monitored as an indirect measurement of benzylic radical generation. In the absence of additional additives, the mixture of **2b** and TDAE results in minimal conversion to homodimer **4**, even at ambient temperature (Figure 5a, purple). This can be rationalized by the difference in reduction potential of NHP ester **2b** (*E*_p/2_ = −1.62 V vs Fc^0/+^), which is 0.5 V more cathodic than TDAE (*E*_1/2_ = −1.11 V vs Fc^0/+^); the irreversible loss of CO_2_ following SET does not appear to be sufficient to drive the thermodynamically unfavorable process. Similarly, the mixture of **2b**, TDAE, and NaI also fail to produce homodimer **4** (Figure 5a, green).

In contrast, when TDAE is added to a mixture of **2b** and TMSBr (1.0 equiv), **2b** is converted to **4** at a rate that is comparable to the rate of **2b** conversion in the catalytic reaction (Figure 5a, teal). We note that TMSBr is essential to form **3a** in high yields under standard reaction conditions (19% yield **3a** when TMSBr is excluded). The rate is increased further (*k*_rel_ = 1.5) when both TMSBr and NaI are present, presumably through the *in situ* generation of TMSI (Figure 5a, maroon). We propose that the silyl halide additive functions as a Lewis acid to lower the reduction potential^23^ of the NHP ester and enable reduction by TDAE.

### 2.5 Altering Rate of Radical Generation through Lewis Acid Selection

**Figure 6:**
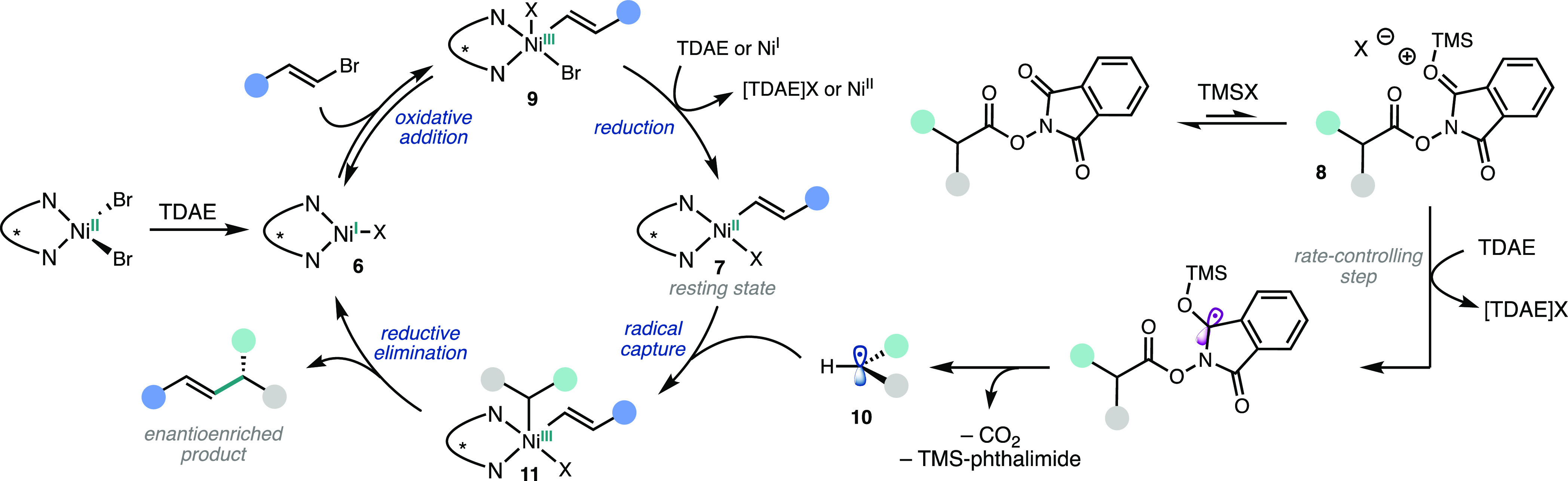
Proposed mechanism for the TDAE-mediated Ni-catalyzed coupling between NHP esters and alkenyl bromides under standard conditions (see Figure 2a).

The observation that a Lewis acid gates NHP ester reduction inspired us to question whether the rate of radical generation could be tuned by using TDAE in combination with different Lewis acids, similar to Weix and coworkers’ work tuning the rate of radical generation by using derivatized NHP esters.^24^ To test this, we measured the rate of radical generation (as d[**4**]/d*t*) in the presence of a variety of Lewis acids (Figure 5b). The more sterically hindered triethylsilyl bromide (TESBr) results in a 3-fold decrease in the rate (*k*_rel_ = 0.34) of radical generation. Further investigation of different silyl halides revealed an intuitive trend in sterics (TBS < TES < TMS), with larger groups slowing down radical generation, as well as the leaving group identity (Cl < Br < OTf < I),^25^ with the better leaving group accelerating radical generation (Figure 5c). As observed with TMSBr (Figure 5a, purple), addition of NaI as a co-additive to various R_3_Si–Cl additives can increase the rate by more than 2-fold. Increasing the concentration of TMSBr increases the rate (*k*_rel_ = 1.68), presumably by driving the equilibrium to increase the concentration of silylated NHP ester **8** (Figure 6). Additionally, non-silyl Lewis acids can also increase the rate of **2b** reduction by TDAE. We have quantified the ability of several common additives^26^ to modulate the rate of radical generation, with rates spanning three orders of magnitude (Figure 5b).

After demonstrating that the rate of NHP ester activation can be tuned with different Lewis acid additives, we sought to investigate how the yield of product was effected by the silyl additive. Although the ARA reaction between **1a** and **2b** was initially reported using TMSBr, we observed that at lower catalyst loadings, increased amounts of benzyl dimer **4** are formed (Figure 2g). We hypothesized that a slower rate of radical generation could improve the yield of **3a** at low catalyst loadings by better matching of the relative concentrations of the resting state species (**7**) and benzylic radical. Given that TESBr decreases the rate of benzylic radical formation by 3-fold, we performed a series of experiments varying the concentration of **L1**·NiBr_2_ in the presence of either TMSBr or TESBr (Figure 5d). First, we note that for this well-performing substrate pair, high yields can be maintained using 1 mol % **L1·**NiBr_2_. Second, we found that TMSBr performs better relative to TESBr when 20 mol % **L1·**NiBr_2_ is used (higher concentration of resting state **7**) and performs worse than TESBr at 0.5 mol % **L1·**NiBr_2_, when rapid release of benzyl radical would outpace radical capture by resting state **7** (Figure 5d). Using 0.5 mol % **L1·**NiBr_2_, higher yield of **3a** was obtained with TESBr (84% yield) than with TMSBr (72% yield), which we propose results from the slower release of the benzyl radical. Analysis of the product profiles for each bromosilane shows that the ratio of cross-coupled to homocoupled products reaches a maxima at 2.5 mol % Ni for TMSBr (*k*_rel_ = 1.0) and 1 mol % Ni for TESBr (*k*_rel_ = 0.34) (Figures S78 and S79), which is consistent with the trends observed in yield. Using these conditions (1 mol % **L1·**NiBr_2_ and 1.0 equiv TESBr), the reaction can be performed on the gram scale to give product **3a** in 81% yield and 94% ee (SI Section 8.2). We propose that monitoring the conversion of starting materials as well as the yield of product and alkyl homodimer at different [Ni] can help chemists identifying the optimal combination of catalyst loading and silyl additive for new substrate combinations.

Taken together, a mechanism for the TDAE-mediated Ni-catalyzed RCC is proposed in Figure 6. Upon reduction of the Ni precatalyst, the resulting **L1·**Ni^I^Br (**6-Br**) rapidly reacts with alkenyl bromide to give Ni^III^ species **9**, which can be reduced to furnish resting state species **7**. Given Hadt and coworker’s studies,^16^ it is possible that DMA is coordinated to **6** during oxidative addition. While the reductant in this oxidative addition–reduction sequence is not known, we propose that the oxidative addition step is fast and reversible since we can observe the formation of halide scrambling products **1a-I** and **1a-Cl** (when a Cl^–^ source is present).^7,27^ The Ni^II^ complex **7** can then intercept NHP ester-derived radical **10** to give Ni^III^ complex **11**, which can undergo reductive elimination to give product **3**. NHP ester **2b** is activated by TMSBr followed by reduction with TDAE in the turnover-limiting step. This reduced species undergoes N–O homolysis and subsequent decarboxylation to give **10** (Figure 6).

### 2.6 Reaction Kinetics of the Mn-Mediated Ni-Catalyzed RCC

**Figure 7:**
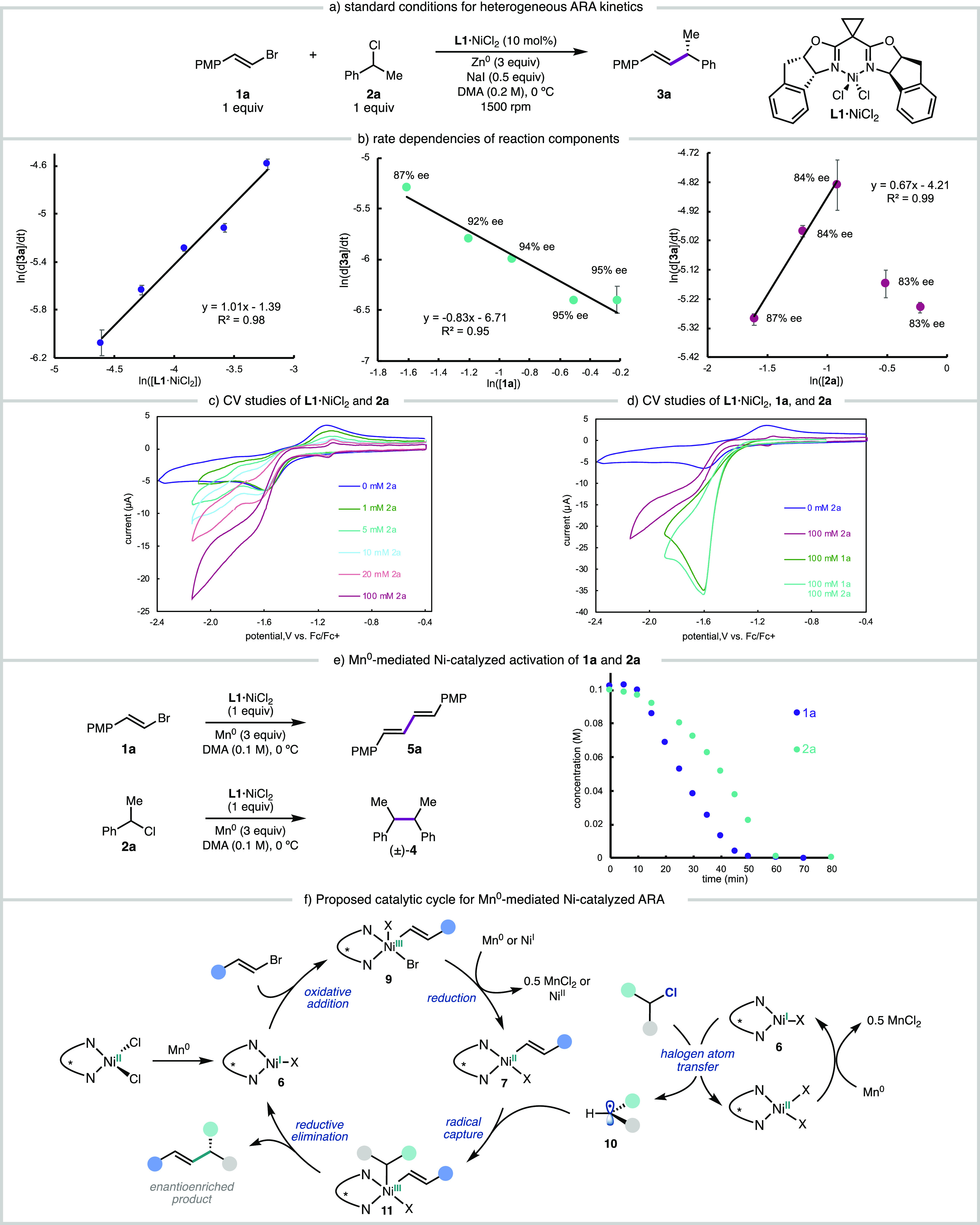
(a) Standard conditions used the Zn-mediated ARA kinetics experiments. (b) Kinetics experiments varying initial concentrations of **1a**, benzyl chloride **2a**, and catalyst **L1**·NiCl_2_. (c) CV studies of sequential addition of **2a** (0–0.1 M) to 1 mM **L1**·NiCl_2_. (d) CV studies **L1**·NiCl_2_ reacting with either **1a** or **2a** or both **1a** and **2a** (0.1 M TBAPF_6_ in DMA, ν = 100 mv/s. V vs Fc0/+). (e) Mn-mediated stoichiometric reactions of **L1**·NiCl_2_ with **1a** and with **2a**. (f) Proposed mechanism for the Mn-mediated Ni-catalyzed coupling between benzyl chlorides and alkenyl bromides. Concentrations determined by GC-FID vs dodecane internal standard.

Kinetic studies of the heterogeneous metal-powder conditions (Figure 1**a**) proved more challenging than the homogeneous TDAE-mediated reaction. We observe long induction periods (up to 90 min) and reaction times of 6 h using previously reported conditions.^6^ The induction period and reaction times can be shortened to 30 and 100 min, respectively, by preactivating the Mn^0^ with HCl. The use of Zn^0^ powder further improved the reaction times (5–10 min induction period and 45 min reaction times, Figure S16) and provided a product in comparable yield and with only slightly lower enantioselectivity as Mn^0^ (Zn: 91% yield, 90% ee; Mn: 96% yield, 96% ee). Both Mn^0^ and Zn^0^ gave reactions with linear rates of product formation, indicative of mass transport-limited reduction; however, Zn^0^ displayed a less significant stir rate dependence that saturated >1000 rpm (Figure S15). The use of 6 equiv Zn^0^ slightly increased the reaction rate by a factor of 1.1, similar to observations by Weix and Biswas^9b^ and Diao and Lin^10a^ in related arylation reactions (Figure S17). These modified reaction conditions (Figure 7a) enabled the collection of reproducible kinetic data allowing us to kinetically observe the next slowest step after mass transported-limited heterogeneous reduction.^9a,28^

Kinetics experiments reveal a first-order rate dependence on [**L1·**NiCl_2_], unlike the TDAE system, across catalyst loadings ranging from 5 to 20 mol % (Figure 7b). The reaction exhibits a negative first-order rate dependence on [**1a**]_0_. A similar inverse dependence on the C(sp)^2^ partner has been observed by Weix and Biswas.^9b^ The rate dependence on [**2a**]_0_ is more complex: a fractional positive rate dependence was observed at 1.0 and 2.0 equivalents of **2a**, but the rate decreases again when >2.0 equiv **2a** is employed (Figure 7b). Notably, as [**2a**]_0_ increases, the ee of **3a** decreases. Taken together, these data might indicate that there are competing mechanisms that depend on the concentration of [**2a**]_0_. One possibility is that when [**2a**] ≫ [**1a**], the reaction of **L1**·Ni^I^X with **2a** begins to compete with the reaction between **L1**·Ni^I^X and **1a**, therefore reversing the order of oxidative addition of the electrophiles to Ni.

### 2.7 Ni-Mediated Electrophile Activation

To interrogate the role of Ni in the activation of benzylic chloride **2a**, a DMA solution of **2a** was treated with Mn^0^ (3.0 equiv) and NaI (0.5 equiv) and the formation of homodimer **4** was monitored (see SI, Section 4.3). No conversion of **2a** or formation of **4** was observed at 0 or 23 °C, even with extended reaction times. In contrast, when **2a** was subjected to identical conditions but **L1**·Ni^II^Cl_2_ was added, **2a** was cleanly converted to homodimer **4** over 60 min (Figure 7e). These findings suggest that **L1**·Ni^I^X can activate **2a**.

CV studies were also performed to investigate the reaction of in situ generated **L1**·Ni^I^Cl with **1a** and **2a**. CVs were acquired for **L1**·Ni^II^Cl_2_ (1.0 mM) in the presence of varying concentrations of **1a** (1–100 mM), which showed a concentration-dependent current with cathodic shifting of the onset potential and loss of the anodic return wave (Figure 7c). This current likely results from the reaction of the reduced **L1**·Ni complex reacting with **2a**, presumably corresponding to the catalytic homocoupling to give **4**. The same studies were performed in the presence of alkenyl bromide **1a**, which also showed a concentration-dependent current (Figure 3c). In the presence of 100 mM **1a** and **2a**, regardless of the order of addition, a catalytic current consistent with the reaction with **1a** is observed (Figure 7d).

This is consistent with the Mn-mediated Ni-catalyzed homodimerization reactions of **1a** and **2a**, in which the conversion of **1a** is faster than the conversion of **2a** under otherwise identical conditions (Figure 7e). Taken together with the CV studies, these data qualitatively suggests that the reductively generated **L1**·Ni^I^Cl reacts faster with **1a** and is consistent with previous RCC studies investigating the relative rates of Ni(I) complexes with aryl and alkyl electrophiles.^9b,10a,12^

Based on our experimental studies, a proposed mechanism for the Mn-mediated Ni-catalyzed ARA is shown in Figure 7f. Upon reduction of precatalyst **L1**·Ni^II^Cl_2_, the resulting complex **6** reacts with alkenyl bromide **1** in an oxidative addition–eduction step to give **L1·**Ni(II) complex **7**. This could proceed by a bimolecular oxidative addition as proposed by Diao and Lin,^10a^ or by reduction of the transiently formed Ni(III) species by Mn^0^. Ni-catalyzed halogen atom transfer (XAT)^29^ from the benzylic chloride gives rise to a cage-escaped radical **10** that can be captured by **7** to yield product **3** following reductive elimination. We again note that both **6** and **7** are in equilibrium with DMA-bound complexes,^14^ and DMA coordination might facilitate the oxidative addition of **1** or XAT from **2a.**

The mechanism shown in Figure 7f is consistent with our observation that **L1**·Ni^I^X (**6**) can react with both **1a** and **2a** but that **1a** reacts with **6** more rapidly. This mechanism is also consistent with the observed inverse dependence on [**1a**]_0_: complex **6** is partitioned between two processes. When **1a** reacts with **6**, it effectively reduces the concentration of **6** available to react with **2a**. If benzylic radical generation by the reaction of **6** with **2a** is the rate-controlling step (the studies in Figure 7e show that reaction of **6** with **2a** is slower than with **1a**), then increased [**1a**] would be expected to decrease the rate of reaction between **6** and **2a** and therefore the overall rate of product formation. We note that we cannot rule out the possibility that **7** is reduced and that the corresponding **L1**·Ni^I^(alkenyl) species, which is calculated to be a stronger reductant,^16^ mediates the XAT; however, we would not expect a significant inverse rate dependence on **1a** for such a process. In addition, recent studies by Diao and coworkers have suggested that similar (biox)Ni^II^(aryl)X complexes are unlikely to be reduced by Mn^0^.^10b^

### 2.8 Computational Investigation into the Origin of Enantioselectivity

**Figure 8:**
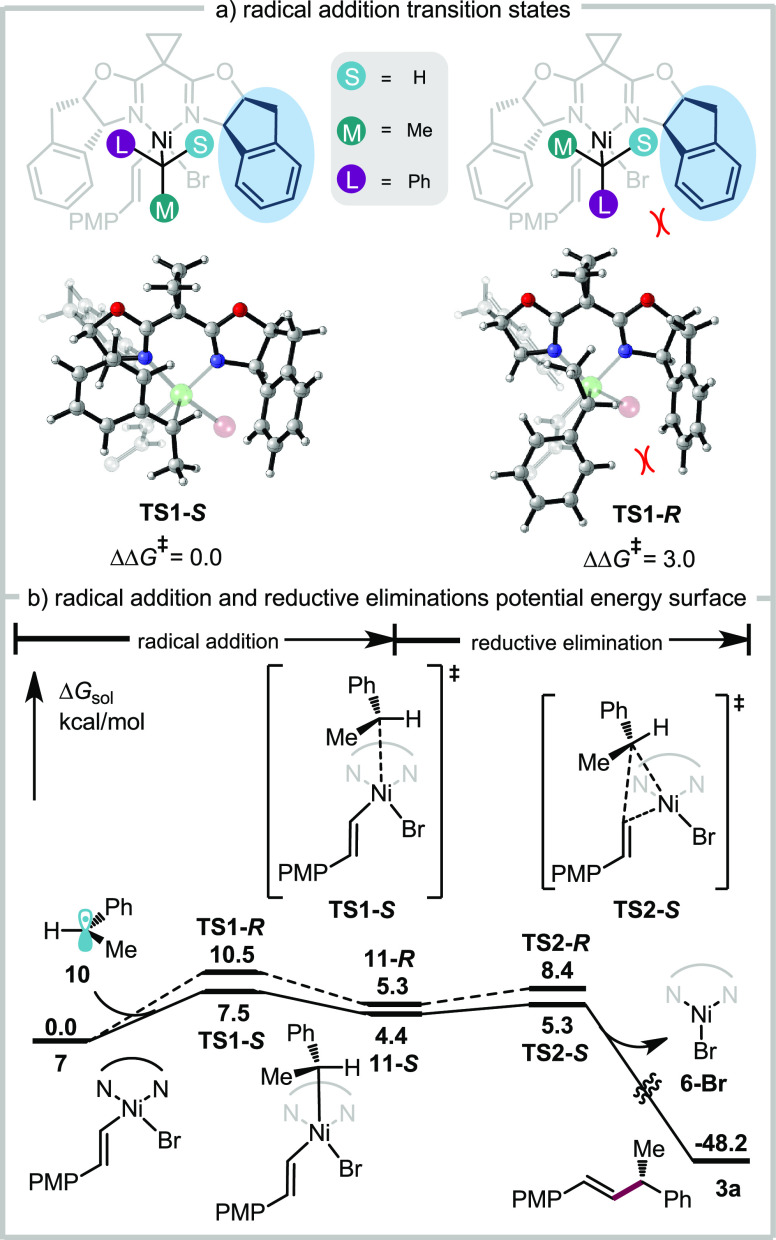
Calculated potential energy surface of enantiodetermining radical capture step and subsequent reductive elimination.

To explore the origins of enantioinduction, the structures and relative Gibbs free energies of the competing transition states for addition of radical **10** to resting state complex **7** were computed (Figure 8a). The free energy difference between **TS1-***S* and **TS1-***R* is computed to be 3.0 kcal/mol, which slightly overestimates the enantioselectivity for the reaction. In both transition states, the smallest substituent of the approaching benzyl radical (**10**), hydrogen, is pointing toward the sterically bulky part of the ligand (highlighted in blue in Figure 8a). This allows the largest substituent, the phenyl group, to project away from this region of the ligand in the favored transition state **TS1-***S*. In the disfavored transition state **TS1-***R*, the phenyl group is proximal to the bulky region of the ligand. This results in an almost perfectly staggered approach of the benzyl radical with respect to the Ni ligands in **TS2-***S*, while steric repulsion from the ligand forces the benzyl radical to adopt a more eclipsed conformation in **TS1-***R*.^30^ Subsequent reductive elimination for **11-***S* is facile with a computed barrier of 0.9 kcal/mol (Figure 8b) for the major pathway. This is in contrast to previously reported computational results for a related Ni-photoredox coupling, which proposed a reversible radical addition and enantiodetermining reductive elimination;^31^ however, this mechanism is consistent with recently published experimental results that measure a rapid reductive elimination from a Ni^III^ complex that is analogous to **11**.^32^ These calculations suggest the facial selectivity of the enantiodetermining radical addition is influenced by the steric environment of BOX ligand **L1**.

## 3 Conclusions

In summary, we have investigated two Ni-catalyzed asymmetric RCC reactions to determine how changing the reductant and C(sp^3^) electrophile influences the reaction mechanism. These reactions proceed through a Ni^I/III^ cycle with fast activation of the alkenyl bromide electrophile by a Ni^I^ species. Both reactions have a rate-determining activation of the C(sp^3^) electrophile to furnish a cage-escaped benzyl radical. We have demonstrated that Ni is not required for NHP ester activation; instead, the combination of TDAE and TMSBr results in reductive decarboxylation to give the benzylic radical. The radical can then be intercepted by a Ni^II^–alkenyl resting state that we were able to detect spectroscopically. In the case of the Mn^0^-mediated ARA with benzylic chlorides, we propose that **L1**·Ni^I^X generates the benzylic radical by an XAT process. This is a subtle distinction from the mechanism proposed by Diao for the (biox)Ni-catalyzed RCC between aryl halides and benzylic chlorides,^10b^ which did not suggest an explicit role for Ni in generating the radical from the benzylic halide.

The fact that reduction of NHP esters by TDAE is Lewis acid-mediated, rate controlling, and independent of the alkenyl bromide activation has significant implications for the development of other Csp^3^–Csp^*n*^ RCCs. This mechanistic regime allows for independent tuning of the rates of electrophile activation where d[Csp^3^]/dt can be tuned with additives and d[Csp^2^]/dt through catalyst design. It is our hope that these findings aid in the adoption of C(sp^2^)–X reductive couplings with NHP ester fragments in more complex settings by providing a framework to guide reaction optimization.

## Supporting Information Available

Experimental procedures, characterization data (^1^H and ^19^F NMR) for all new compounds, and coordination geometries for DFT optimized compounds (PDF)
