# Supplementary material for: Mechanistic Investigation of Ni-Catalyzed Reductive Cross-Coupling of Alkenyl and Benzyl Electrophiles
Source: J Am Chem Soc. 2023 Jun 26;145(27):14705–15. doi: 10.1021/jacs.3c02649 (PMC10347553; doi:10.1021/jacs.3c02649)
Supplement: Supplementary file 1 — ja3c02649_si_001.pdf [file ja3c02649_si_001.pdf]

Mechanistic Investigation of Ni-Catalyzed Reductive Cross-Coupling of Alkenyl and Benzyl Electrophiles

Raymond F. Turro, Julie L.H. Wahlman, Z. Jaron Tong, Xiahe Chen, Miao Yang, Emily P. Chen, Xin Hong,[4] Ryan G. Hadt,[2] K. N. Houk,[5] Yun-Fan Yang,[3]* Sarah E. Reisman[1]*

[1]The Warren and Katharine Schlinger Laboratory for Chemistry and Chemical Engineering, Division of Chemistry and Chemical Engineering, California Institute of Technology, Pasadena, California 91125, United States

[2]Arthur Amos Noyes Laboratory of Chemical Physics, Division of Chemistry and Chemical Engineering, California Institute of Technology, Pasadena, California, 91125, United States

[3]College of Chemical Engineering, Zhejiang University of Technology, Hangzhou, Zhejiang 310014, China

[4]Department of Chemistry, Zhejiang University, Hangzhou 310027, China

[5]Department of Chemistry and Biochemistry, University of California, Los Angeles, California 90095, United States

**reisman@caltech.edu*

Supporting Information

Table of Contents

1. Experimental Details S3

2. Synthetic Procedures S4

3. Kinetics and Time Course Experiments S5

   3.1. Heterogenous Reaction Kinetics S5

   3.2. Homogeneous Reaction Kinetics S13

4. Mechanism of Substrate Activation S23

4.1. Additive Effects on NHP Ester Reduction Rate S23

4.2. Comparison of **1a** and **2a** Activation Rates by **L1**·$NiCl_2$ S35

4.3. Catalyst-Mediated **1a** Activation Control Experiments S37

4.4. Preliminary Kinetic Simulations of Rate-Controlling Activation of NHP esters S38

5. Cyclic Voltammetry Experiments S40

5.1. CV's of **L1**·NiX_2, **2b**, and TDAE S40

5.2. Substrate Titration and Catalytic Current Comparison S44

6. NMR Reaction Monitoring S45

6.1. ^{19}FNMR Reaction Monitoring S45

6.2. Room Temperature ^{1}H and ^{19}F NMR S50

6.3. Attempts to assign **7b** with ^{1}H NMR S51

7. Electron Paramagnetic Spectroscopy (EPR) S61

7.1. Generation of Ni(I) from Chemical Reduction of **L1**·NiX_2 .... S61

7.2. Reaction of **L1**·Ni(COD) with **1a** S64

7.3. Reduction of **L1**·$NiCl_2$ with Zn^0 Time Course S66

7.4. Reaction Monitoring with EPR S69

8. Catalyst Loading Experiments (Figure 5d) S70

9. Computational Data S71

9.1. DFT-Computed Gibbs Free Energy Barriers for Radical Addition and Reductive Elimination S75

9.2. Table of Energies S76

9.3. Cartesian Coordinates for Calculated Species S77

10. References S109

1. Experimental Details

Materials and Methods

Unless otherwise stated, reactions were performed under a N_2 atmosphere using freshly dried solvents. All reagents were purchased from commercial suppliers (Sigma Aldrich, Combi-Blocks, TCI, Enamine, Strem) and used without further purification unless mentioned otherwise. Tetrahydrofuran (THF), acetonitrile (MeCN), and methylene chloride (CH_2Cl_2) were dried by passing through activated alumina columns. Anhydrous dimethylacetamide (DMA) was purchased from Aldrich and stored in a N_2-filled glovebox. $NiCl_2$·dme was purchased from Strem and stored in the glovebox. Manganese powder (~325 mesh, 99.3%) was purchased from Alfa Aesar. Zinc dust (97.5%) was purchased from Strem. NaI (anhydrous, 99%) was purchased from Strem and stored in a N_2-filed glovebox. Flash column chromatography was performed as described by Still et al. using silica gel (230-400 mesh, Silicycle).[1] Purified compounds were dried on a high vacuum line (0.2 torr) to remove trace solvent. ^{1}H and ^{13}C NMR spectra were recorded on a Bruker Avance III HD with Prodigy cryoprobe (at 400 MHz and 101 MHz, respectively), a Varian 400 MR (at 400 MHz and 101 MHz, respectively), or a Varian Inova 500 (at 500 MHz and 126 MHz, respectively). ^{1}H and ^{19}F NMR spectra were also recorded on a Varian Inova 300 (at 300 MHz and 282 MHz, respectively). NMR data is reported relative to internal $CHCl_3$ (^{1}H, δ = 7.26) and $CDCl_3$ (^{13}C, δ = 77.0) or C_6F_6 (^{19}F -164.9 ppm). HRMS were acquired from the Caltech Center for Catalysis and Chemical Synthesis Facility using electrospray ionization (ESI-TOF). Analytical chiral SFC was performed with a Mettler SFC supercritical CO_2 analytical chromatography system with Chiralcel AD-H, OD-H, AS-H, OB-H, and OJ-H columns (4.6 mm x 25 cm). Analytical achiral GC was performed with an Agilent 6850 GC utilizing an HP-1 capillary column (methyl siloxane, 30.0 m x 320 µm x 0.25 µm, Agilent) column with a splitless injection and a helium flow of 7.3 mL/min. The temperature program began at 50 °C and was held for 2 min, increased to 250 °C at 25 °C/min and then held at 250 °C for 3 min. X-band perpendicular mode EPR spectra were recorded on a Bruker EMX spectrometer at 77 K using a LN_2 immersion dewar. Parallel mode EPR were recorded at 5K using a LHe cryostat. EPR spectra were simulated with Easyspin (version 5.2.35)[2]. Electronic absorption spectra were obtained using CARY 300 spectrophotometer. Electroanalytical experiments were conducted in the Beckman Resource Laser Resource Center at the California Institute of Technology using a Bio-Logic SP300 potentiostat/galvanostat. Cyclic voltammetry experiments we conducted with a glassy carbon

disk working electrode, a platinum wire counter electrode, and a silver wire reference electrode containing a 10 mM $AgNO_3$ solution with 0.1 M $TBAPF_6$ in MeCN.

2. Synthetic Procedures

Substrate and Catalyst Synthesis

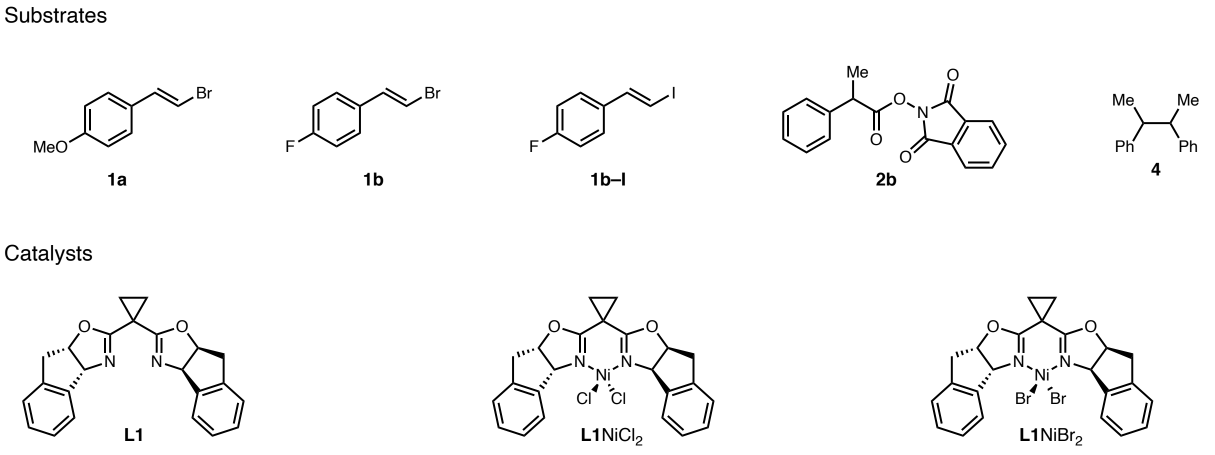

Figure S1. Substrates and catalysts used for mechanistic studies.

Catalysts:(3a*R*,3a'*R*,8a*S*,8a'*S*)-2,2'-(Cyclopropane-1,1-diyl)bis(3a,8a-dihydro-8*H*-indeno[1,2-*d*]-oxazole) **(L1)** was synthesized according to our previously published procedure.[3] Complexation with $NiBr_2$ or $NiCl_2$ were prepared according to previously reported synthesis of **L1**$NiCl_2$[4] and **L1**$NiBr_2$.[5] Complexes were recrystallized once by vapor diffusion of pentane in a saturated DCM solution for use in catalytic reactions and 3 times for use in electroanalytical experiments.

Substrates: Coupling partners **1a**, **1b** and **2b** were synthesized according to the procedure described in the initial disclosure.[5]

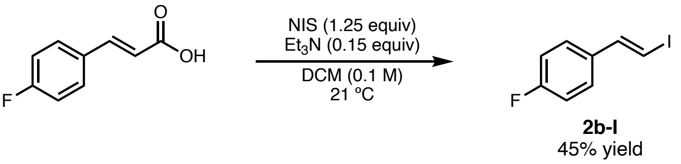

(*E*)-1-fluoro-4-(2-iodovinyl)benzene (**2b-I**): To a 250 mL oven-dried round bottom flask with a stir bar was added (*E*)-4-fluorocinnamic acid (831.6mg, 5.0 mmol, 1 equiv). The acid was then suspended in 50 mL (0.1 M) DCM then triethylamine (105 µL, 0.75 mmol, 0.15 equiv) was added and the reaction was stirred under N_2. To the stirring solution was then added *N*-iodosuccinimide (1.41 g, 6.25 mmol, 1.25 equiv) in one portion. After 12 minutes, the reaction solution had turned red and then deep black after 20 minutes. After 1h the starting material was consumed by TLC and the reaction mixture was concentrated *in vacuo*. Residue was taken up

in 30 mL EtOAc and washed with 25 mL sat. $Na_2S_2O_3$ solution. The aqueous layer was then extracted two more times and combined organics were dried over $MgSO_4$, filtered through celite, rinsed with EtOAc then concentrated in vacuo to give a brown solid. The crude was then purified by filtration through SiO_2 with pentane to give alkenyl iodide **2b-I** (558 mg, 2.3 mmol, 45% yield). *Note:* we observed significant discoloration and decomposition upon prolonged exposure to light so storage at -20 ºC in the darkness under Ar is essential to prevent decomposition. Spectral data is in good agreement with literature reported data.[6]

3. Kinetics and Time Course Experiments

Methods of GC-FID Quantification: For each reaction component and product, authentic samples were isolated to determine response factors for GC-FID analysis. Three standards were made for each analyte to normalize the GC-FID area counts and convert the obtained data into reaction concentration (M) values. The analyte and dodecane standard were each added to a 20 mL vial and massed on a balance. The mixture was dissolved in 10 mL of EtOAc and transferred to a GC vial for analysis. The density of dodecane (0.75 g/mL) was also used to convert the area values to concentration.

3.1 Heterogenous Reaction Kinetics

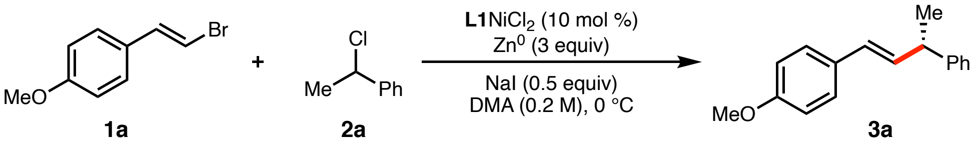

General Procedure 1 (Zn^0 powder): A 10 mL round bottom flask with a small magnetic stirring rod was charged with the sodium iodide (22.5 mg, 0.15 mmol, 0.5 equiv) and zinc powder (58.8 mg, 0.9 mmol, 3 equiv). The flask was sealed with a rubber septum, purged with N_2, and cooled to 0 °C by being placed in an ice water bath. The alkenyl bromide **1a** (85.2 mg, 0.4 mmol) and **L**·$NiCl_2$ complex (19.4 mg, 0.04 mmol) were added to a 2 mL volumetric flask, sealed with a rubber septum, and purged with N_2. The benzyl chloride **2a** (53 µL, 0.4 mmol) and dodecane (48 µL) as an internal standard were added via syringe to the volumetric flask. Then anhydrous DMA was added to the volumetric flask until it reached the 2 mL line. A small stir bar was added to the volumetric flask and the solution was stirred until all of the **L**·$NiCl_2$ complex was dissolved. The solution was taken up into a 2 mL syringe to ensure homogeneity, and then 1.5 mL of the solution was added to the round bottom flask. The reaction was stirred under N_2 by inlet needle attached to a Schenck line by using an IKA stir plate set to a stirring

speed of 1500 rpm. At appropriate time points, approximately 50 μL of the solution was removed by syringe (syringe and needle were pre-flushed with N_2), loaded onto a short silica plug (1 cm) in a glass pipette packed with cotton. The crude mixture was flushed through the silica plug with 2 mL of 10% EtOAc/hexane directly into GC vials and analyzed by GC-FID. All data runs obtained from the GC-FID instrument were appropriately integrated for the product and the dodecane standard. The integrated data points were further processed by normalizing each product area value by its corresponding standard area value. The normalized areas were then converted to concentration by using calculated response factors obtained from preparing known mixtures of the standard and purified reaction product. Each reaction was analyzed and graphed to show the product concentration (M) as a function of reaction time (min). All data points were plotted with black markers (•) as shown below, while only the data points included in the linear fit are shown with red markers (•). The best-fit linear regression line is also shown and the y=mx+b equation is given. Each reaction was run in duplicates as indicated by Trial 1 and Trial 2.

Standard Reaction Conditions

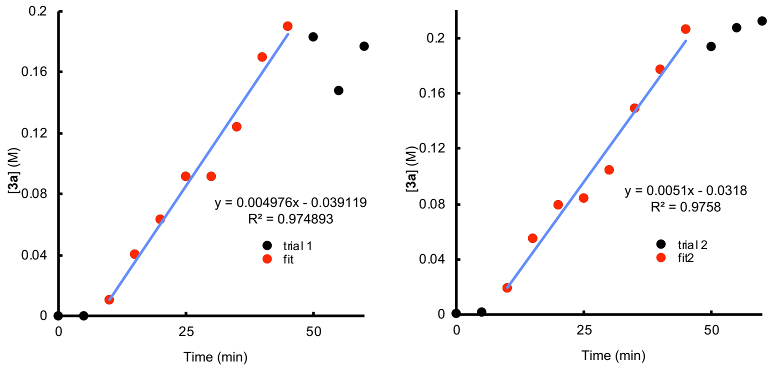

Figure S2: Results of standard reaction conditions runs with linear fit regions highlighted: [**1a**]$_0$ = 0.2M, [**2a**]$_0$ = 0.2M, [**L1**·$NiCl_2$] = 0.02M.

Effect of Changing [L1·$NiCl_2$]

The general procedure 1 was followed except varying the amounts of **L1**$NiCl_2$ were used to give final loadings of 5%, 7%, 14%, and 20%.

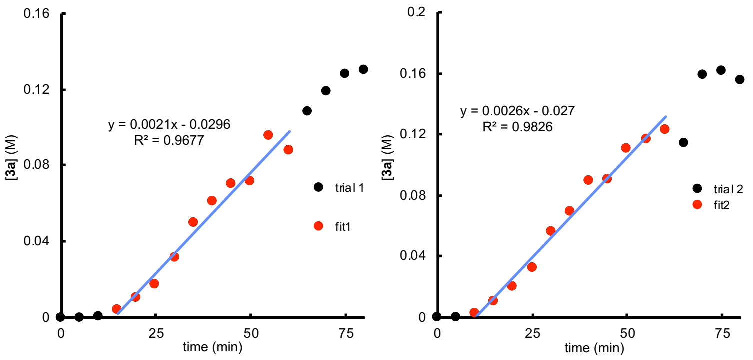

Figure S3: Kinetics runs with linear fit regions highlighted: [**1a**]$_0$ = 0.2M, [**2a**]$_0$ = 0.2M, [**L1**·$NiCl_2$] = 0.01M.

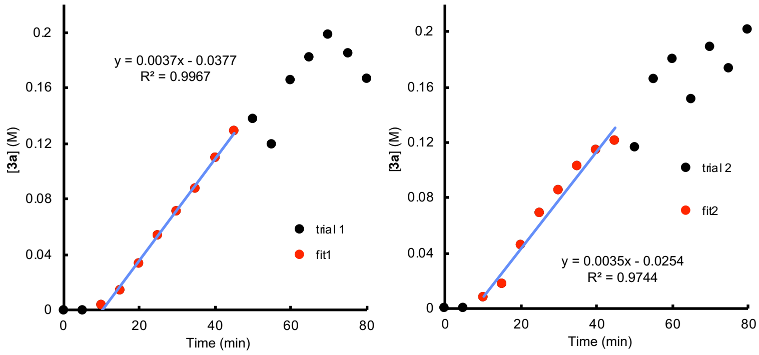

Figure S4: Kinetics runs with linear fit regions highlighted: [**1a**]$_0$ = 0.2M, [**2a**]$_0$ = 0.2M, [**L1**·$NiCl_2$] = 0.014M.

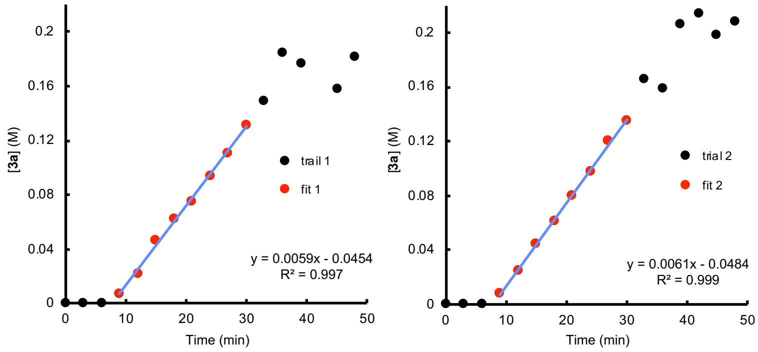

Figure S5: Kinetics runs with linear fit regions highlighted: [**1a**]$_0$ = 0.2M, [**2a**]$_0$ = 0.2M, [**L1**·$NiCl_2$] = 0.028M.

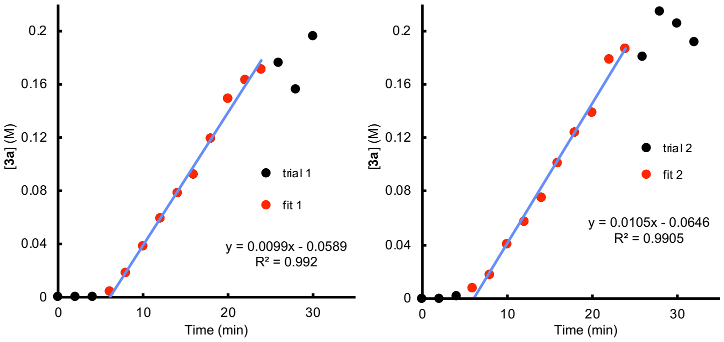

Figure S6: Kinetics runs with linear fit regions highlighted: [**1a**]$_0$ = 0.2M, [**2a**]$_0$ = 0.2M, [**L1**·$NiCl_2$] = 0.04M.

Effect of Changing Alkenyl Bromide 1a Equivalents

The general procedure 1 was followed except varying the amounts of **1a** were used to give final amounts of 1.5, 2, 3, and 4 equivalents.

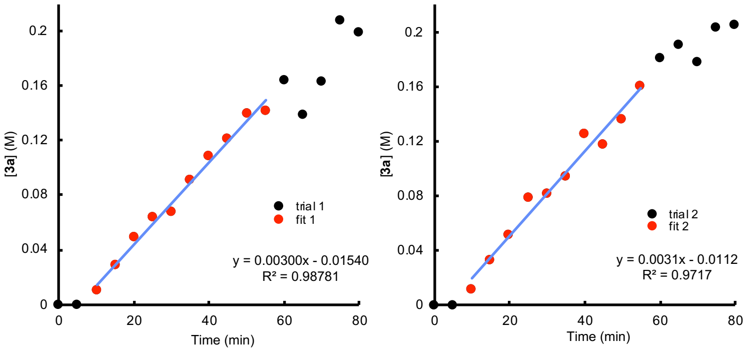

Figure S7: Kinetics runs with linear fit regions highlighted: [**1a**]$_0$ = 0.3M, [**2a**]$_0$ = 0.2M, [**L1**·$NiCl_2$] = 0.02M

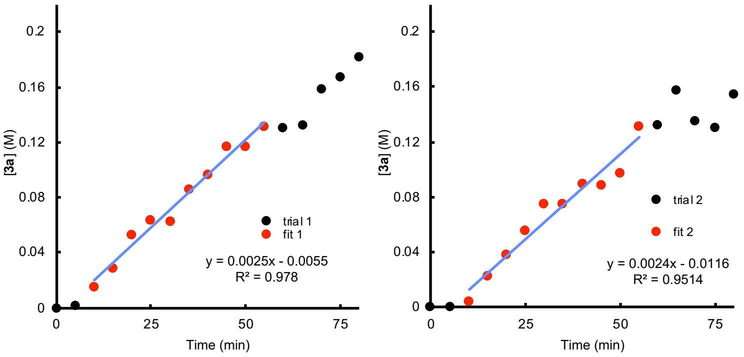

Figure S8: Kinetics runs with linear fit regions highlighted: [**1a**]$_0$ = 0.4M, [**2a**]$_0$ = 0.2M, [**L1**·$NiCl_2$] = 0.02M.

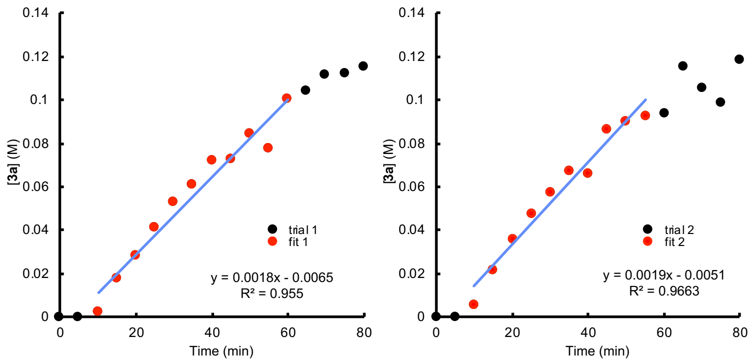

Figure S9: Kinetics runs with linear fit regions highlighted: [**1a**]$_0$ = 0.6M, [**2a**]$_0$ = 0.2M, [**L1**·$NiCl_2$] = 0.02M.

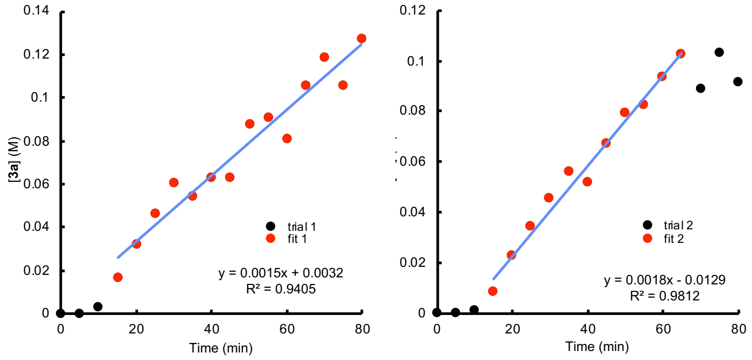

Figure S10: Kinetics runs with linear fit regions highlighted: [**1a**]$_0$ = 0.8M, [**2a**]$_0$ = 0.2M, [**L1**·$NiCl_2$] = 0.02M.

Effect of Changing Benzyl Chloride 2a Equivalents

The general procedure 1 was followed except varying the amounts of **2a** were used to give final amounts of 1.5, 2, 3, and 4 equivalents.

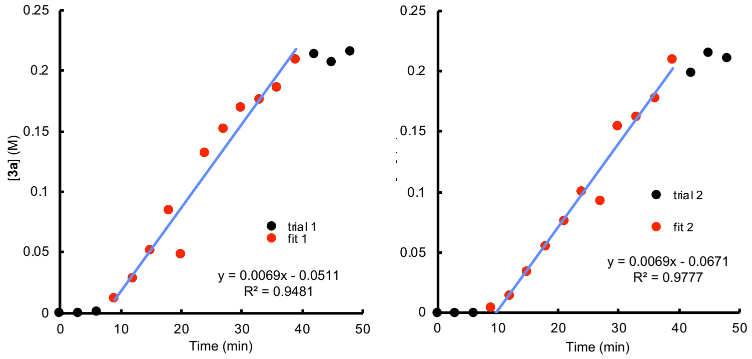

Figure S11: Kinetics runs with linear fit regions highlighted: [**1a**]$_0$ = 0.2M, [**2a**]$_0$ = 0.3M, [**L1**·$NiCl_2$] = 0.02M.

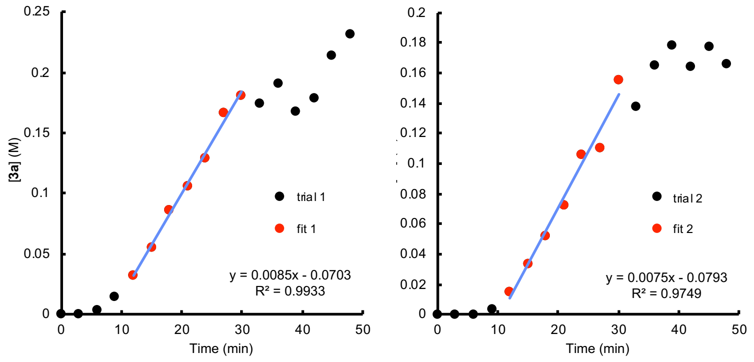

Figure S12: Kinetics runs with linear fit regions highlighted: [**1a**]$_0$ = 0.2M, [**2a**]$_0$ = 0.4M, [**L1**·$NiCl_2$] = 0.02M.

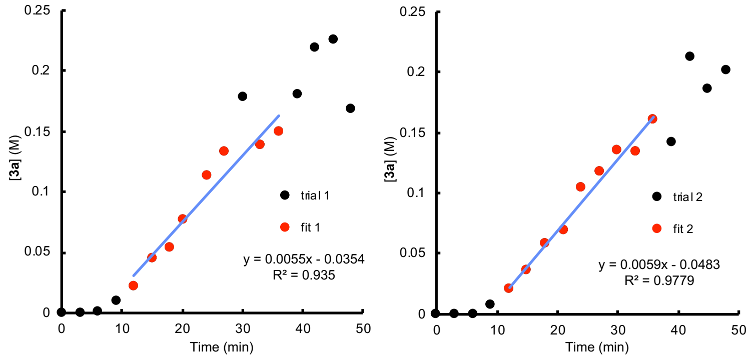

Figure S13: Kinetics runs with linear fit regions highlighted: [**1a**]$_0$ = 0.2M, [**2a**]$_0$ = 0.6M, [**L1**·$NiCl_2$] = 0.02M.

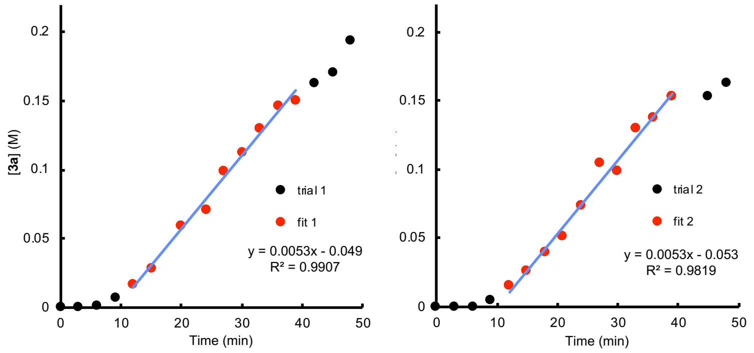

Figure S14: Kinetics runs with linear fit regions highlighted: [**1a**]$_0$ = 0.2M, [**2a**]$_0$ = 0.8M, [**L1**·$NiCl_2$] = 0.02M.

Tabulated Summary of Heterogeneous Kinetics Data:

$[Ni]_0$ (M)	Trial 1 Rate (M/min)	Trial 2 Rate (M/min)	Average Rate (M/min)	Standard deviation	**3a** ee at End of Trial 1	**3a** ee at End of Trial 2
0.02	0.0050	0.0051	0.0050	0.0001	93	93
0.01	0.0025	0.0021	0.0023	0.0003	87	88
0.014	0.0035	0.0037	0.0036	0.0001	89	89
0.028	0.0061	0.0059	0.0060	0.0002	86	85
0.04	0.0099	0.011	0.010	0.0004	85	85

Table S1: Reaction rate data for each trial at different Ni loadings.

[**1a**]$_0$ (M)	Trial 1 Rate (M/min)	Trial 2 Rate (M/min)	Average Rate (M/min)	Standard deviation	**3a** ee at End of Trial 1	**3a** ee at End of Trial 2
0.2	0.0050	0.0051	0.0050	0.0001	93	93
0.3	0.0030	0.0031	0.0030	0.0001	93	91
0.4	0.0025	0.0025	0.0025	0.0001	94	94
0.6	0.0016	0.0017	0.0017	0.00003	95	95
0.8	0.0018	0.0015	0.017	0.0002	95	95

Table S2: Reaction rate data for each trial at different initial concentrations of **1a**.

[**2a**]$_0$ (M)	Trial 1 Rate (M/min)	Trial 2 Rate (M/min)	Average Rate (M/min)	Standard deviation	**3a** ee at End of Trial 1	**3a** ee at End of Trial 2
0.2	0.0050	0.0051	0.0050	0.0001	93	93
0.3	0.0069	0.0067	0.0068	0.0001	83	84
0.4	0.0075	0.0085	0.0080	0.0007	84	83
0.6	0.0055	0.0059	0.0057	0.0003	82	83
0.8	0.0052	0.0053	0.0053	0.0001	83	83

Table S3: Reaction rate data for each trial at different initial concentrations of **2a**.

Impact of Stir Rate

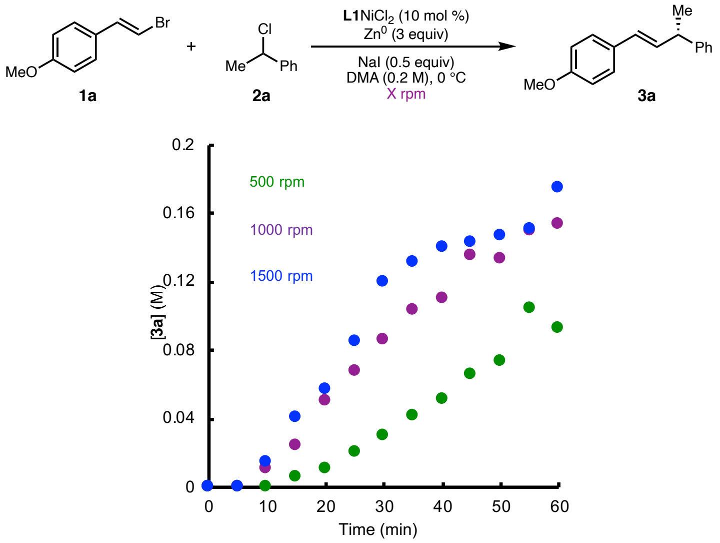

Figure S15: Rate of **3a** formation at different stir rates (average of 2 runs shown). There is a a significant rate dependence on stirring from 500 rpm to 1000 rpm and a smaller difference between 1000 rpm and 1500 rpm. The stir rate dependence is smaller at high stir rates and kinetic runs measured runs at 1500 rpm are reproducible.

Mn^0 vs. Zn^0 Profile:

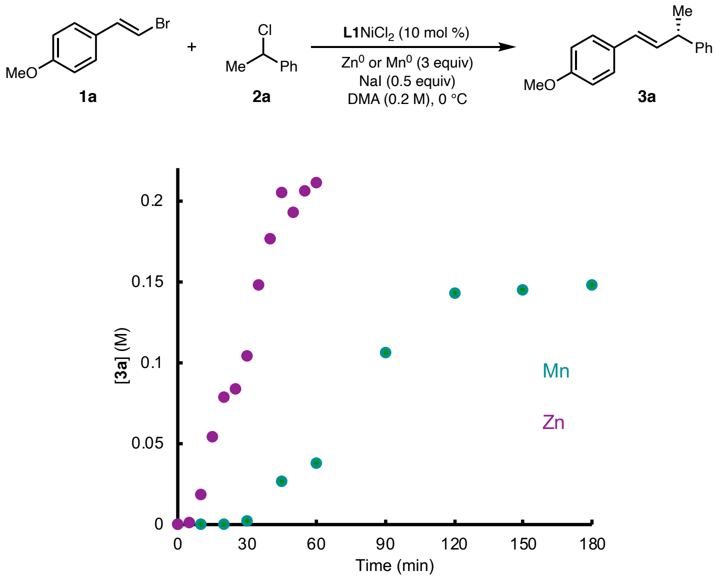

Figure S16: Representative profile of **3a** formation with Zn^0 and Mn^0 stirring at 1500 rpm.

Excess Zn^0 Profile

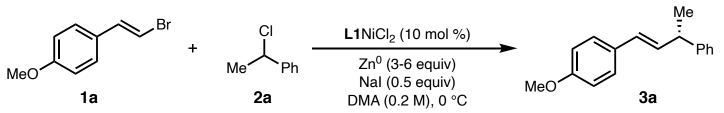

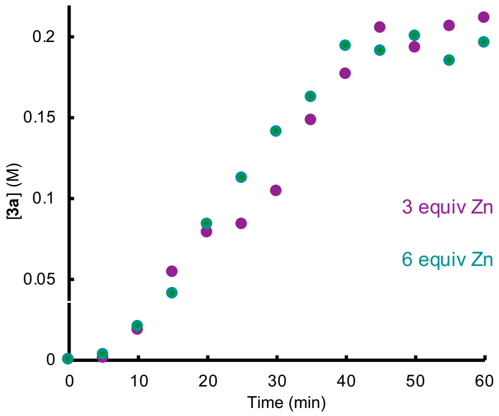

Zn equiv	Rate (M/min)
3	0.0050
6	0.0053

Figure S17: Rate of **3a** formation with 3 equiv and 6 equiv of Zn^0 stirring at 1500 rpm shown as average of two runs for each loading.

3.2 Homogenous Reaction Kinetics

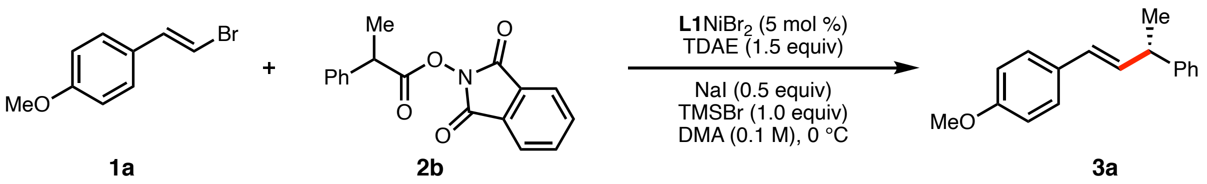

General Procedure 2: To a 2mL volumetric flask was added (*E*)-1-(2-bromovinyl)-4-methoxybenzene (65.6 mg, 0.308 mmol, 1.0 equiv), 1,3-dioxoisoindolin-2-yl 2-phenylpropanoate (90.9 mg, 0.308 mmol, 1.0 equiv) and **L1**·$NiBr_2$ (8.8 mg, 0.0154 mmol, 5 mol%) on the bench. The volumetric flask was then placed under argon and sealed with a septa. In a N_2-filled glovebox was then added sodium iodide (23.1 mg, 0.154 mmol, 0.5 equiv) to the volumetric flask which was then filled to volume with DMA (0.154 M). A stir bar was then added and the solution was stirred for 15 minutes. In a 1mL volumetric flask was added *N,N'*-tetrakis(dimethylamino)ethylene (TDAE, 100μL, 0.43 mmol, 1.5 equiv) and then filled to volume with DMA (0.43 M) in the glovebox and sealed with a rubber septum and tape. Then 1.3mL of the homogeneous solution in the 2 mL volumetric flask was then added to a 10mL oven-dried round bottom flask with a 2 dram stir bar. The flask was then sealed with a rubber

septum and electrical tape. To the 10mL round bottom flask was added 15µL of dodecane as the internal standard followed by TMSBr (26.4 µL, 0.2 mmol, 1.0 equiv). The round bottom flask was then quickly removed from the glovebox and placed under N_2 with an inlet needle attached to a Schenck line and placed in an ice bath and stirred. A ~50 µL aliquot were removed by an N_2-purged 1mL syringe for a t = 0 timepoint. Once cooled, TMSBr (26.4 µL, 0.2 mmol, 1.0 equiv) was added via syringe and the reaction was stirred for 30 seconds before 0.7 mL of the cooled TDAE solution was added initiating the reaction. ~50 µL aliquots were removed by an N_2-purged 1mL syringe and quenched into a 1-dram vial containing EtOAc and 1M $HCl_{(aq)}$. The vial was then capped and shaken then the organic layer was removed and pushed through a $MgSO_4$ plug into a GC vial for analysis. All samples were analyzed directly by GC-FID. Each experiment was run in duplicate with representative profiles shown below.

Standard Reaction Conditions

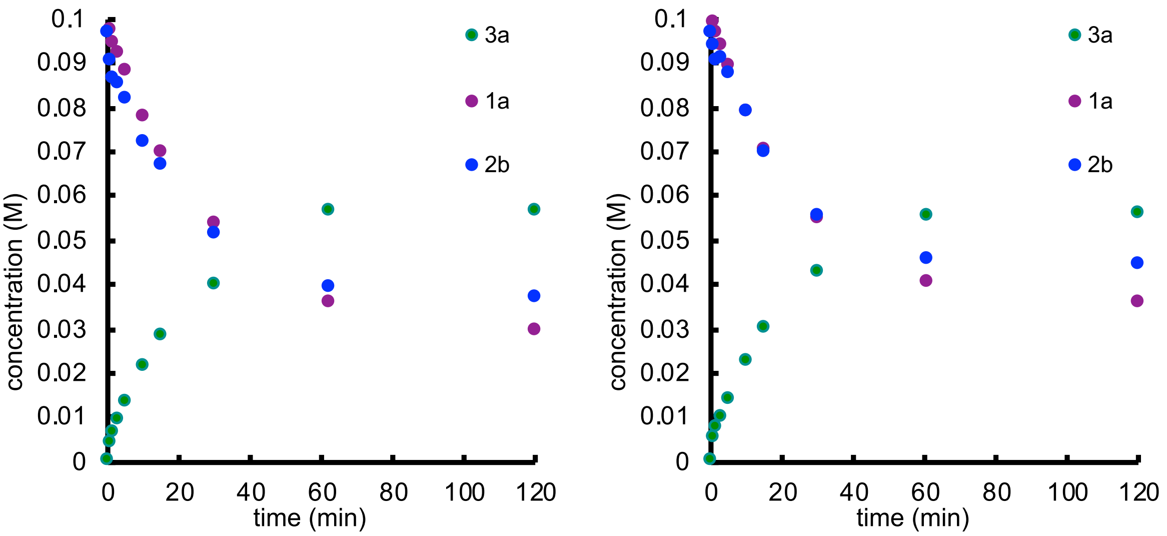

Figure S18: Results of standard reaction conditions under general procedure 2: [**1a**]$_0$ = 0.1M, [**2b**]$_0$ = 0.1M, [**L1**·$NiBr_2$] = 0.005 M.

Reaction Profiles for Experiments Varying [L1NiBr$_2$]$_0$

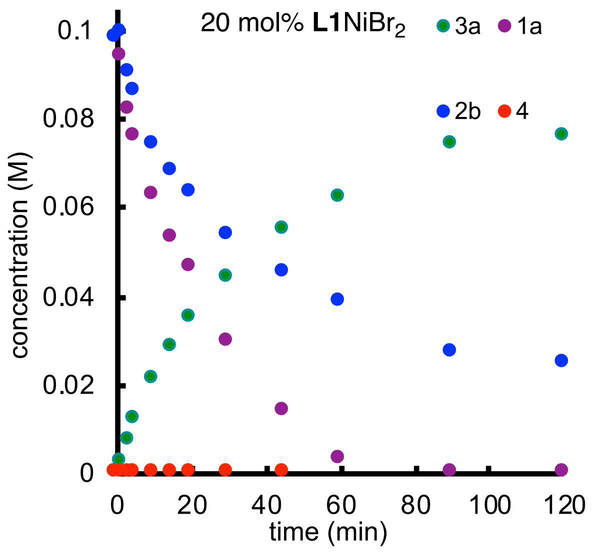

Figure S19: Profile of reaction under general procedure 2: [**1a**]$_0$ = 0.1M, [**2b**]$_0$ = 0.1M, [**L1**·NiBr$_2$] = 0.02 M.

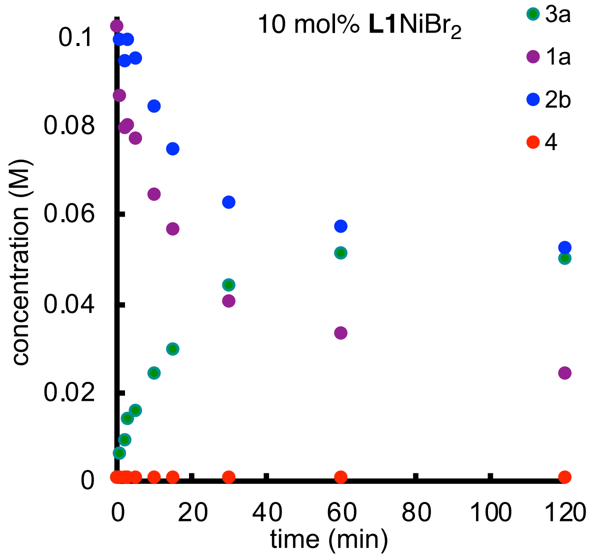

Figure S20: Profile of reaction under general procedure 2: [**1a**]$_0$ = 0.1M, [**2b**]$_0$ = 0.1M, [**L1**·NiBr$_2$] = 0.01 M.

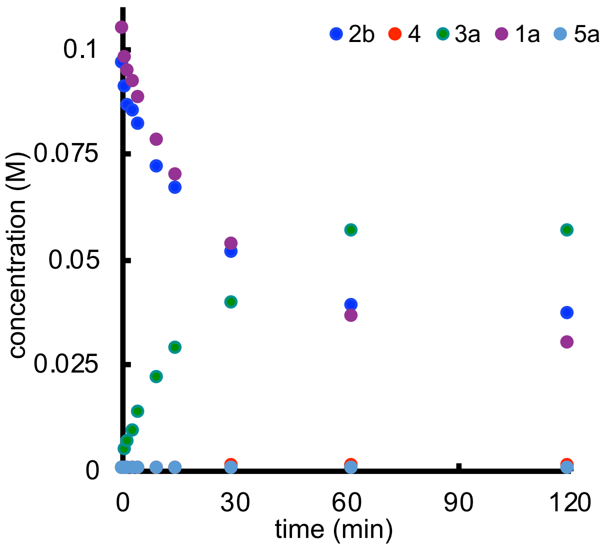

Figure S21: Profile of reaction under general procedure 2: [**1a**]$_0$ = 0.1M, [**2b**]$_0$ = 0.1M, [**L1**·NiBr$_2$] = 0.005 M.

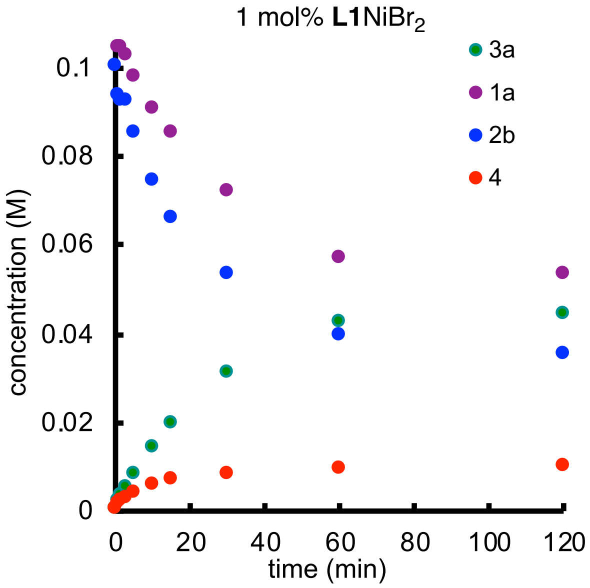

Figure S22: Profile of reaction under general procedure 2: [**1a**]$_0$ = 0.1M, [**2b**]$_0$ = 0.1M, [**L1**·NiBr$_2$] = 0.001 M.

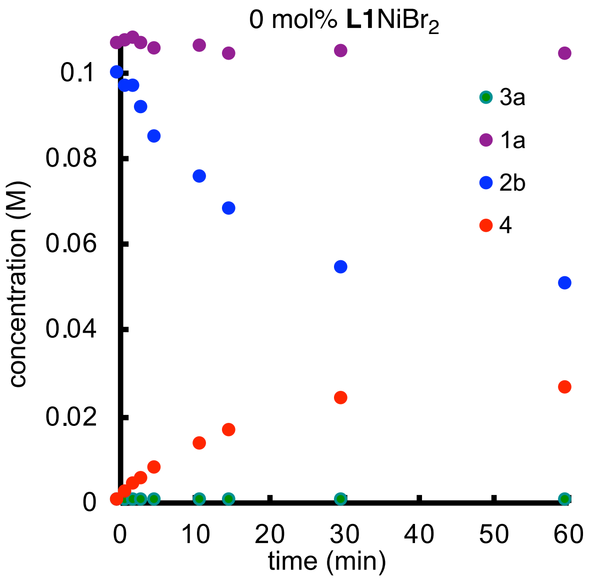

Figure S23: Profile of reaction under general procedure 2: [**1a**]$_0$ = 0.1M, [**2b**]$_0$ = 0.1M, [**L1**·NiBr$_2$] = 0 M.

Reaction Profiles for Experiments Varying [1a]$_0$

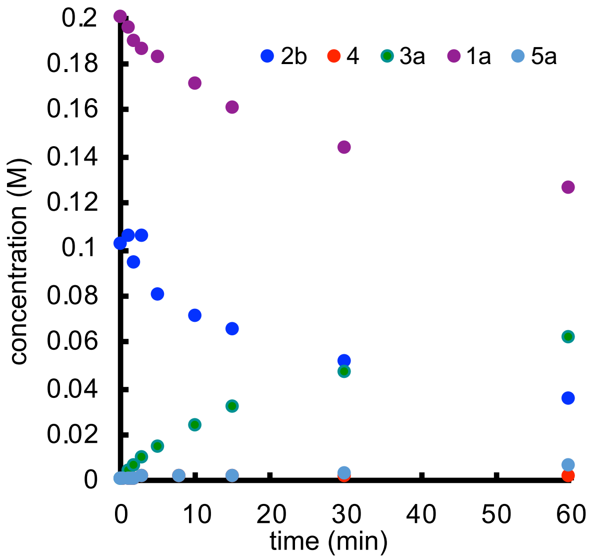

Figure S24: Profile of reaction under general procedure 2: [**1a**]$_0$ = 0.2M, [**2b**]$_0$ = 0.1M, [**L1**·NiBr$_2$] = 0.005 M. This example shows significant amounts of **5a** (6% at end of reaction monitoring) so it is explicitly included in the reaction profile data,

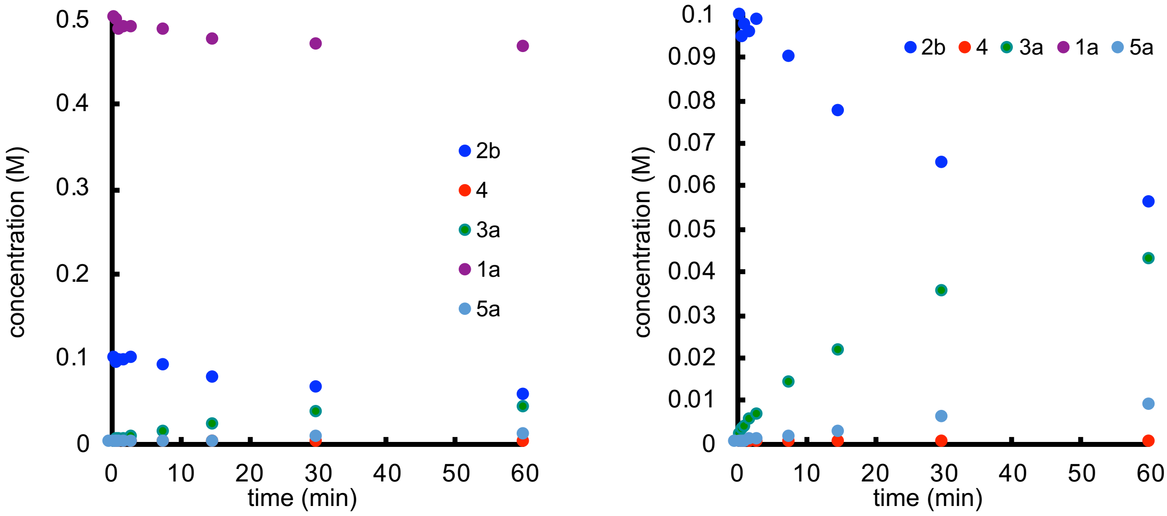

Figure S25: Profile of reaction under general procedure 2: [**1a**]$_0$ = 0.5M, [**2b**]$_0$ = 0.1M, [**L1**·NiBr$_2$] = 0.005 M (left) and inset (right) showing formation of **5a** in significant quantities (9% at 60 min) under these conditions.

Reaction Profiles for Experiments Varying [2b]$_0$

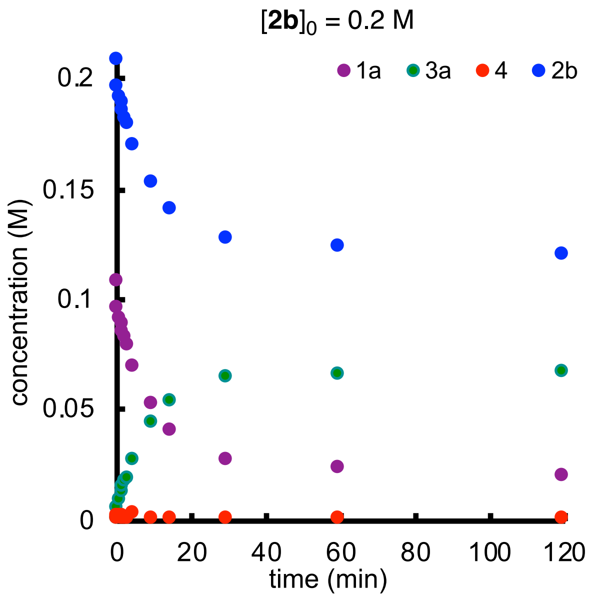

Figure S26: Profile of reaction under general procedure 2: [**1a**]$_0$ = 0.1M, [**2b**]$_0$ = 0.2M, [**L1**·NiBr$_2$] = 0.005 M.

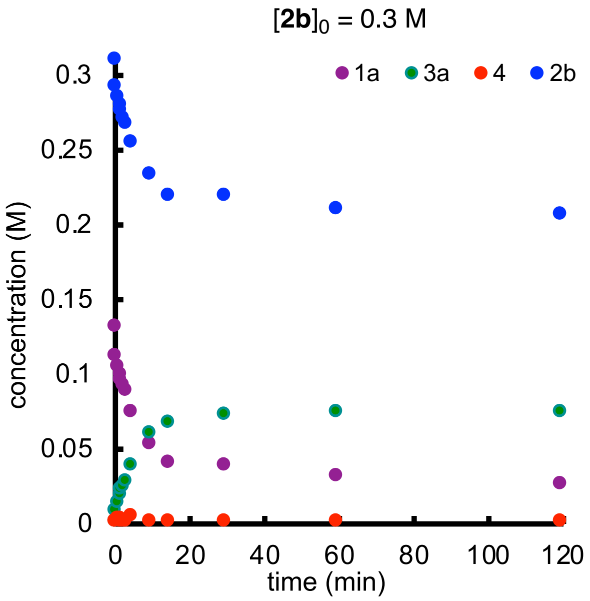

Figure S26: Profile of reaction under general procedure 2: [**1a**]$_0$ = 0.1M, [**2b**]$_0$ = 0.3M, [**L1**·$NiBr_2$] = 0.005 M.

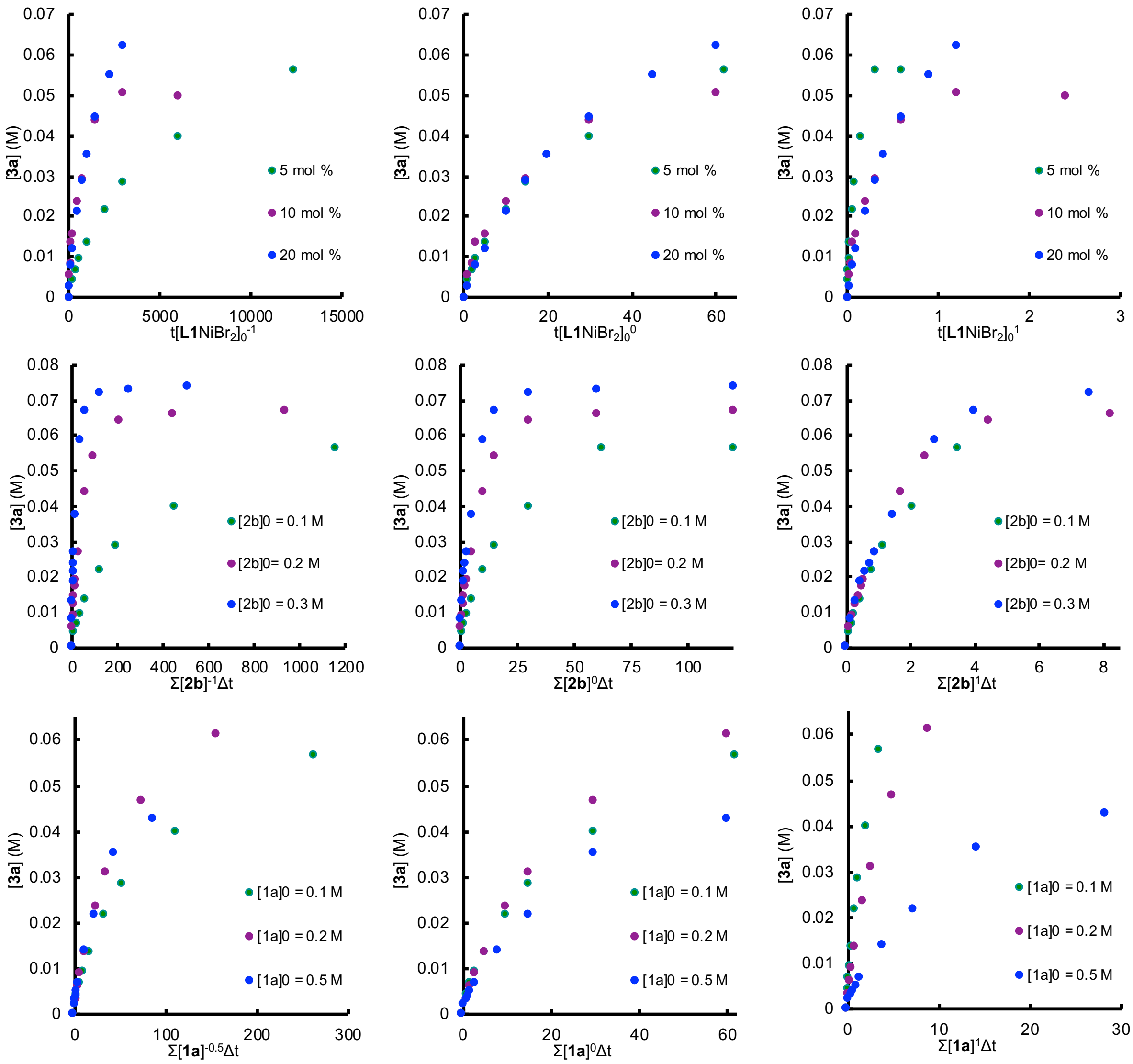

Figure S27: Grid of reactions analyzed by Variable Time Normalization Analysis (VTNA) showing the coefficients that result in the best profile overlay next to coefficients that show poor overlay.

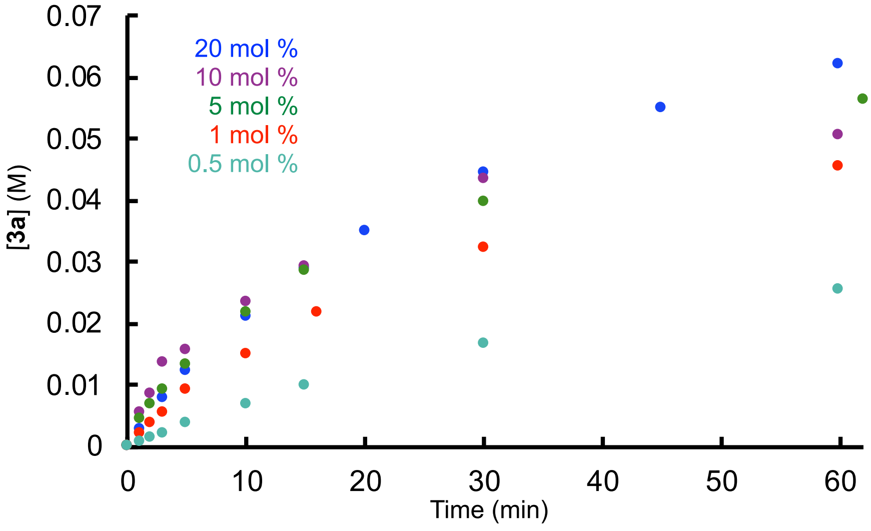

Figure S28: Comparison of the rate of product **3a** formation at different **L1**·$NiBr_2$ catalyst loadings. The positive rate dependence on catalyst can be seen in the 1 mol % and 0.5 mol % runs which are slower than the 20-5 mol %.

Order in TDAE

The rate dependence on TDAE was determined according to General Procedure 2 with different loadings of TDAE (1, 1.5, 2, 3, 4.5 equivs). The rate was then obtained from the initial rate of **3a** formation within the first 20% conversion. The data suggests an positive rate dependence on TDAE with saturation to pseudo 0th order $\geq$ 3 equiv.

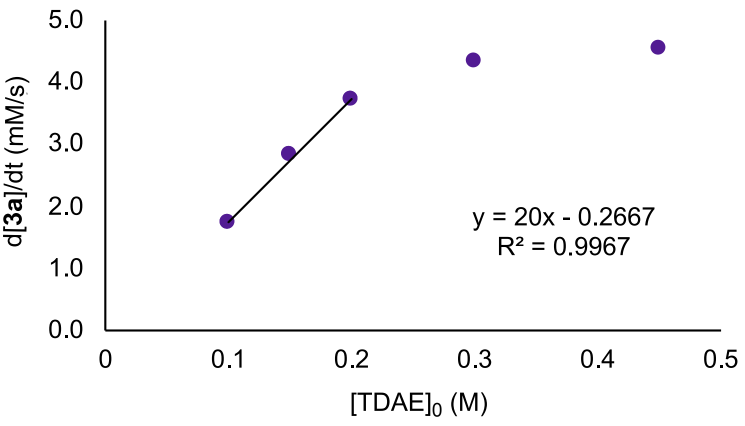

Figure S29: Changes in initial rate of **3a** formation with different $[TDAE]_0$ concentrations. Reactions were run according to general procedure 2 with $[TDAE]_0$ values of 0.1, 0.15, 0.2, 0.3, and 0.45 M.

Order in TMSBr

The rate dependence on TMSBr was determined according to General Procedure 2 with different loadings of TMSBr (1, 2, 4 equivs). The rate was then obtained from the initial rate of **3a** formation within the first 20% conversion. The data suggests an positive rate dependence on TDAE with saturation to pseudo 0th order ≥ 3 equiv.

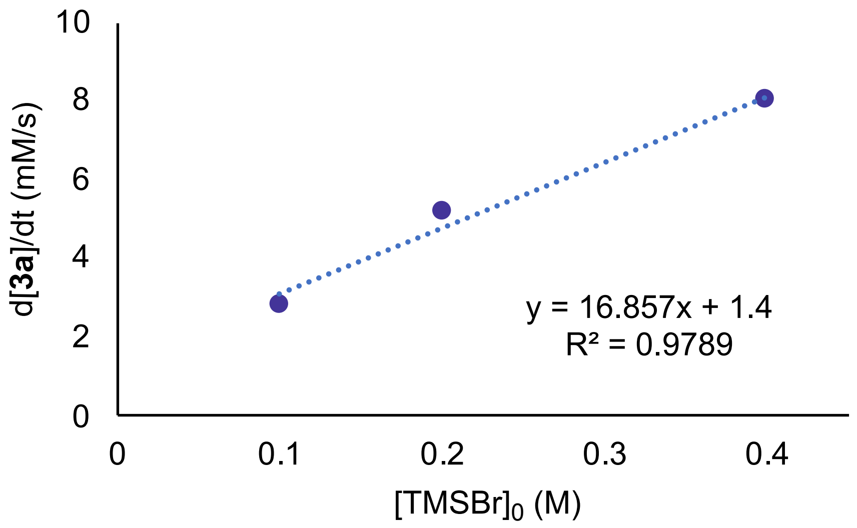

Figure S30: Changes in initial rate of **3a** formation with different $[TMSBr]_0$ concentrations. Reactions were run according to general procedure 2 with $[TMSBr]_0$ values of 0.1, 0.2, and 0.4 M. Across a wide concentration range there maintains a positive rate dependence on TMSBr.

Monitoring the ee of 3a

The ee of product **3a** was monitored over the course of the reaction according to Procedure 2 but on 0.6 mmol scale and 0.2 mL aliquots were removed. Half of the volume of the aliquot was analyzed by GC-FID to determine the concentration of reaction components while the other half was purified by preparative TLC and analyzed by chiral SFC to determine ee.

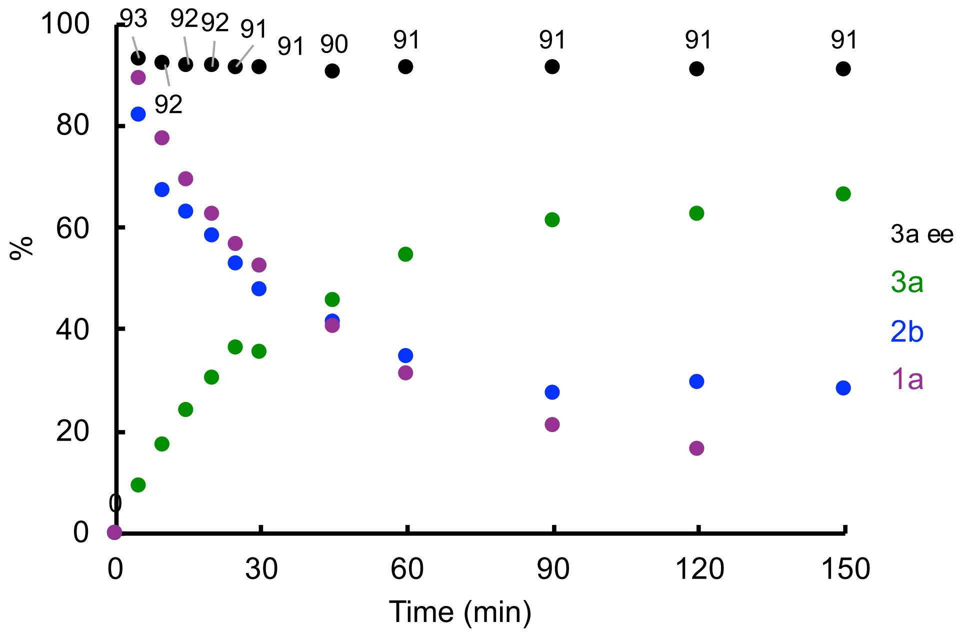

Figure S31: Plot showing the percent conversion of starting materials **1a** and **2b**, and the percent yield and ee of product **3a**.

4. Mechanism of Substrate Activation

4.1 Additive Effects of NHP Ester Reduction Rate

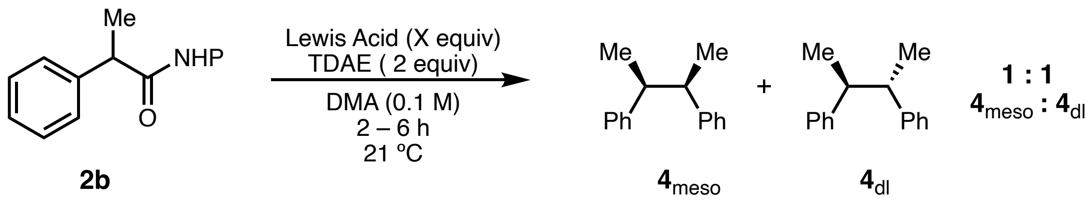

General Procedure 3: To an oven-dried 10 mL round bottom flask with a stir bar was added 1,3-dioxoisoindolin-2-yl 2-phenylpropanoate **2b** (59.1 mg, 0.20 mmol, 1.0 equiv). The flask was then brought into a N_2-filled glovebox where NaI (if applicable), DMA (2.0 mL, 0.1 M) and *n*-dodecane (target: 20 µL, 0.088 mmol, 0.44 eqiuv, actual mass was recorded for each experiment) internal standard was added. The solution was stirred until homogenous and then the respective Lewis acid (0.2 mmol, 1 equiv) was added and the flask was sealed with a septa and electrical tape. The flask was removed from the glovebox and placed under N_2 and stirred. A ~50 µL aliquot of the solution was removed with a N_2-purged syringe then quenched into a 1-dram vial containing EtOAc and 1M $HCl_{(aq)}$ and the organic layer was passed through a $MgSO_4$ plug into a GC vial then further diluted with EtOAc for the appropriate concentration

for GC analysis. To the stirring solution was then added *N,N'*-tetrakis(dimethylamino)ethylene (93.1 μL, 0.40 mmol, 2.0 equiv) to start the reaction (t = 0). The reaction was then aliquoted with the same procedure previously described at regular intervals.

Data Analysis: The calculated concentrations of **2b**, meso-**4** and dl-**4** were calculated from the analyte:standard integral ratios measured by GC-FID at each timepoint. The measured response for each component was used to calculate the amount of analyte which was then converted to concentration corresponding to 2 mL reaction volume. In all cases, diastereomers of **4** were produced in a 1:1 ratio and summed to determine the total amount of product produced ($[\mathbf{4}]_{meso} + [\mathbf{4}]_{dl} = [\mathbf{4}]_{tot}$, noted as [**4**] throughout the remainder of the text). Due to the fact that the formation of **4** from the diffusion-limited termination of two **2b**-derived radicals is much faster than the reactions leading to the generation of the radical species it is reasonable to treat the rate of radical generation as the rate of [**4**] formation.

The relative rate of **2b**-derived radical formation was determined from the [**4**] vs. time profiles for each Lewis acid additive. The rates were determined in a similar method as described by Weix and coworkers[7] using equation **1** to linearize the data.

$$\frac{1}{(1-f)} = mt \qquad (1)$$

Where f is the fraction of $[\mathbf{4}]_t$ over the theoretical yield of [**4**]. The rate was then extracted from the slope determined from least-squares linear regression. To calculate the relative rate (k_{rel}) the absolute rate obtained from experiments employing 1 equivalent of TMSBr was used as a baseline according to equation **2** due to its use in the optimized catalytic reaction.

$$k_{rel} = \frac{k_{Lewis\ acid}}{k_{TMSBr}} \qquad (2)$$

Representative Concentration Profiles and Rate Determination:

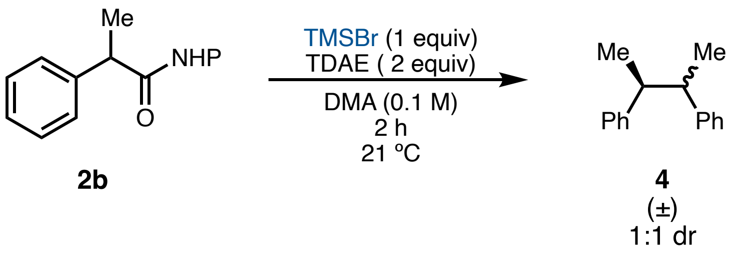

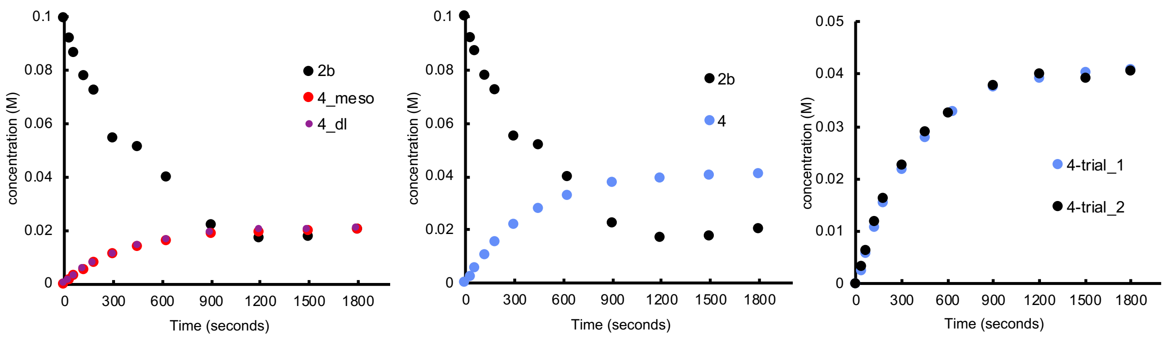

Figure S32: Representative reaction profile for **2b** activation kinetics. The profile is shown with all quantified species (left) and with the sum of **4** diastereomers (middle). Runs were run in duplicate to ensure reproducibility (right).

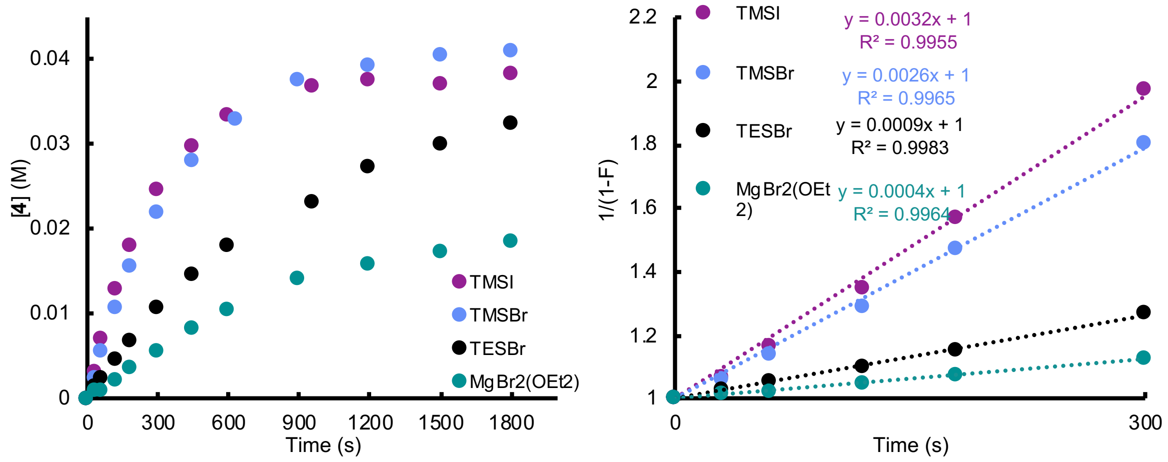

Figure S33: Comparative reaction profiles for different Lewis acids (left) and the corresponding linearized data with equation **1** (right).

Tabulated Rate Data:

Lewis Acid	TMSBr	1 equiv
k	0.002625	
krel	1.000	
Time (s)	[4] (M)	1/(1-F)
0	0.000	1.00
30	0.002	1.05
60	0.006	1.13
120	0.011	1.27
180	0.016	1.45
300	0.022	1.78
450	0.028	2.27
630	0.033	2.91
900	0.038	4.04
1200	0.039	4.70
1500	0.040	5.20
1800	0.041	5.59
2700	0.041	5.62
3600	0.041	5.88
5400	0.039	4.61
7202	0.043	6.88

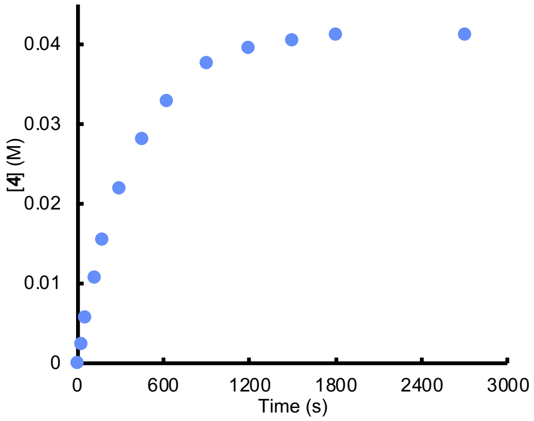

Table S4: Reaction data and linearization by general procedure 3. $[TMSBr]_0 = 0.1$ M.

Lewis Acid	TMSI	1 equiv
k	0.00317	
krel	1.210	
Time (s)	[4] (M)	1/(1-F)
0	0.000	1.00
30	0.003	1.07
60	0.007	1.17
120	0.013	1.35
180	0.018	1.57
300	0.025	1.97
450	0.030	2.48
600	0.033	3.02
960	0.037	3.83
1200	0.038	4.03
1500	0.037	3.85
1800	0.038	4.25
2700	0.038	4.27
3600	0.038	4.15
5400	0.039	4.42

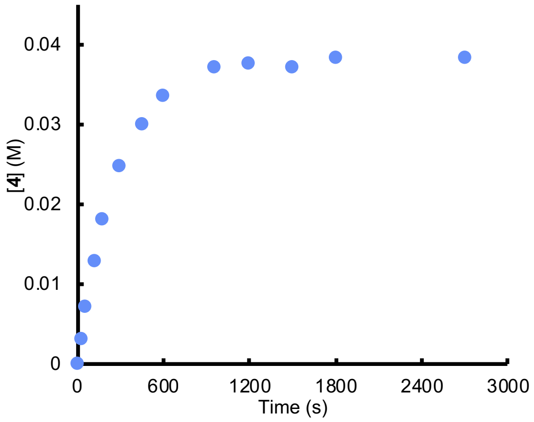

Table S5: Reaction data and linearization by general procedure 3. $[TMSI]_0 = 0.1$ M.

Lewis Acid	TMSOTf	1 equiv
k	0.00296	
krel	1.128	
Time (s)	[4] (M)	1/(1-F)
0	0.000	1.00
30	0.003	1.07
60	0.007	1.16
120	0.013	1.36
180	0.018	1.55
300	0.023	1.88
450	0.028	2.24
600	0.030	2.52
960	0.033	2.91
1200	0.033	2.97
1500	0.034	3.08
1800	0.034	3.16
2700	0.035	3.24
3600	0.034	3.22
5400	0.034	3.19

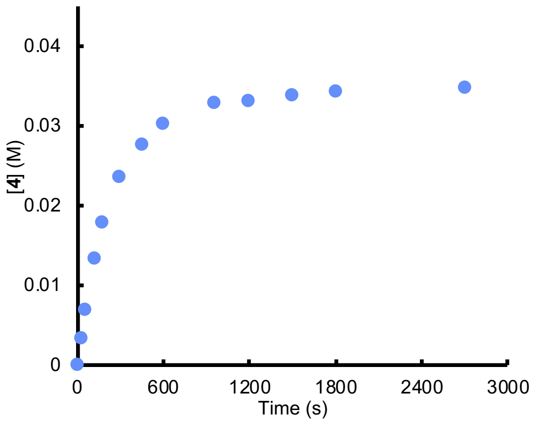

Table S5: Reaction data and linearization by general procedure 3. $[TMSOTf]_0 = 0.1$ M.

Lewis Acid	TESBr	1 equiv
k	0.00088	
krel	0.335	
Time (s)	[4] (M)	1/(1-F)
0	0.000	1.00
30	0.002	1.03
60	0.002	1.05
120	0.005	1.10
180	0.007	1.15
300	0.011	1.27
450	0.015	1.41
600	0.018	1.56
960	0.023	1.87
1200	0.027	2.21
1500	0.030	2.51
1800	0.032	2.84
2700	0.036	3.53
3600	0.037	3.85
5400	0.038	4.05

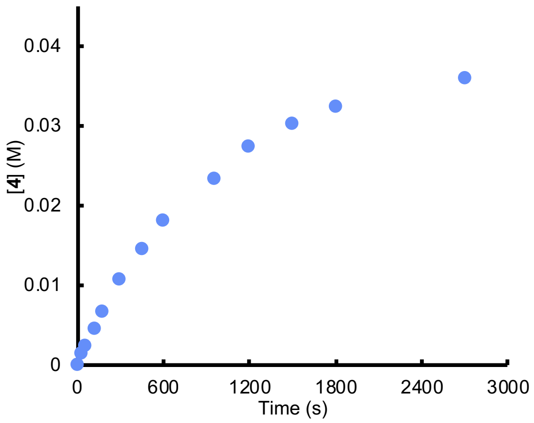

Table S6: Reaction data and linearization by general procedure 3. $[TESBr]_0 = 0.1$ M.

Lewis Acid	TMSBr	2 equiv
k	0.0044	
krel	1.676	
Time (s)	[4] (M)	1/(1-F)
0	0.000	1.00
30	0.005	1.11
60	0.010	1.24
120	0.016	1.47
180	0.021	1.75
300	0.029	2.38
450	0.035	3.39
600	0.039	4.73
900	0.045	10.23
1200	0.048	32.81
1500	0.051	-70.94

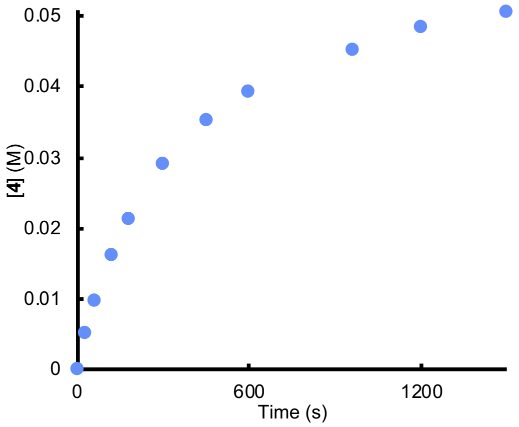

Table S7: Reaction data and linearization by general procedure 3. $[TMSBr]_0 = 0.2$ M.

Lewis Acid	TMSCl	1 equiv
k	0.00024	
krel	0.091	
Time (s)	[4] (M)	1/(1-F)
0	0.000	1.00
30	0.001	1.02
60	0.001	1.02
120	0.002	1.04
180	0.002	1.05
300	0.004	1.08
450	0.005	1.12
600	0.007	1.15
900	0.010	1.24
1200	0.013	1.34
1500	0.015	1.44
1800	0.017	1.53
2700	0.023	1.84
3600	0.026	2.06
5400	0.031	2.59

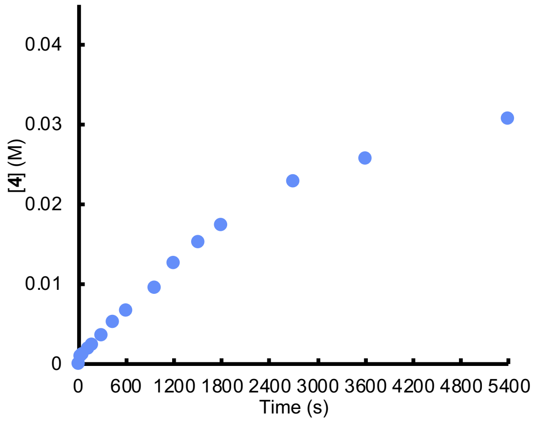

Table S8: Reaction data and linearization by general procedure 3. $[TMSCl]_0 = 0.1$ M.

Lewis Acid	TESCl	1 equiv
k	0.000031	
krel	0.012	
Time (s)	[4] (M)	1/(1-F)
0	0.000	1.00
30	0.000	1.00
60	0.000	1.00
120	0.000	1.01
180	0.000	1.01
300	0.000	1.01
450	0.001	1.01
600	0.001	1.02
960	0.001	1.02
1200	0.001	1.03
1500	0.001	1.03
1800	0.002	1.03
2700	0.002	1.05
3600	0.003	1.07
5400	0.005	1.12

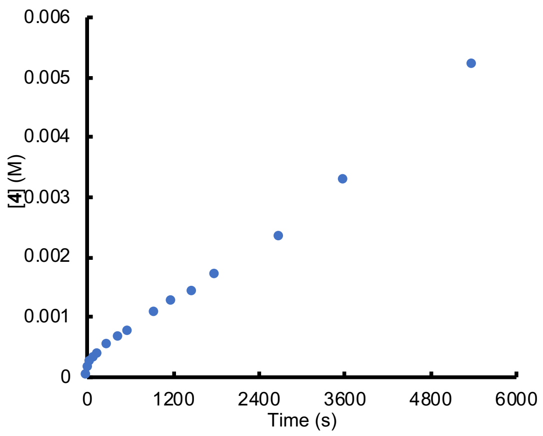

Table S9: Reaction data and linearization by general procedure 3. $[TESCl]_0 = 0.1$ M.

Lewis Acid	TBSCl	1 equiv
k	0.0000049	
krel	0.002	
Time (s)	[4] (M)	1/(1-F)
0	0.000	1.00
300	0.000	1.00
600	0.000	1.00
900	0.000	1.00
1800	0.000	1.01
2700	0.000	1.01
3600	0.001	1.01
5400	0.001	1.02
7200	0.001	1.03
9000	0.002	1.03
10860	0.002	1.05
12600	0.002	1.05
14400	0.003	1.07
18000	0.004	1.10

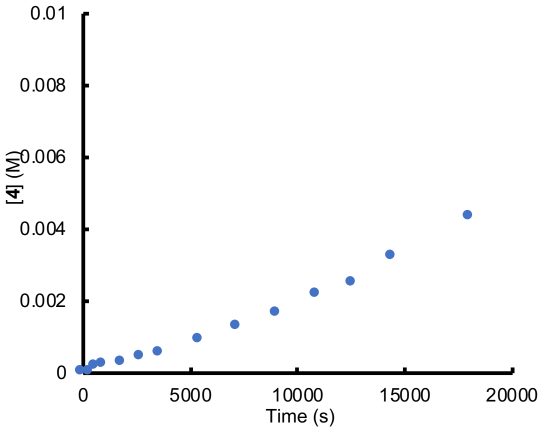

Table S10: Reaction data and linearization by general procedure 3. $[TBSCl]_0 = 0.1$ M.

Lewis Acid	MnCl2	1 equiv
k	0.000094	
krel	0.036	
Time (s)	[4] (M)	1/(1-F)
0	0.000	1.00
30	0.000	1.01
60	0.001	1.01
120	0.001	1.02
180	0.001	1.02
300	0.002	1.03
450	0.002	1.05
600	0.003	1.07
900	0.004	1.10
1200	0.006	1.13
1500	0.007	1.15
1800	0.007	1.18
2700	0.010	1.26
3600	0.013	1.35
5400	0.016	1.49

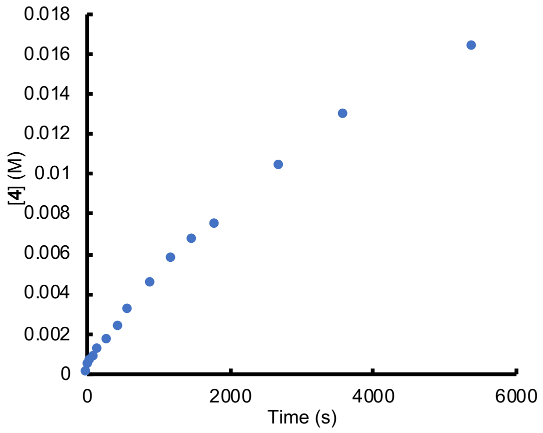

Table S11: Reaction data and linearization by general procedure 3. $[MnCl_2]_0 = 0.1$ M.

Lewis Acid	MgBr2(OEt2)	1 equiv
k	0.00044	
krel	0.147	
Time (s)	[4] (M)	1/(1-F)
0	0.000	1.00
30	0.001	1.02
60	0.001	1.02
120	0.002	1.05
180	0.004	1.08
300	0.006	1.13
450	0.008	1.20
600	0.011	1.27
900	0.014	1.39
1200	0.016	1.47
1500	0.017	1.53
1800	0.019	1.59
2700	0.019	1.59
3600	0.019	1.64
6300	0.020	1.67
9000	0.021	1.70

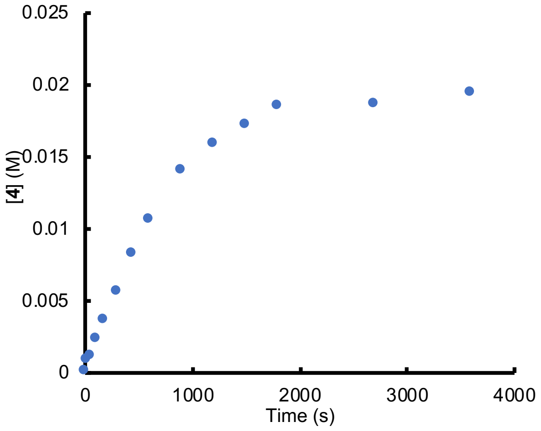

Table S12: Reaction data and linearization by general procedure 3. $[MgBr_2{\cdot}OEt_2]_0 = 0.1$ M.

Lewis Acid	Sc(OTf)3	1 equiv
k	0.00038	
krel	0.127	
Time (s)	[4] (M)	1/(1-F)
0	0.000	1.00
30	0.001	1.02
60	0.002	1.03
120	0.003	1.06
180	0.004	1.08
300	0.006	1.14
450	0.008	1.20
600	0.010	1.25
900	0.013	1.36
1200	0.016	1.47
1500	0.018	1.57
1800	0.021	1.72
2700	0.026	2.07
3600	0.030	2.51
5400	0.036	3.70

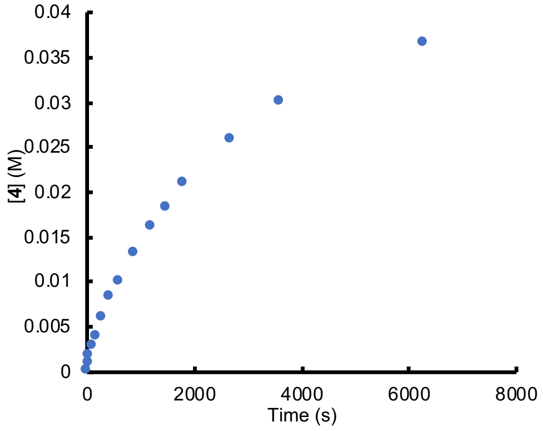

Table S13: Reaction data and linearization by general procedure 3. $[Sc(OTf)_3]_0$ = 0.1 M.

Lewis Acid	LiBr	1 equiv
k	0.000043	
krel	0.014	
Time (s)	[4] (M)	1/(1-F)
0	0.000	1.00
30	0.000	1.01
60	0.000	1.01
120	0.000	1.01
180	0.000	1.01
300	0.001	1.01
450	0.001	1.02
600	0.001	1.02
900	0.002	1.04
1200	0.002	1.05
1500	0.003	1.06
1800	0.003	1.07
2700	0.005	1.12
3600	0.007	1.15
5400	0.010	1.26
7200	0.013	1.35

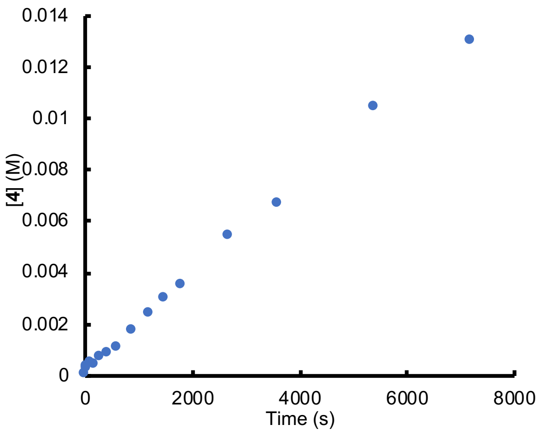

Table S14: Reaction data and linearization by general procedure 3. $[LiBr]_0$ = 0.1 M.

Lewis Acid	ZnI2	1 equiv
k	0.000037	
krel	0.012	
Time (s)	[4] (M)	1/(1-F)
0	0.000	1.00
30	0.001	1.01
60	0.001	1.02
120	0.001	1.02
180	0.002	1.03
300	0.002	1.04
450	0.002	1.05
600	0.003	1.06
900	0.003	1.07
1200	0.004	1.08
1500	0.004	1.09
1800	0.004	1.10
2700	0.005	1.12
3600	0.006	1.14
5400	0.007	1.17
7200	0.008	1.19

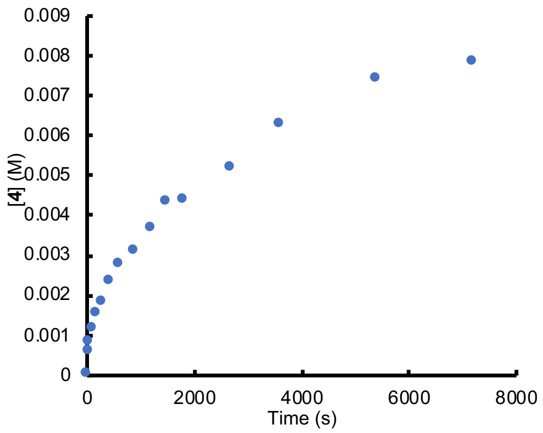

Table S15: Reaction data and linearization by general procedure 3. $[ZnI_2]_0 = 0.1$ M.

Lewis Acid	TMSBr, NaI	1 equiv, 0.5 equiv
k	0.0046	
krel	1.533	
Time (s)	[4] (M)	1/(1-F)
0	0.000	1.00
30	0.004	1.09
60	0.008	1.20
120	0.015	1.43
180	0.020	1.69
300	0.028	2.24
450	0.033	2.96
600	0.037	3.79
900	0.038	4.01
1200	0.039	4.68
1500	0.040	5.11
1800	0.040	4.98
2700	0.041	5.60
3600	0.041	5.76
5400	0.042	6.07

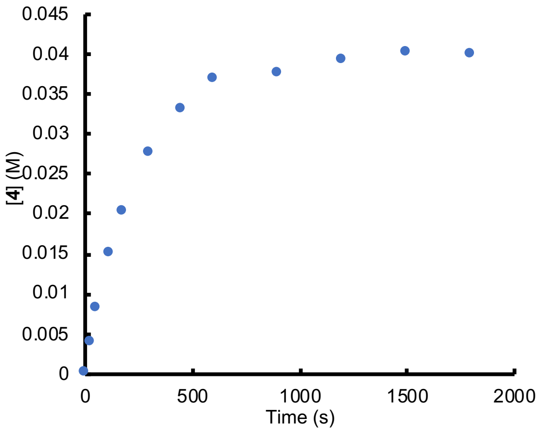

Table S16: Reaction data and linearization by general procedure 3. $[TMSBr]_0 = 0.1$ M, $[NaI]_0 = 0.05$ M.

Lewis Acid	TMSCl, NaI	1 equiv, 1 equiv
k	0.000549	
krel	0.209	
Time (s)	[4] (M)	1/(1-F)
0	0.000	1.00
30	0.001	1.02
60	0.002	1.04
120	0.003	1.07
180	0.004	1.10
300	0.007	1.16
450	0.009	1.23
600	0.012	1.31
960	0.015	1.43
1200	0.018	1.58
1500	0.021	1.72
1800	0.022	1.76
2700	0.026	2.07
3600	0.028	2.28
5400	0.031	2.65
7200	0.034	3.10

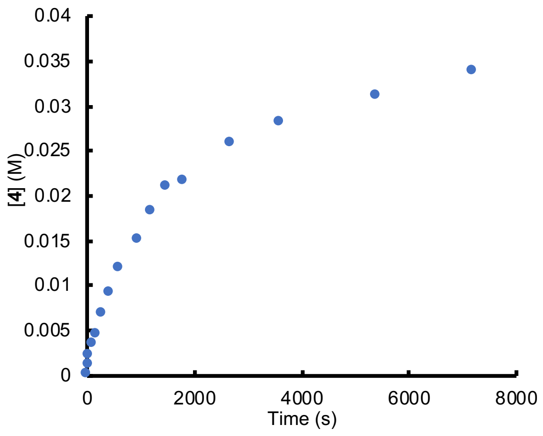

Table S17: Reaction data and linearization by general procedure 3. $[TMSCl]_0 = 0.1$ M, $[NaI]_0 = 0.1$ M.

Lewis Acid	TESCl, NaI	1 equiv, 1 equiv
k	0.000068	
krel	0.026	
Time (s)	[4] (M)	1/(1-F)
0	0.000	1.00
30	0.000	1.00
60	0.000	1.01
120	0.001	1.01
180	0.001	1.02
300	0.001	1.02
450	0.002	1.03
600	0.002	1.04
1200	0.003	1.06
1500	0.003	1.06
1800	0.003	1.07
2700	0.004	1.09
3600	0.006	1.13
5400	0.008	1.18
7200	0.010	1.25

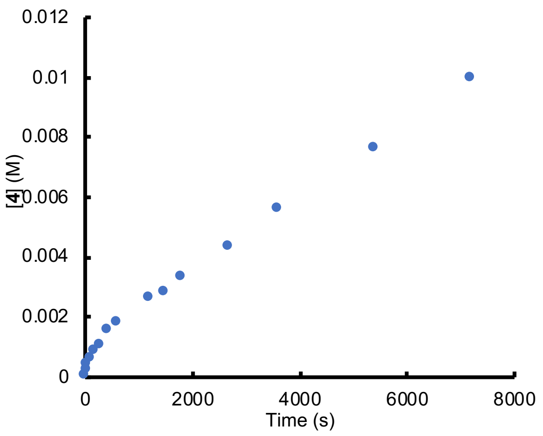

Table S18: Reaction data and linearization by general procedure 3. $[TESCl]_0 = 0.1$ M, $[NaI]_0 = 0.1$ M.

Lewis Acid	TBSCl, NaI	1 equiv, 1 equiv
k	0.000032	
krel	0.012	
Time (s)	[4] (M)	1/(1-F)
0	0.000	1.00
30	0.000	1.00
60	0.000	1.00
120	0.000	1.01
180	0.000	1.01
300	0.000	1.01
450	0.001	1.01
600	0.001	1.01
900	0.001	1.02
1200	0.001	1.02
1500	0.001	1.02
1800	0.001	1.03
2700	0.002	1.03
3600	0.002	1.04
5400	0.003	1.06
7200	0.003	1.07

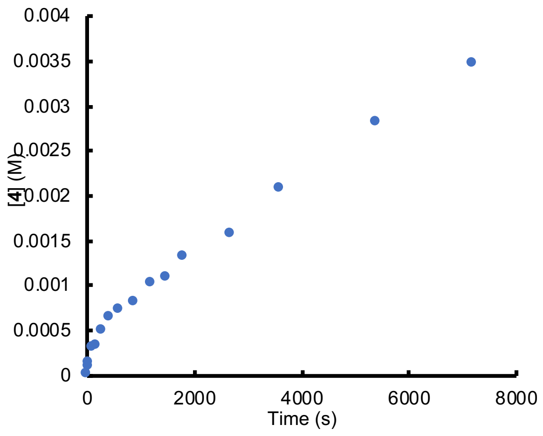

Table S19: Reaction data and linearization by general procedure 3. $[TBSCl]_0 = 0.1$ M, $[NaI]_0 = 0.1$ M.

Lewis acid	TMSCl	4 equiv
k	0.0006603	
krel	0.252	
Time (s)	[4] (M)	1/(1-F)
0	0.000	-1.00
30	0.001	-1.02
60	0.002	-1.04
120	0.003	-1.07
180	0.005	-1.10
300	0.007	-1.18
450	0.011	-1.29
600	0.014	-1.40
900	0.020	-1.68
1200	0.025	-2.01
1500	0.030	-2.47
1800	0.033	-2.92
2700	0.041	-5.44
3600	0.046	-13.42

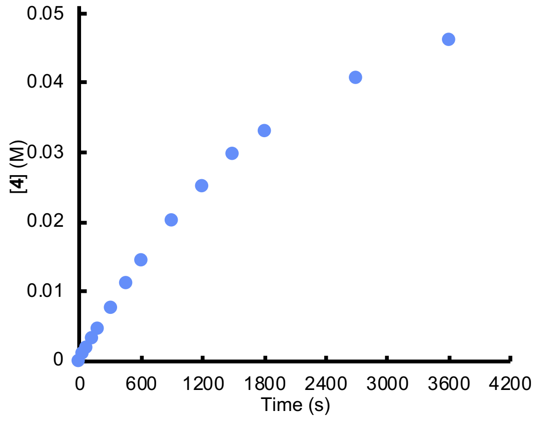

Table S20: Reaction data and linearization by general procedure 3. $[TMSCl]_0 = 0.4$ M.

4.2 Comparison of 1a and 2a Activation Rates by L1·$NiCl_2$

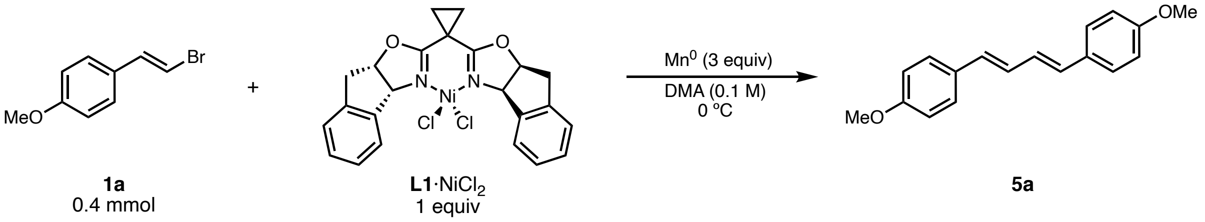

Reaction of L1·$NiCl_2$ with 1a: To an oven-dried 10 mL round bottom flask with a stir bar was added (*E*)-1-(2-bromovinyl)-4-methoxybenzene **1a** (85.2 mg, 0.40 mmol, 1 equiv) and Mn^0 powder (65.9 mg, 1.2 mmol, 3 equiv). In a 2-dram oven-dried vial with a stir bar was added **L1**$NiCl_2$ (1.1 times the needed amount, 213.4 mg, 0.44 mmol, 1.1 equiv). The flask and vial were then brought into a N_2-filled glovebox where 4.4 mL of DMA was added to the vial and the contents were stirred until homogenous to make a 0.1 M stock solution of **L1**·$NiCl_2$. To the flask was then added *n*-dodecane (target: 40 µL, 0.176 mmol, 0.44 eqiuv, actual mass was recorded for each experiment) internal standard was added followed by 4 mL of the **L1**·$NiCl_2$ stock solution. The flask was then sealed with a septa and electrical tape then removed from the glovebox where it was placed under N_2 and submerged in an ice bath. A ~50 µL aliquot of the solution was removed with a N_2-purged syringe then pushed through a SiO_2 plug and eluted with 10% EtOAc:hexanes into a GC vial then further diluted with EtOAc. Once cooled, the solution was then stirred at 1500 rpm to start the reaction (t = 0). The reaction was then aliquoted with the same procedure previously described at regular intervals.

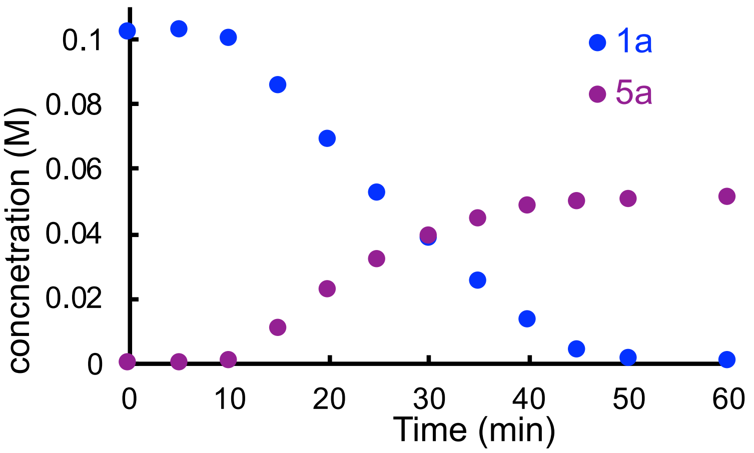

Reaction:	**1a** with **L1**NiCl2	
Time (min)	[1**a**] (M)	[**5a**] (M)
0	0.1025	0.0000
5	0.1030	0.0000
10	0.1003	0.0007
15	0.0859	0.0108
20	0.0689	0.0227
25	0.0528	0.0322
30	0.0388	0.0396
35	0.0255	0.0450
40	0.0134	0.0487
45	0.0043	0.0497
50	0.0014	0.0506
60	0.0007	0.0510

Figure S34: Reaction profile and tabulated data of stoichiometric reaction between **L1**·$NiCl_2$ and **1a**.

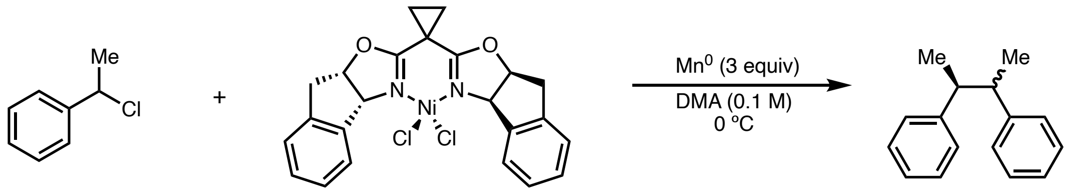

2a 0.4 mmol

L1NiCl$_2$ 1 equiv

4 (±) 1:1 dr

Reaction of L1·NiCl$_2$ with 2a: To an oven-dried 10 mL round bottom flask with a stir bar was added Mn^0 powder (65.9 mg, 1.2 mmol, 3 equiv). In a 2-dram oven-dried vial with a stir bar was added **L1**·NiCl$_2$ (1.1 times the needed amount, 213.4 mg, 0.44 mmol, 1.1 equiv). The flask and vial were then brought into a N$_2$-filled glovebox where 4.4 mL of DMA was added to the vial and the contents were stirred until homogenous to make a 0.1 M stock solution of **L1**·NiCl$_2$. To the flask was added (1-chloroethyl)benzene **2a** (53.1 µL, 0.40 mmol, 1 equiv) and *n*-dodecane (target: 40 µL, 0.176 mmol, 0.44 equiv, actual mass was recorded for each experiment) internal standard. The **L1**·NiCl$_2$ (4 mL) stock solution was then added to the flask before it was sealed with a septa and electrical tape then removed from the glovebox where it was placed under N$_2$ and submerged in an ice bath. A ~50 µL aliquot of the solution was removed with a N$_2$-purged syringe then pushed through a SiO$_2$ plug and eluted with 10% EtOAc:hexanes into a GC vial then further diluted with EtOAc. Once cooled, the solution was then stirred at 1500 rpm to start the reaction (t = 0). The reaction was then aliquoted with the same procedure previously described at regular intervals.

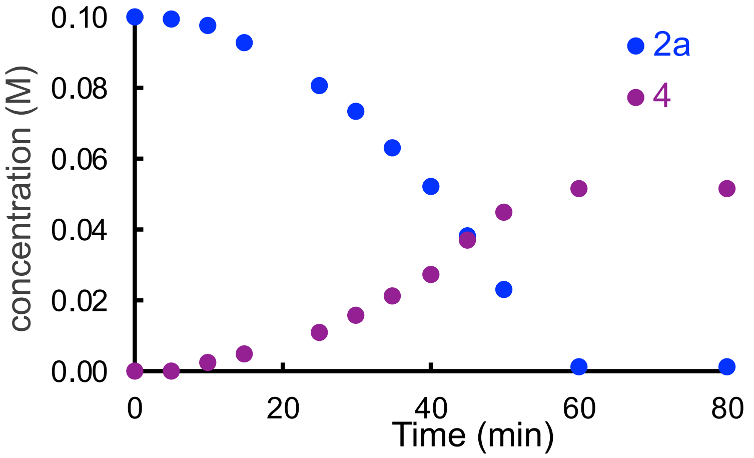

Reaction:	**2a** with **L1**NiCl2	
Time (min)	[**2a**] (M)	[**4**] (M)
0	0.0998	0.0000
5	0.0987	0.0000
10	0.0971	0.0021
15	0.0921	0.0046
25	0.0804	0.0108
30	0.0728	0.0153
35	0.0628	0.0210
40	0.0516	0.0272
45	0.0381	0.0367
50	0.0228	0.0446
60	0.0013	0.0511
80	0.0008	0.0513

Figure S35: Reaction profile and tabulated data of stoichiometric reaction between **L1**·NiCl$_2$ and **2a**.

Overlaid Reaction Profiles and Simulated Profiles

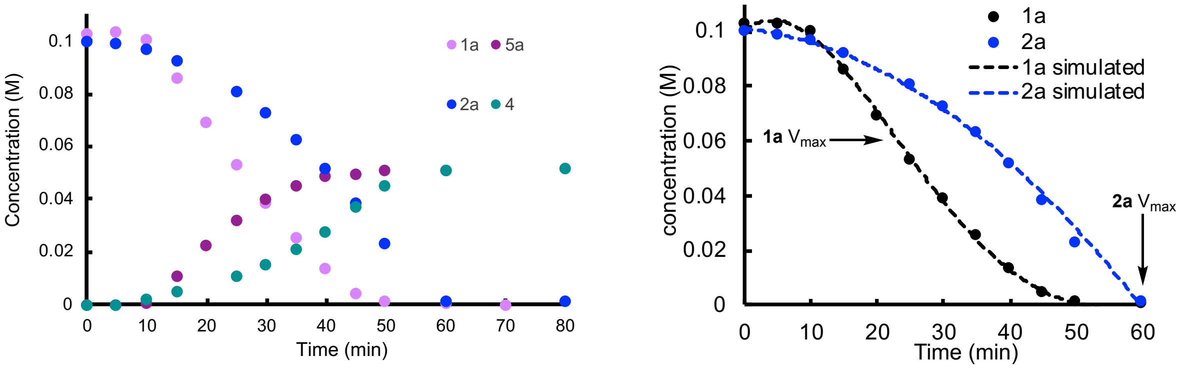

Figure S36: Left: Overlaid reaction profiles from the stoichiometric reaction of **L1**·$NiCl_2$ with **1a** and **2a** to compare the relative rates of electrophile activation. Right: Simulated reaction profiles to get relative rates.

Estimating k_{rel} for 1a and 2a Activation

To estimate the relative rates of **1a** and **2a** activation the concentration profiles were fitted with an appropriate n^{th}-order polynomial. The simulated profile from these equations (Figure S36, right) were then derived with the power rule to obtain an expression for d[**1a**]/dt and d[**2a**]/dt. Comparing the rates at 15% conversion gives a **1a**:**2a** k_{rel} = 4.1 whereas comparison of the maximum rates (V_{max}) gives a **1a**:**2a** k_{rel} = 1.3. These values are reasonable based on the reaction kinetic data that shows **1a** activation is faster yet **2a** is competitive at higher concentrations of **2a**.

4.3 Catalyst-Mediated 1a Activation Control Experiments

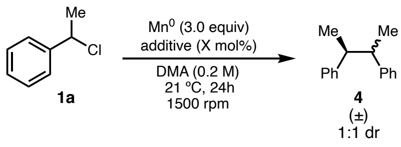

additive	**4** yield %
NA	ND
NaI (50 mol%)	< 5
$MnCl_2$ (10 mol%)	ND
**L1**$NiCl_2$ (10 mol%)	82
$NiCl_2$·dme (10 mol%)	63
**L1** (10 mol%)	ND

Procedure for Control Experiments: To an oven dried 1 dram vial with a stir bar was added Mn^0 powder (8.2 mg, 0.15 mmol, 3 equiv). The vial was then brought into a N_2-filled glovebox where (1-chloroethyl)benzene **2a** (6.6 µL, 0.050 mmol, 1 equiv), *n*-dodecane internal standard, and the respective additive (if applicable) was added. DMA (0.25 mL, 0.2 M) was then added to the vial before it was sealed with a teflon-lined cap and removed from the glovebox. The

reactions were allowed to stir at ambient temperature for 24h at 1500 rpm. Upon completion the crude reaction mixture was filtered through SiO_2 plug and eluted with 10% EtOAc:hexanes into a GC vial then further diluted with EtOAc, then analyzed by GC-FID. Reaction were run in duplicate and no other **2a**-derived byproducts (other than **4**) were detected.

These control experiments show that Ni is required for **2a** activation meaning a reductant-mediated activation pathway, like **2b**, is unlikely.

4.4 Preliminary Kinetic Simulations of Rate-Controlling Activation of NHP Esters

Discussion: We became interested in modeling[8] the rate-determining NHP reduction reaction to account for the reaction profile shape from the kinetics experiments on Lewis acid-mediated NHP ester reduction (SI Section 4.1), the full catalytic reaction (GC kinetics SI Section 3.2 and NMR reaction monitoring SI section 6.1). Specifically, we noticed in most cases the reaction displays an initial "burst" then enters what appears to be a more linear region. We believe that this shape is to be expected based on our mechanistic hypothesis of reversible silylation of NHP ester by TMSX followed by rate-limiting TDAE reduction. A steady state approximation of the rate law for this process is shown below (Scheme S1).

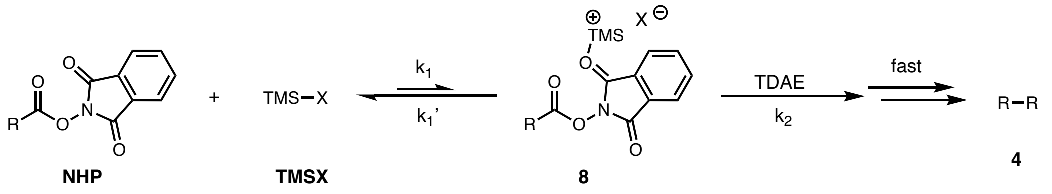

steady state approximation: $$\frac{d[\mathbf{4}]}{dt} = \frac{k_1 k_2[\mathrm{TDAE}][\mathbf{NHP}][\mathbf{TMSBr}]}{k_1'[\mathbf{X}^-] + k_2[\mathrm{TDAE}]}$$

*[**8**]$_t$ is dependent on Lewis acid (K_1) and its concentration*

Scheme S1: Simplified reaction model used for kinetic simulations and rate law assuming a steady state scenario with respect to **8**.

This model uses similar conditions to those in the NHP ester reduction studies in Figure 5 ($[NHP]_0$ = 0.1 M, $[TDAE]_0$ = 0.2 M, $[TMSX]_0$ = 0.1 M) and assumes that the silylation of the NHP ester is endergonic (K_1<1) with faster reduction by TDAE (k_2>k_1). From the

parameters $k_1 = 0.1$, $k_1' = 1$, $k_2 = 100$, we observe a similar profile to the observed reactions with a fast burst of reactivity and a flatter surface at high conversions due to low $[TMSX]_t$ (purple, Figure S37). Changing K_1 to 0.01 ($k_1 = 0.01$, $k_1' = 1$) flattens the curve to something that becomes more linear (teal, Figure S37). The rate can then be increased by increasing the initial concentration of TMSX (green, Figure S37), with an overall similar curvature. By analogy we can make similar perturbations to kinetics parameters by changing the Lewis acid (K_1) and the initial concentration of a given Lewis acid ($[TMSX]_t$). Gratifyingly, we observe a similar trend to that predicted by this kinetic model when changing the equilibrium constants by moving from TMSBr to TMSCl ($k_{rel} = 0.91$); the curve significantly flattens when using 1 equiv TMSCl instead of 1 equiv TMSBr, but the rate can be accelerated by using a higher a concentration of $[TMSCl]_0$ (full profile in the middle and the first 2000 s of the reactions on the right for clarity, Figure S38). We note that the rate-controlling step of NHP ester reduction is the same as for the catalytic reaction and similar profile shapes are observed for both experiments.

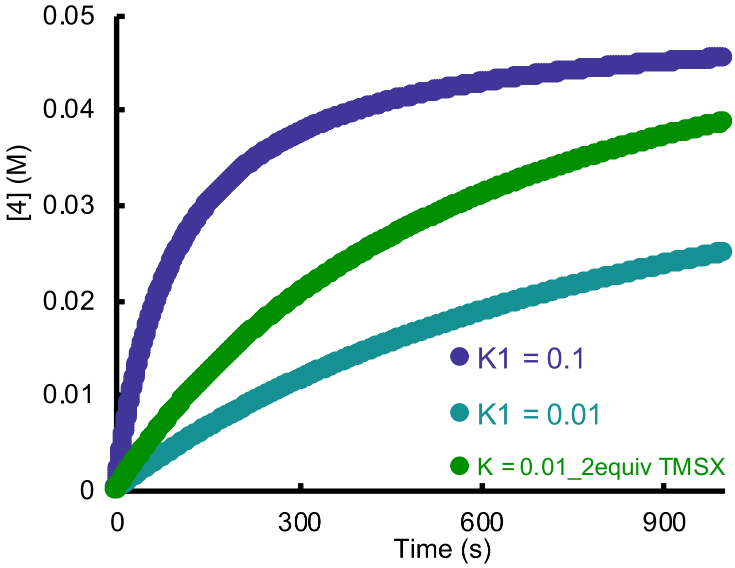

Figure S37: Simulated product formation profiles for the NHP ester reduction reaction obtained from preliminary kinetic models using different initial conditions.

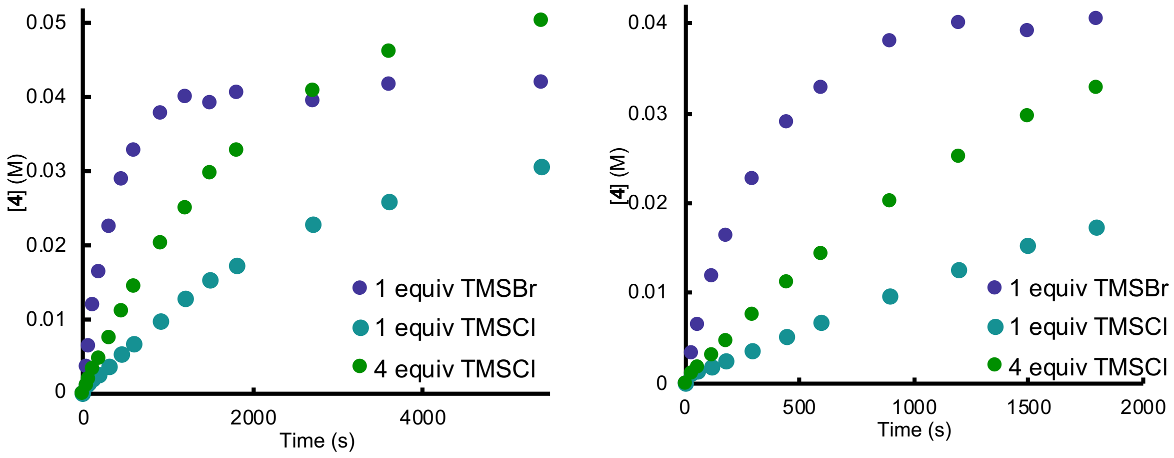

Figure S38: Experimental product formation profiles from SI section 4.1. The differences in the full reaction profile (left) and initial rates (right) from using different Lewis acids (K_1) and starting concentration ($[TMSX]_0$) are intended to replicate the simulated data shown in Figure S37.

5. Cyclic Voltammetry Experiments

General Details: Cyclic voltammograms were obtained in a N_2-filled glovebox using a standard three electrode cell consisting of a freshly polished (0.3 µm then 0.05 µm alumina) glassy carbon working electrode, platinum counter electrode, and a silver wire non-aqueous reference electrode containg a 10 mM $AgNO_3$, 0.1 M $TBAPF_6$, MeCN filling solution. Data were collected using a Biologic SP-300 potentiostat and analyzed in EC-Lab. All cyclic voltammograms were measured in DMA with 0.1 M $TBAPF_6$ or 0.1 M $TBAClO_4$ supporting electrolyte and then referenced to freshly sublimed ferrocene (Fc). $TBAPF_6$ was recrystallized from boiling absolute ethanol and stored in a N_2-filled gloveox. The reduction potentials are reported versus the reduction potential of the Fc/Fc^+ peak. Ohmic drop compensation was done with all samples before each scan using positive-feedback iR-compensation at 85% of uncompensated resistance (R_u) measured from potentio electrochemical impedence spectroscopy (PEIS). The first scan is shown in the following section and the main text unless otherwise specified.

5.1. CVs of $L1NiX_2$ complexes, 2b, and TDAE

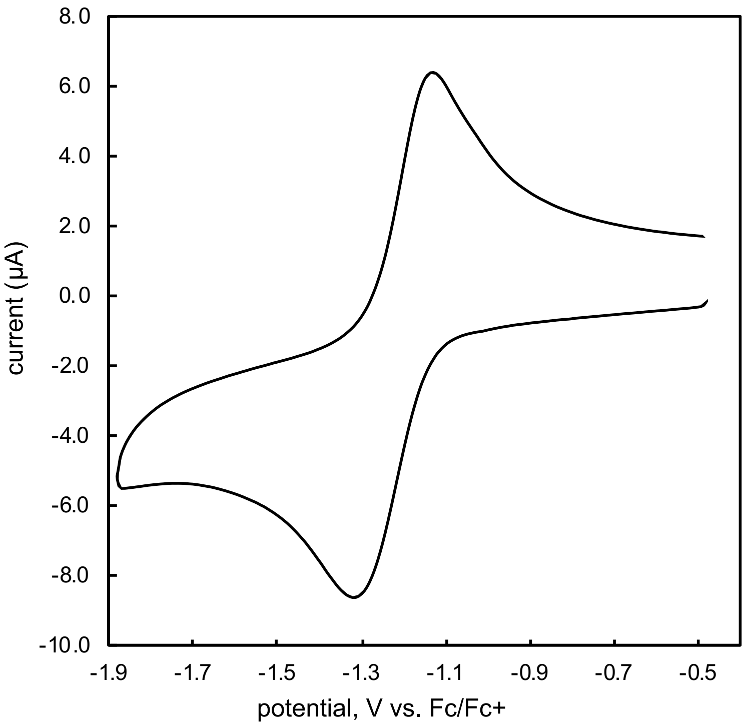

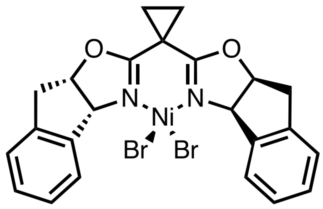

Analyte	**L1**NiBr2
Epc (Fc/Fc+)	– 1.32 V
Epc/2 (Fc/Fc+)	– 1.23 V
Epa (Fc/Fc+)	– 1.05 V

Figure S39: Voltammogram of 1 mM **L1**·$NiBr_2$ in DMA with 0.1 M $TBAPF_6$ supporting electrolyte, ν = 100 mV/s.

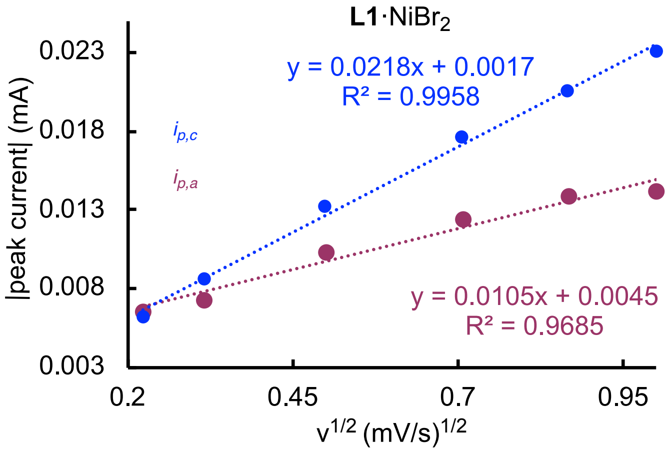

Figure S40: Randles-Sevcik plot of **L1**·$NiBr_2$ obtained from varying the scan rate.

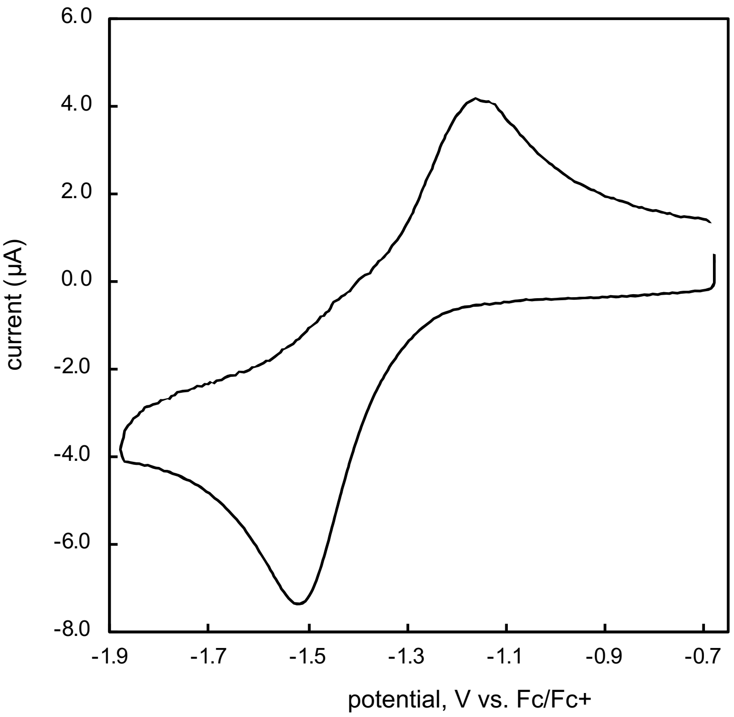

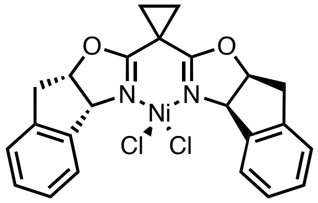

Analyte	**L1**NiCl2
Epc (Fc/Fc+)	– 1.52 V
Epc/2 (Fc/Fc+)	– 1.47 V
Epa (Fc/Fc+)	– 1.17 V

Figure S41: Voltammogram of 1 mM **L1**·$NiCl_2$ in DMA with 0.1 M $TBAPF_6$ supporting electrolyte, ν = 100 mV/s.

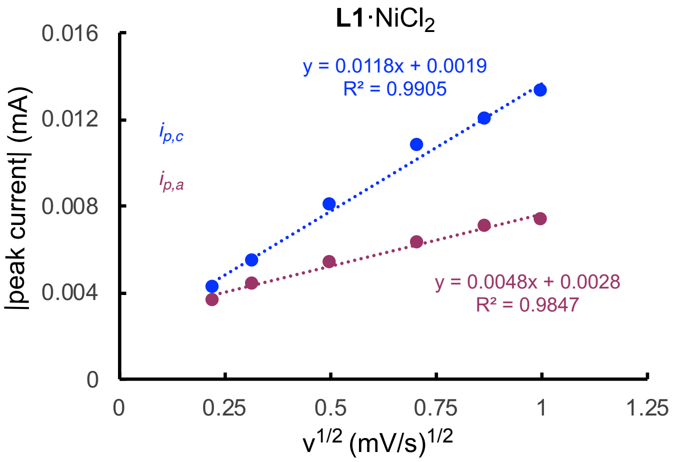

Figure S42: Randles-Sevcik plot of **L1**·$NiCl_2$ obtained from varying the scan rate.

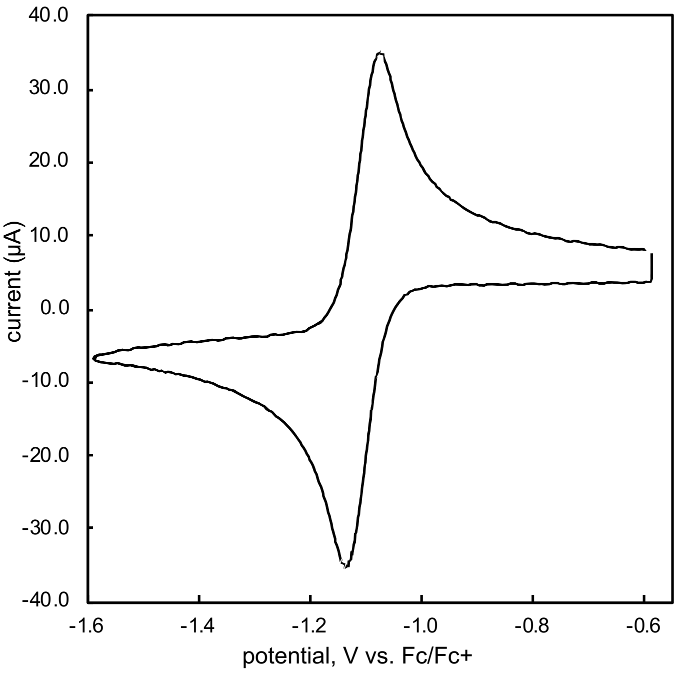

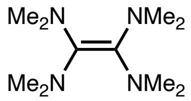

Analyte:	TDAE
E1/2(Fc/Fc+)	-1.11 V

Figure S43: Voltammogram of 1 mM TDAE in DMA with 0.1 M $TBAPF_6$ supporting electrolyte, $\nu = 100$ mV/s.

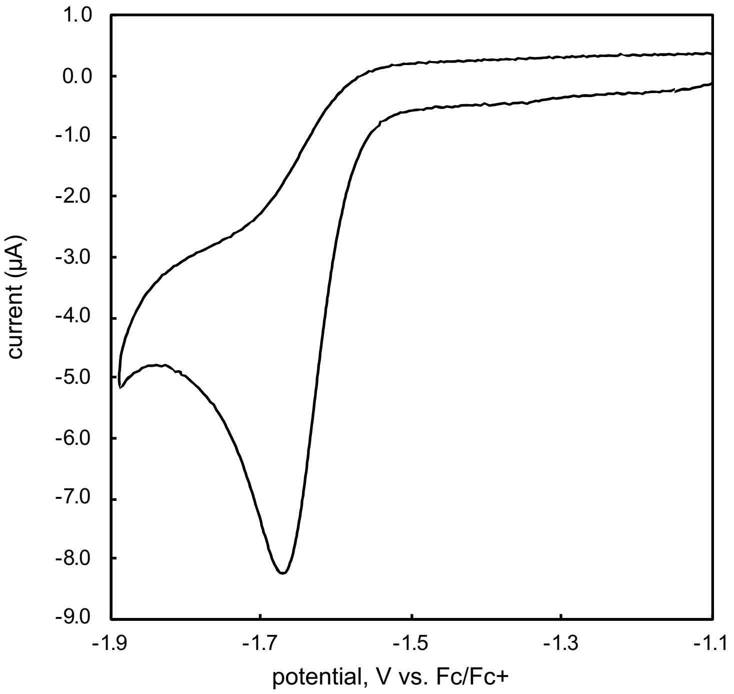

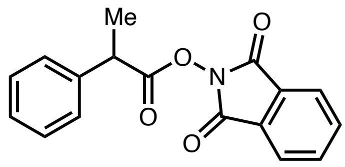

Analyte:	**2b**
Ep (Fc/Fc+)	-1.67 V
Ep/2 (Fc/Fc+)	-1.61 V

Figure S44: Voltammogram of 1 mM **2b** in DMA with 0.1 M $TBAPF_6$ supporting electrolyte, $\nu = 100$ mV/s.

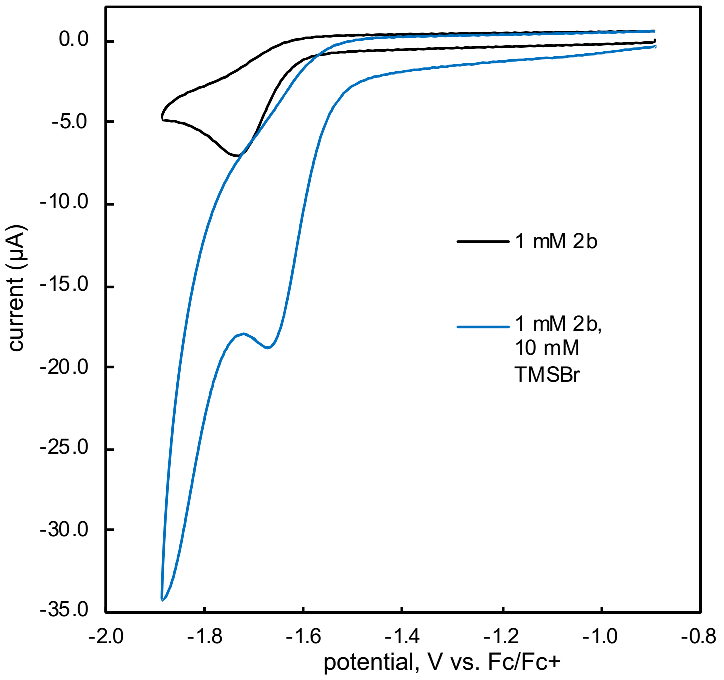

Figure S45: Effect of TMSBr on **2b** reduction. 1 mM **2b** and 10 mM TMSBr in DMA with 0.1 M TBAClO$_4$ supporting electrolyte, ν = 100 mV/s.

5.2. Substrate Titration and Catalytic Current Comparison

Procedure for Substrate Titration for Current response of L1·NiCl$_2$: For these experiments CVs were taken of 1 mM **L1**·NiCl$_2$ followed by the addition of an appropriate amount of **1a** or **2a** was added for subsequent scans. At the end of each titration the substrate that was not previously titrated in was then added in equimolar amounts.

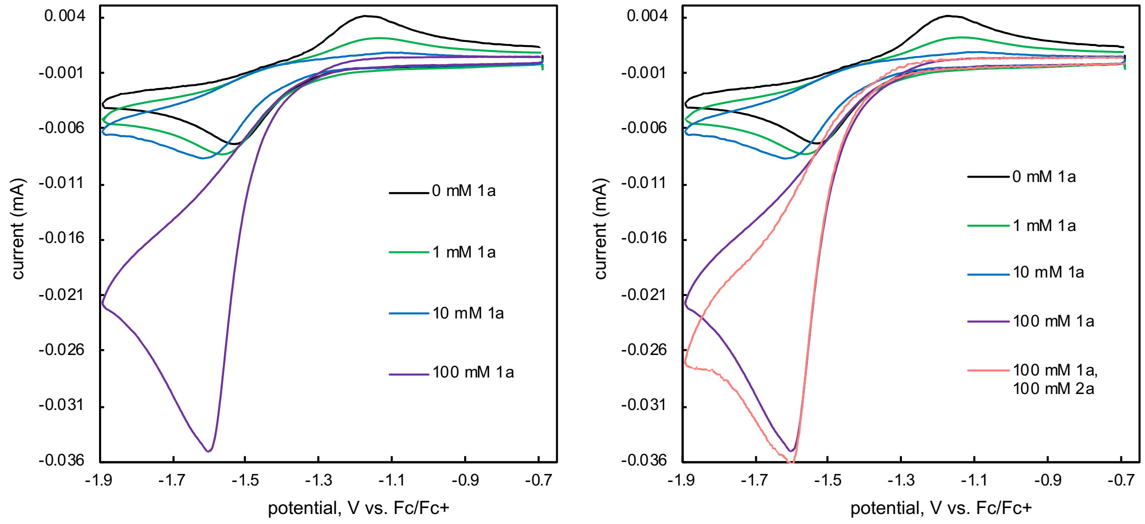

Figure S46: CV of 1 mM **L1**·$NiCl_2$ with increasing concentrations of **1a** (left) and subsequent addition of 100 mM **2a** (right). CVs run in DMA with0.1 M $TBAPF_6$ supporting electrolyte, ν = 100 mV/s.

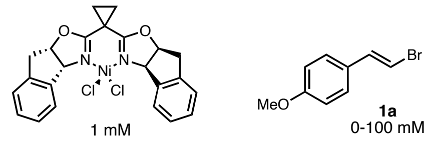

[**1a**] (M)	*i* p,c (μA)	*i* p,a (μA)	peak ratio (*i* p,a/*i* p,c)
0	6.88	4.66	0.68
1	7.10	2.24	0.32
10	8.03	0.21	0.03
100	34.89	0.00	–

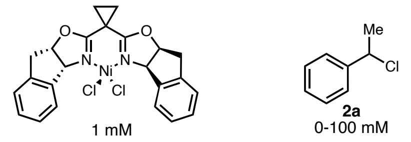

[**2a**] (M)	*i* p,c (μA)	*i* p,a (μA)	peak ratio (*i* p,a/*i* p,c)
0	6.88	4.66	0.68
1	5.48	3.03	0.55
10	5.12	0.72	0.14
100	20.05	0.00	–

Table S21: Comparison of peak current ratios of **L1**·$NiCl_2$ reacting with **1a** (left) and **2a** (right) by CV.

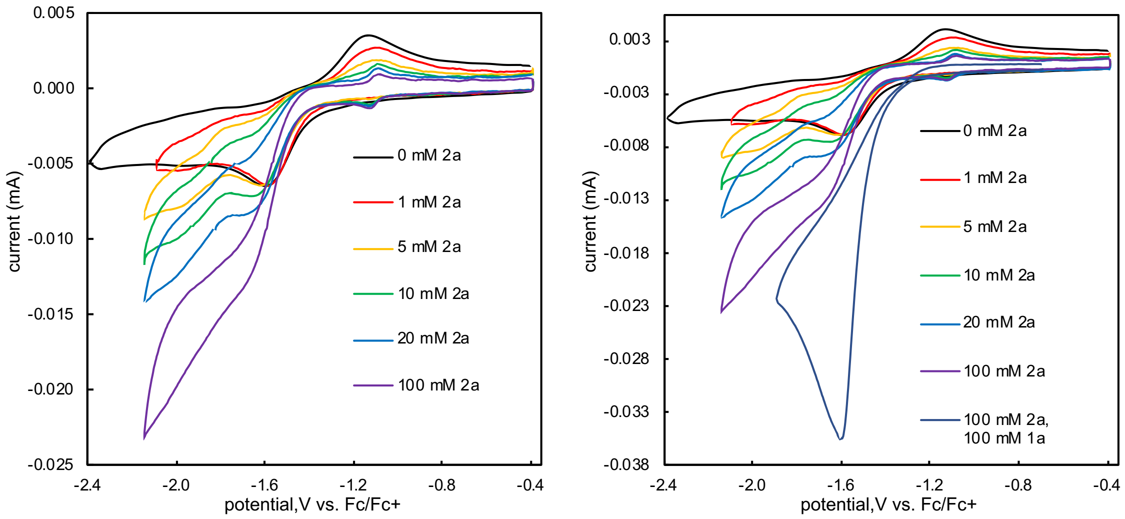

Figure S47: CV of 1 mM **L1**·$NiCl_2$ with increasing concentrations of **2a** (left) and subsequent addition of 100 mM **1a** (right). CVs run in DMA with 0.1 M $TBAPF_6$ supporting electrolyte, ν = 100 mV/s.

6. NMR Reaction Monitoring

6.1. ^{19}FNMR Reaction Monitoring

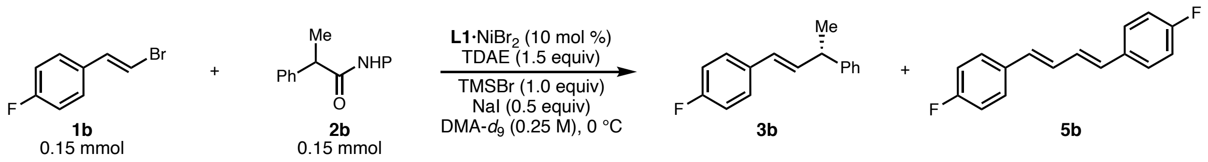

General procedure for ^{19}F Reaction Monitoring: In a N_2-filled glovebox (*E*)-1-(2-bromovinyl)-4-fluorobenzene **1b** (30.2 mg, 0.15 mmol, 1 equiv), 1,3-dioxoisoindolin-2-yl 2-phenylpropanoate **2b** (44.3 mg, 0.15 mmol, 1 equiv), **L1**$NiBr_2$ (8.6 mg, 0.015 mmol, 0.1 equiv), NaI (11.2 mg, 0.075 mmol, 0.5 equiv), and hexafluorobenzene internal standard were added to a dry 1 dram vial with a stir bar. The contents were then dissolved in 0.3 mL of DMA-d^9 then transferred to a dry J-young NMR tube. The vial was rinsed with 0.3 mL of DMA (0.6 mL final volume, 0.25 M final concentration) to ensure quantitative transfer. The NMR tube was then sealed and removed from the glovebox where it was then cooled to 0 ºC in the NMR instrument (air bath cooling to 0 ºC). The sample was locked/shimmed and an initial quantitative ^{19}F NMR measurement was taken in order to determine starting concentration. The tube was then removed from the instrument, submerged in an ice bath, fitted with a septa, and Ar balloon. The tube was opened then TMSBr (19.8 uL, 0.15 mmol, 1 equiv) was added via syringe and the tube was agitated to ensure adequate mixing. After 30 seconds TDAE (52.3 µL, 0.225 mmol, 1.5 equiv) was added to start the reaction and the tube was immediately sealed, placed back in the NMR instrument, and qNMR (single scan, 27s interscan delay) scans (measured 3 minute delay from TDAE addition to completion of first measurement). Measurements were taken every 30 seconds for the first 3 hours of the reaction and then every minute for the next 3 hours (6 hours total). At the end of the reaction the product was isolated to determine ee of **3b** as 93% by SFC analysis (OJ-H, 7% IPA:CO_2, t_{major} = 7.07 min, t_{minor}=5.86 min).[5]

Modifications to General Procedure for 20 mol % Catalyst Loadings: For this experiment more **L1**$NiBr_2$ (17.2 mg, 0.030 mmol, 0.2 equiv) added to 1 dram vial. The delay between TDAE addition and the first scan was shortened to one minute instead of three minutes. Scan taken every one minute for the entire experiment and the reaction was monitored for 5 hours total.

Important Considerations for this Experiment: It is apparent when comparing the reaction profiles for the NMR-based kinetics (Figure 4c) and GC-FID-based kinetics (Figures 2b-d) the shapes of the profiles are significantly different. We attribute this to several factors beyond differences in alkenyl bromide electronics and reaction concentration. In addition to running under different conditions, the GC kinetics are run under N_2 connected a bubbler (Schlenk line) and are stirred for the entirety of the reaction while the NMR experiments are run in a sealed J-young tube and are only agitated in the beginning of the reaction to encourage mixing. These factors are likely significant for a reaction that has two separate phase changes associated with

the rate-controlling step: 1) CO_2 evolution and 2) precipitation of $[TDAE]X_2$ salts. Qualitatively we observe that the precipitate is microcrystalline in the GC reaction but large single crystals are present in the NMR tube by the end of the experiment. Perhaps the most significant difference is how well we can control the temperature at the beginning of each reaction. Great care and optimization went into the ^{19}F NMR reaction experiments to ensure that the NMR tube is kept at 0 ºC for as long as possible including pre-cooling of the probe and submerging the tube in an ice bath while it is outside the instrument. Despite these considerations there is still a 30-45 second window where the tube is transferred from the ice bath following TDAE addition back into the instrument itself which gives this reaction, in a reactor with a high surface area to volume ratio time to warm a bit at early points. A temperature jump is not observed on the measured temperature of the probe as the tube is added but this does not mean the reaction itself doesn't briefly warm.

Representative Spectra (full window):

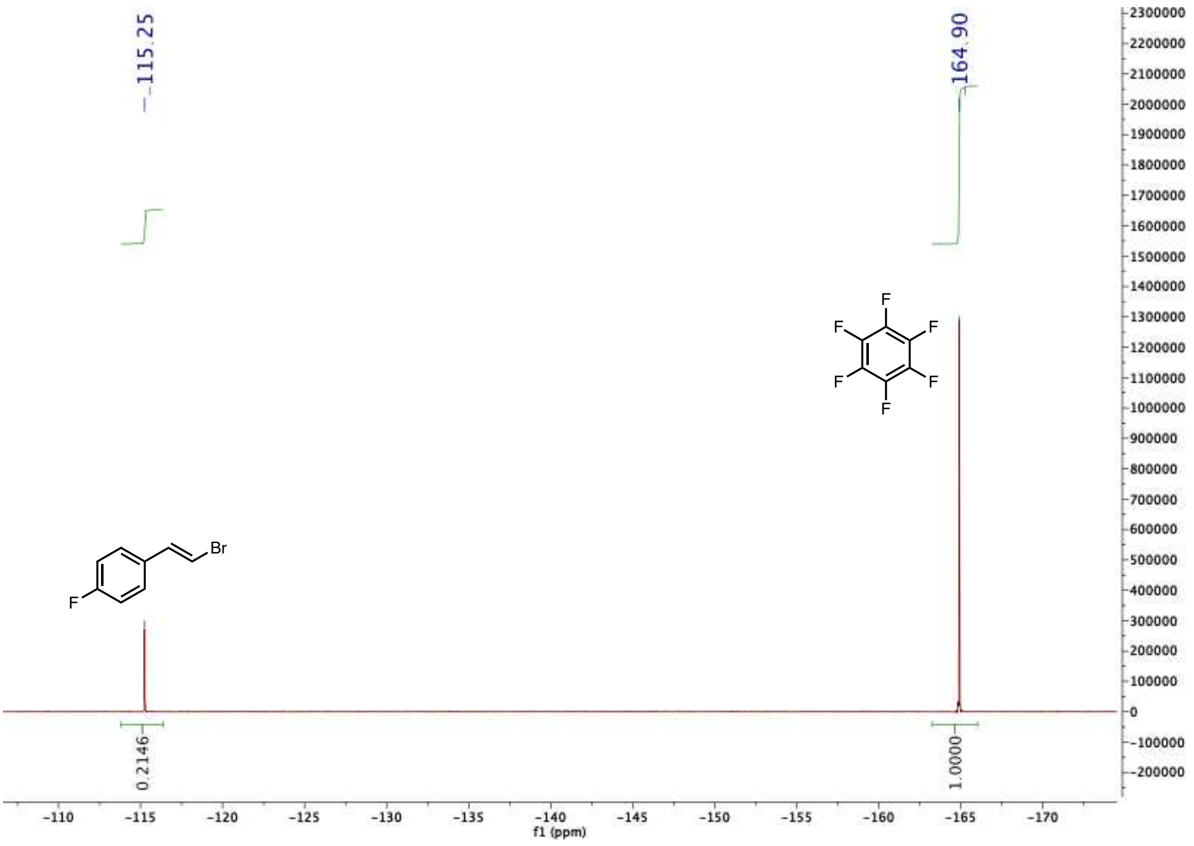

Figure S48: ^{19}F NMR full window view of reaction mixture before TDAE is added.

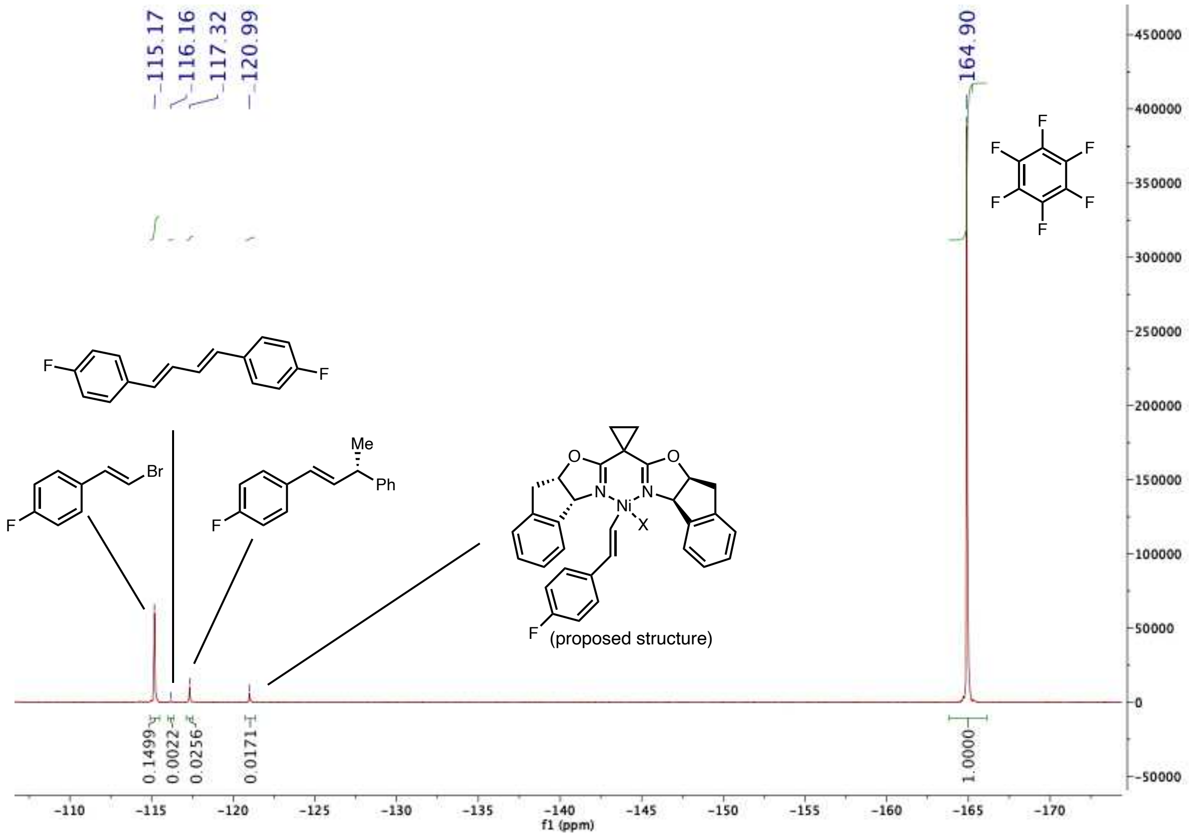

Figure S49: ^{19}F NMR full window view of reaction mixture 3 minutes after TDAE was added.

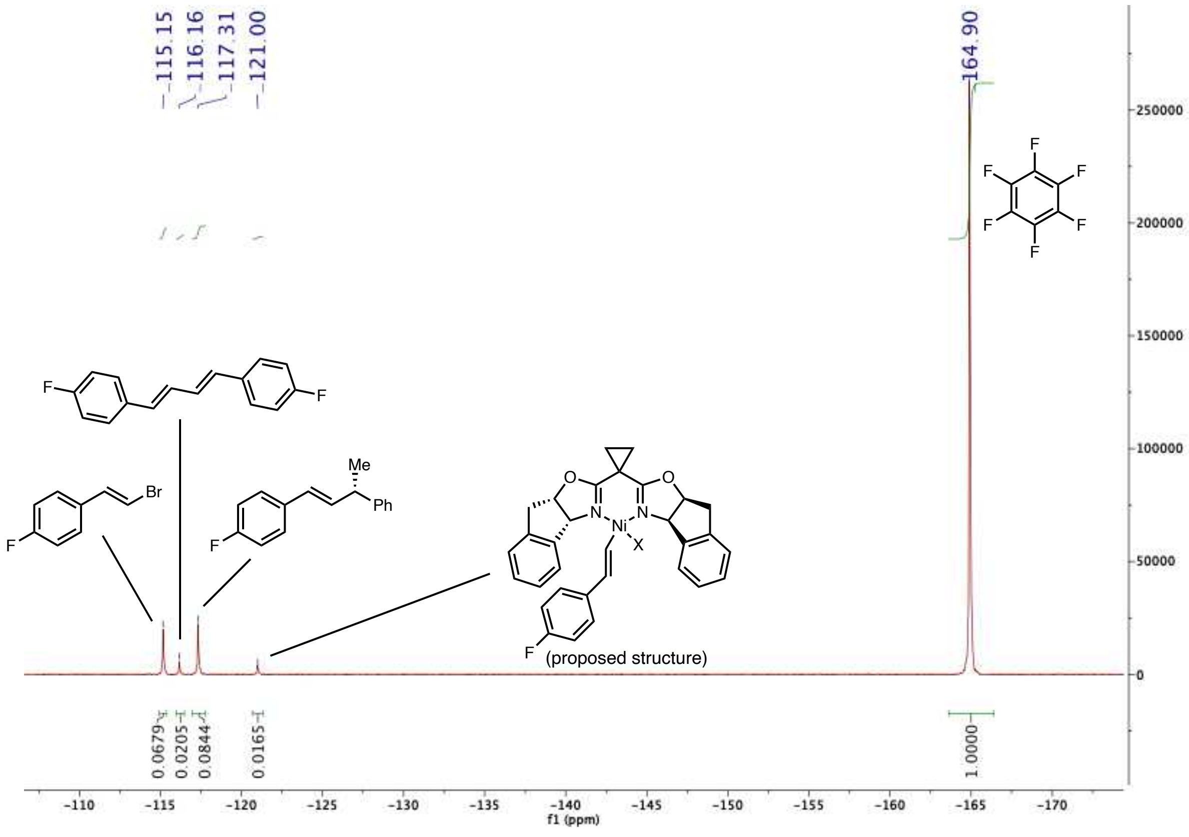

Figure S50: ^{19}F NMR full window view of reaction mixture 3 hours after TDAE was added.

Independently prepared, C_6F_6-referenced (C_6F_6 = -164.9 ppm) NMR characterization in $CDCl_3$ of possible **1b**-derived species. This includes species like 4-fluorostyrene and **1b-I** that were not observed in the reaction mixture. It is noteworthy that while the absolute chemical shifts of independently prepared species in $CDCl_3$ are different than those observed in the reaction mixture in DMA-d_9 (Figure S48-50), the relative shifts are the same (Table S22).

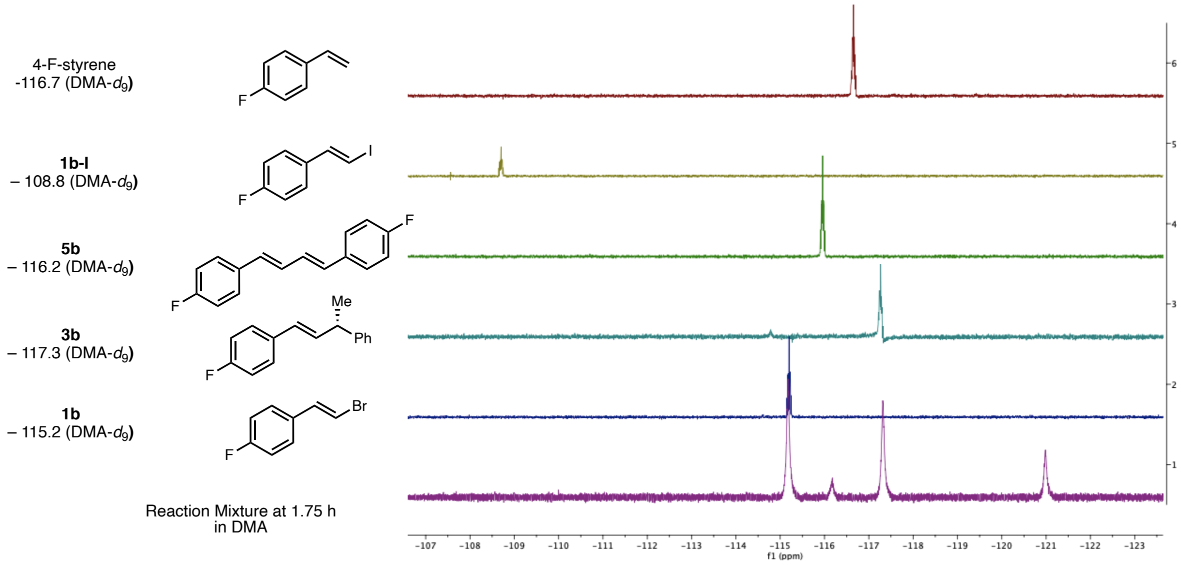

Figure S51: ^{19}F chemical shifts for reaction components and potential byproducts. No species corresponds to observed intermediate that is previously assigned to resting state species. Reported shifts are referenced such that C_6F_6 =-164.9 ppm.

	4-F-styrene	**1b-I**	**5b**	**3b**	**1b**	Resting State
CDCl3 (ppm)	-117.5	-110.8	-117.3	-118.7	-116.2	ND
DMA-d9 (ppm)	-116.7	-108.8	-116.2	-117.3	-115.2	-120.99
Δ to **1b** ppm (CDCl3)	-1.3	5.4	-1.1	-2.5	0	–
Δ to 1b ppm (DMA-d9)	-1.5	6.4	-1	-2.1	0	-5.79

Table S22: Tabulated ^{19}F NMR shifts in $CDCl_3$ and DMA-d_9 as well as the relative shifts compared to **1b**.

Δ to C_6F_6 (DMA)	**1b**	**5b**	**7b**
Observed	51.00	50.00	45.00
DFT Predicted	47.00	46.00	37.00

Table S23: Comparison between observed and DFT predicted ^{19}F chemical shifts.

Processed Reaction Data:

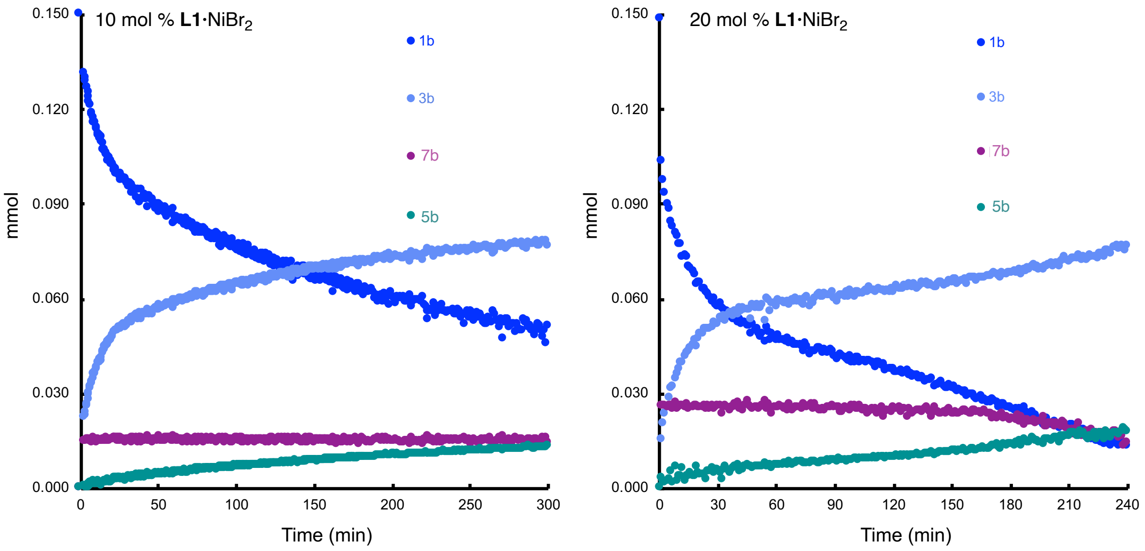

species	% yield at 300 min*
**1b**	33
**3b**	52
**7b**	10
**5b**	8

** end time of montering this experiment*

species	% yield at 240 min*
**1b**	9
**3b**	51
**7b**	10
**5b**	12

** end time of montering this experiment*

Figure S52: Quantified amount of each species for ^{19}F time courses shown in main text.

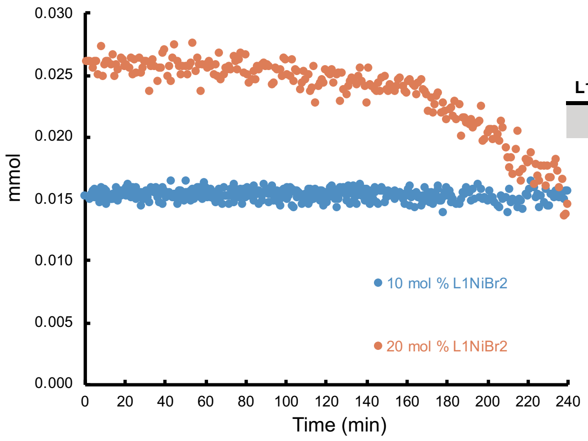

**L1**NiBr$_2$ loading	Avg **L1**NiBr$_2$ (mmol/mol %)	Std Dev (mmol)	Period Used for Avg
10 mol %	0.015 / 10.1	0.0005	3 min – 5 h
20 mol %	0.025 / 16.9	0.0008	1 min – 2 h

Figure S53: Comparison of resting state concentration for experiments starting with 10 mol % and 20 mol % **L1**·NiBr$_2$. Table showing calculation of average amount of the proposed resting state species.

6.2. Room Temperature ^{19}F NMR

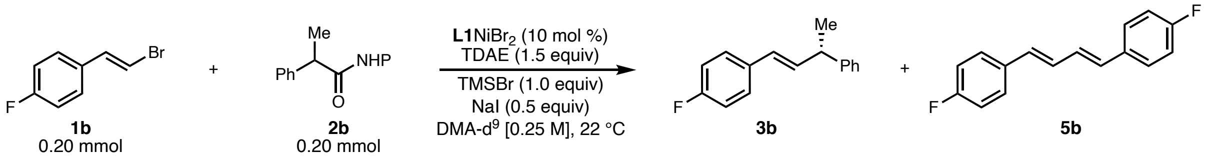

Ambient Temperature NMR Reaction: In a N_2-filled glovebox (*E*)-1-(2-bromovinyl)-4-fluorobenzene **1b** (40.2 mg, 0.20 mmol, 1 equiv), 1,3-dioxoisoindolin-2-yl 2-phenylpropanoate **2b** (59.1 mg, 0.2 mmol, 1 equiv), **L1**·$NiBr_2$ (11.5 mg, 0.020 mmol, 0.1 equiv), NaI (15.0 mg, 0.10 mmol, 0.5 equiv), and hexafluorobenzene internal standard were added to a dry 1 dram vial with a stir bar. The contents were then dissolved in 0.4 mL of DMA-d_9 then transferred to a dry J-young NMR tube. The vial was rinsed with 0.4 mL of DMA (0.6 mL final volume, 0.25 M final concentration) to ensure quantitative transfer. The NMR tube was then sealed and removed from the glovebox. The sample was locked/shimmed and an initial quantitative ^{19}F NMR measurement was taken in order to determine starting concentration. The tube was then removed from the instrument, fit with a septa, and Ar balloon. To the tube was then added TMSBr (26.4 µL, 0.20 mmol, 1 equiv) was added via syringe and the tube was agitated to ensure adequate mixing. After 30 seconds TDAE (69.8 µL, 0.30 mmol, 1.5 equiv) was added to start the reaction and the tube was immediately sealed, placed back in the NMR instrument and monitored at 22 ºC.

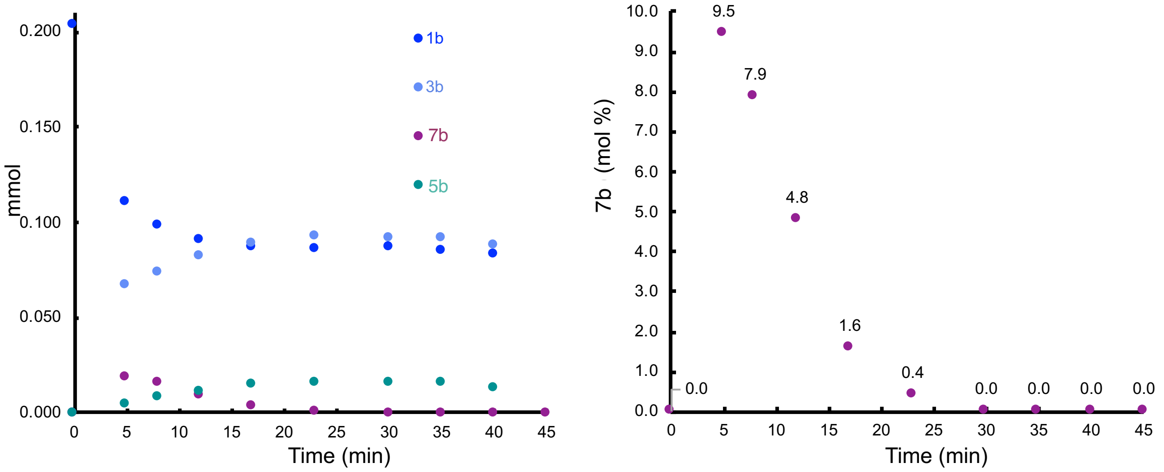

Figure S54: Concentration of **1b**-derived species over the course of the reaction (left). Concentration of putative resting state species **7b** during the time course (right).

6.3. Attempts to detect 7b by 1H NMR

Discussion:

We have conducted experiments monitoring the reaction mixture by alternating ^{1}H NMR and ^{19}F NMR. In this study we observe ^{1}H NMR signals in the diamagnetic region (Figure S58) that track with the proposed catalyst resting state signal by ^{19}F NMR (Figure S55). These signals are not well resolved making a complete assignment challenging but we have attempted to assign a pair of signals corresponding to the alkenyl bromide fragment and the ligand backbone. We note that no paramagnetic signals corresponding to **L1**·$NiBr_2$ are observed during active catalysis or at the end of the reaction (Figure S56-57). To aid in this assignment we prepared and characterized the **L1**·$ZnCl_2$ complex by NMR in DMF-d_7 to see how the ligand chemical shifts change upon coordination to a diamagnetic metal (Figure S63-65). We observe significant deshielding of protons on the ligand upon coordination, in particular the aryl protons in closest proximity to the metal center (signal at 7.9 ppm). A similar apparent doublet at ~9.3 ppm is observed in the reaction mixture for the in situ NMR experiment, which integrates 1:1 relative to alkenyl proton signals (7.05 and 5.42 ppm) and supports a diamagnetic Ni^{II} resting state (Figure S62).

Procedures and data:

Ambient Temperature NMR Reaction (dilute variant of previous experiment): In a N_2-filled glovebox (*E*)-1-(2-bromovinyl)-4-fluorobenzene **1b** (5.0 mg, 0.025 mmol, 1 equiv), 1,3-dioxoisoindolin-2-yl 2-phenylpropanoate **2b** (7.4 mg, 0.025 mmol, 1 equiv), **L1**·$NiBr_2$ (1.4 mg, 2.5 μmol, 0.1 equiv), NaI (1.9 mg, 0.0125 mmol, 0.5 equiv), and hexafluorobenzene internal standard were added to a dry 1 dram vial with a stir bar. The contents were then dissolved in 0.4 mL of DMA-d_9 then transferred to a dry J-young NMR tube. The vial was rinsed with 0.3 mL of DMA (0.7 mL final volume, 0.35 mM final concentration) to ensure quantitative transfer. The NMR tube was then sealed and removed from the glovebox. The sample was locked/shimmed and an initial ^{1}H and ^{19}F NMR was taken before the reaction was initiated. The tube was then removed from the instrument, fit with a septa, and Ar balloon. To the tube was then added TMSBr (3.3 μL, 0.025 mmol, 1 equiv) was added via syringe and the tube was agitated to ensure adequate mixing. After 30 seconds TDAE (8.7 μL, 0.0375 mmol,

1.5 equiv) was added to start the reaction and the tube was immediately sealed, placed back in the NMR instrument and monitored at 22 ºC by ^{19}F and ^{1}H NMR.

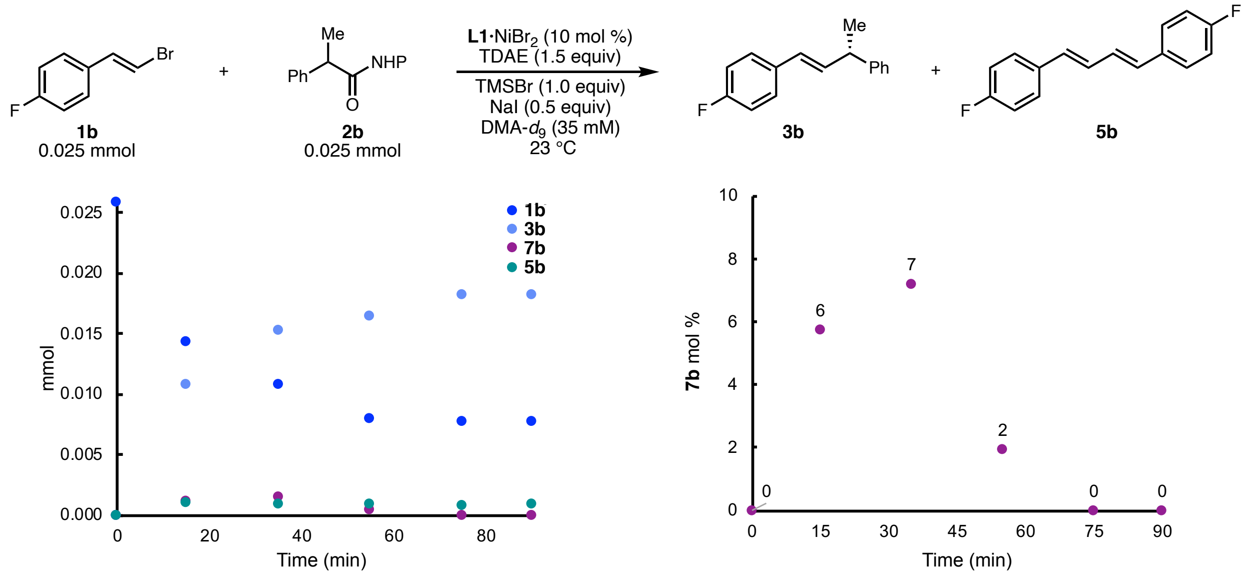

Figure S55: Concentration of **1b**-derived species over the course of the reaction (left). Concentration of putative resting state species **7b** during the time course (right).

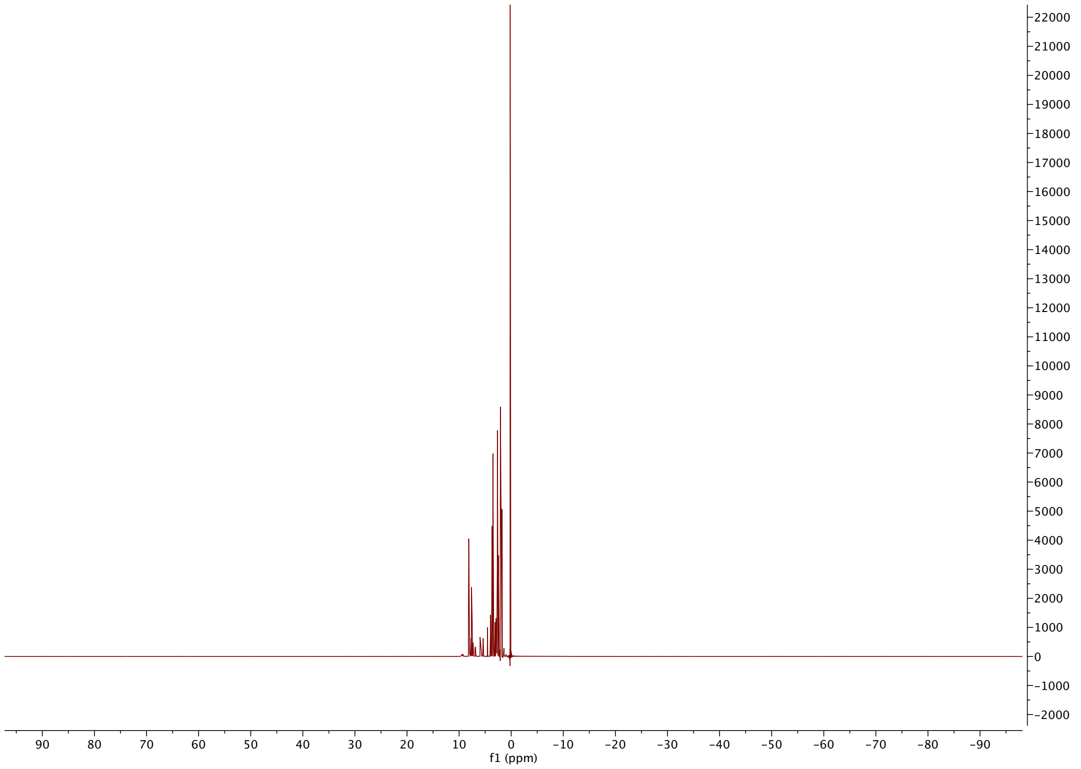

Figure S56: Full ^{1}H NMR window of the reaction mixture at 25 minutes.

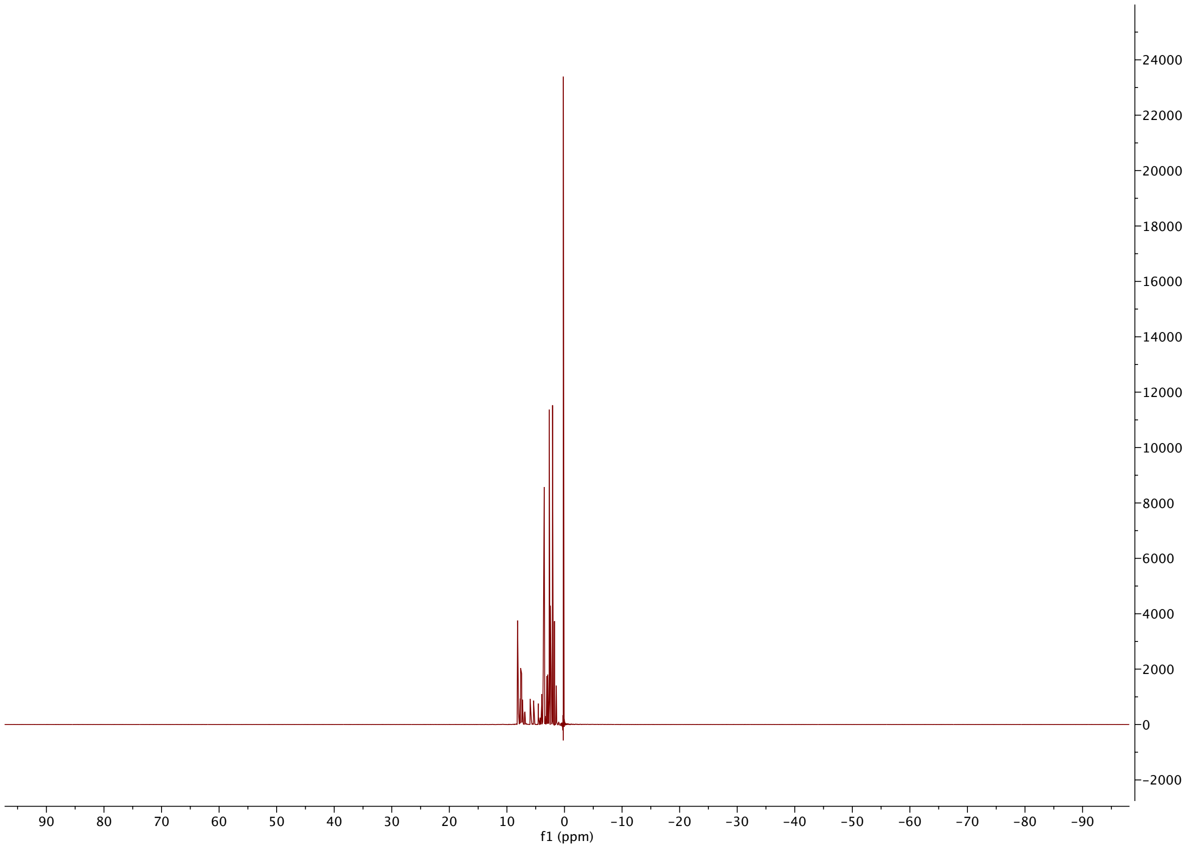

Figure S57: Full ^{1}H NMR window of the reaction mixture at 85 minutes.

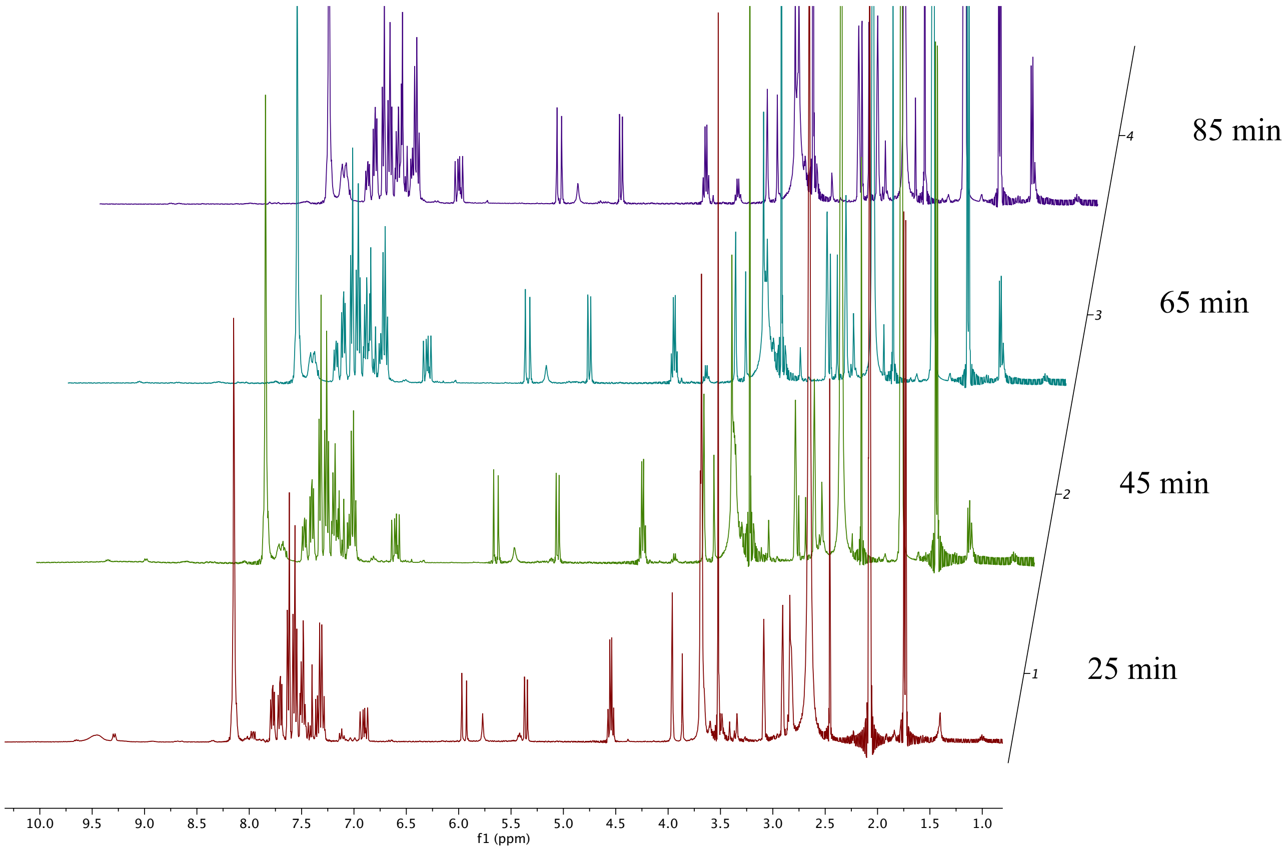

Figure S58: ^{1}H NMR time course of the reaction. When comparing to Figure S55, the 25 and 45 minute time points should contain the proposed resting state species while the 65 and 85 minute time points do not.

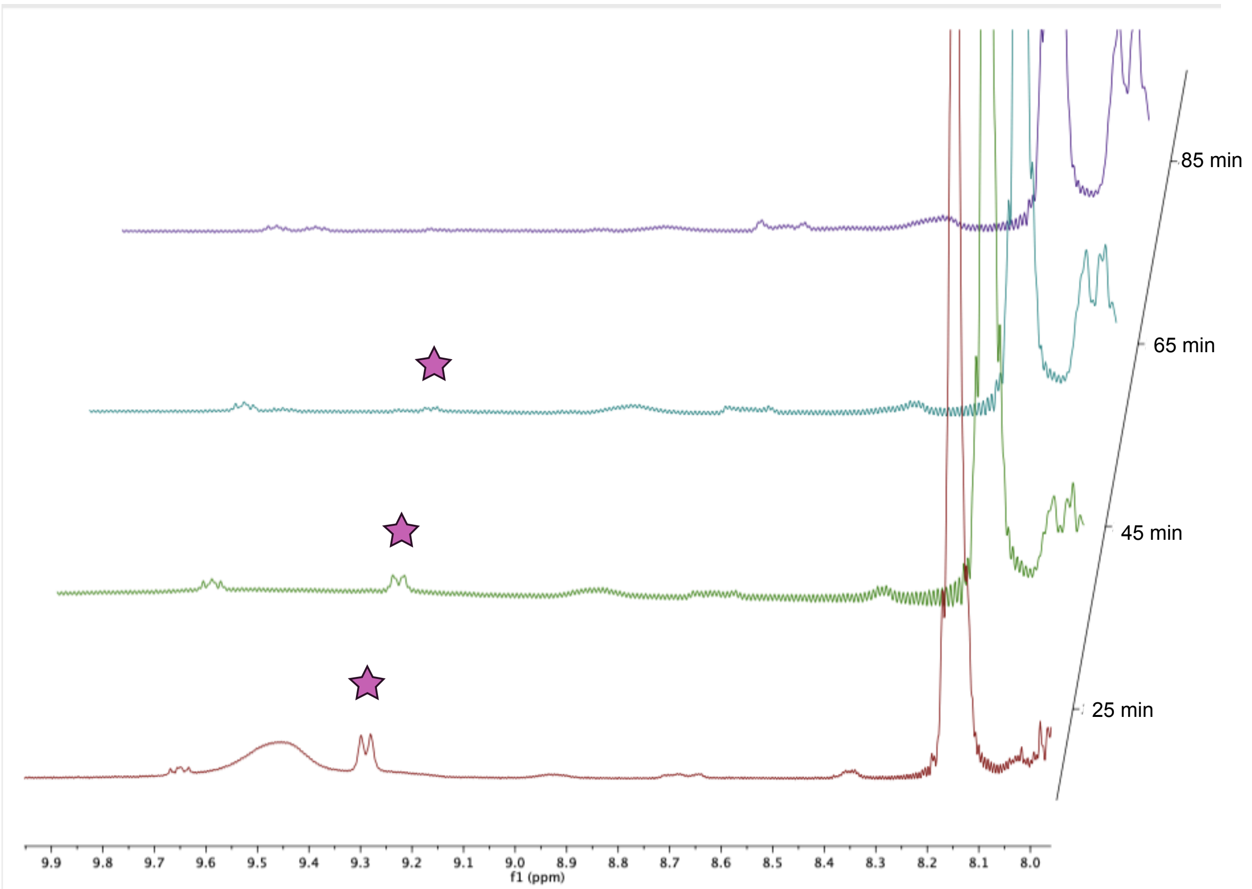

Figure S59: ^{1}H NMR time course of the reaction zoomed in on region with proposed resting state resonance at 9.3 ppm showing it decay over time.

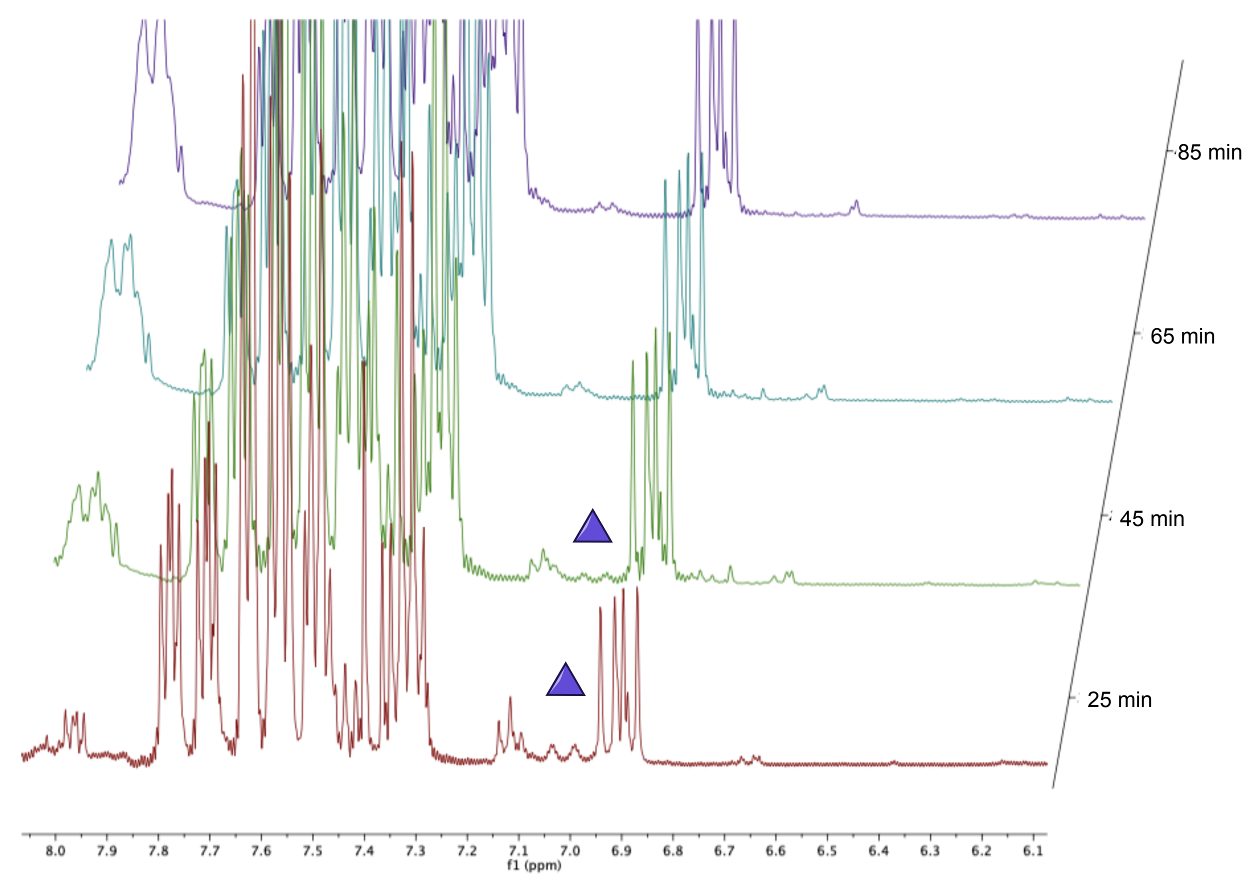

Figure S60: ^{1}H NMR time course of the reaction zoomed in on region with proposed resting state resonance at 7.05 ppm showing it decay over time.

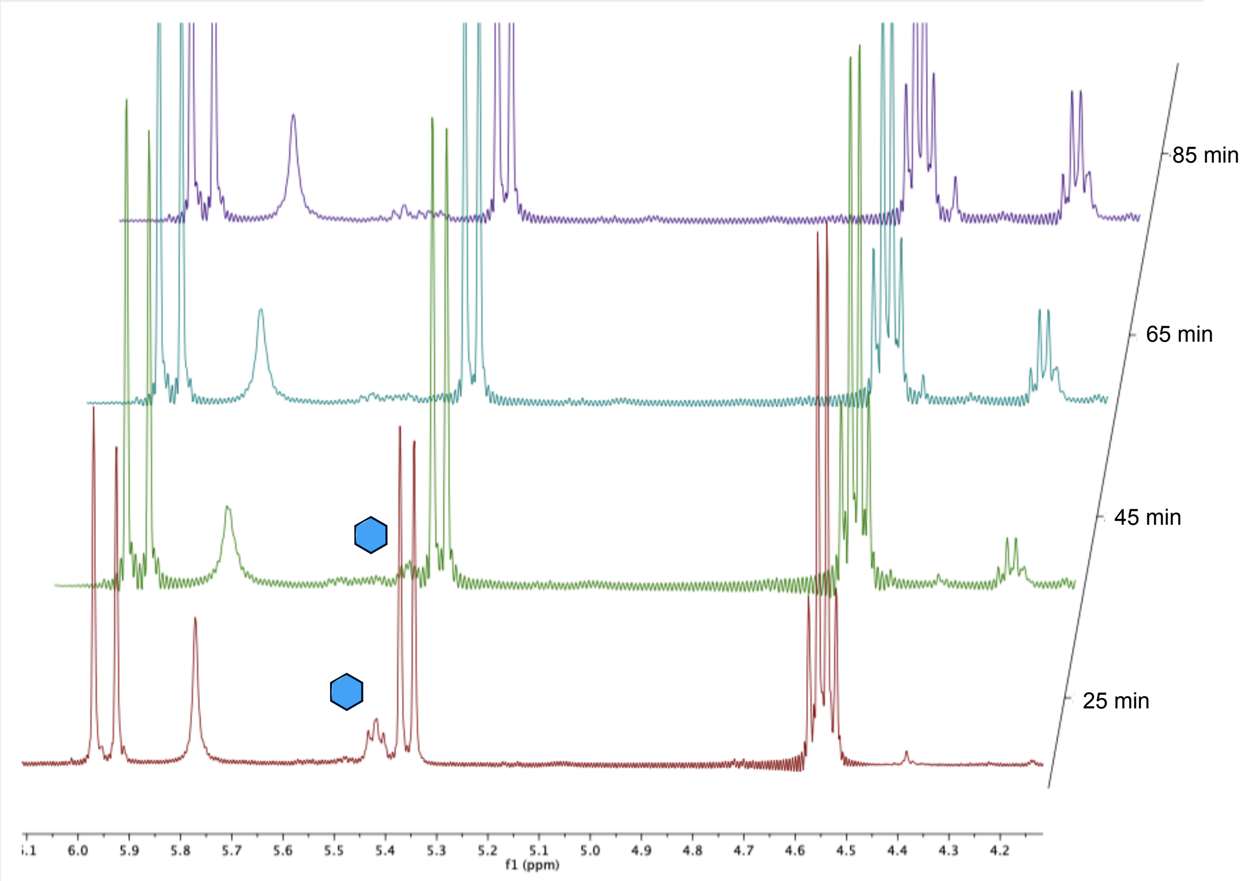

Figure S61: ^{1}H NMR time course of the reaction zoomed in on region with proposed resting state resonance at 5.4 ppm showing it decay over time.

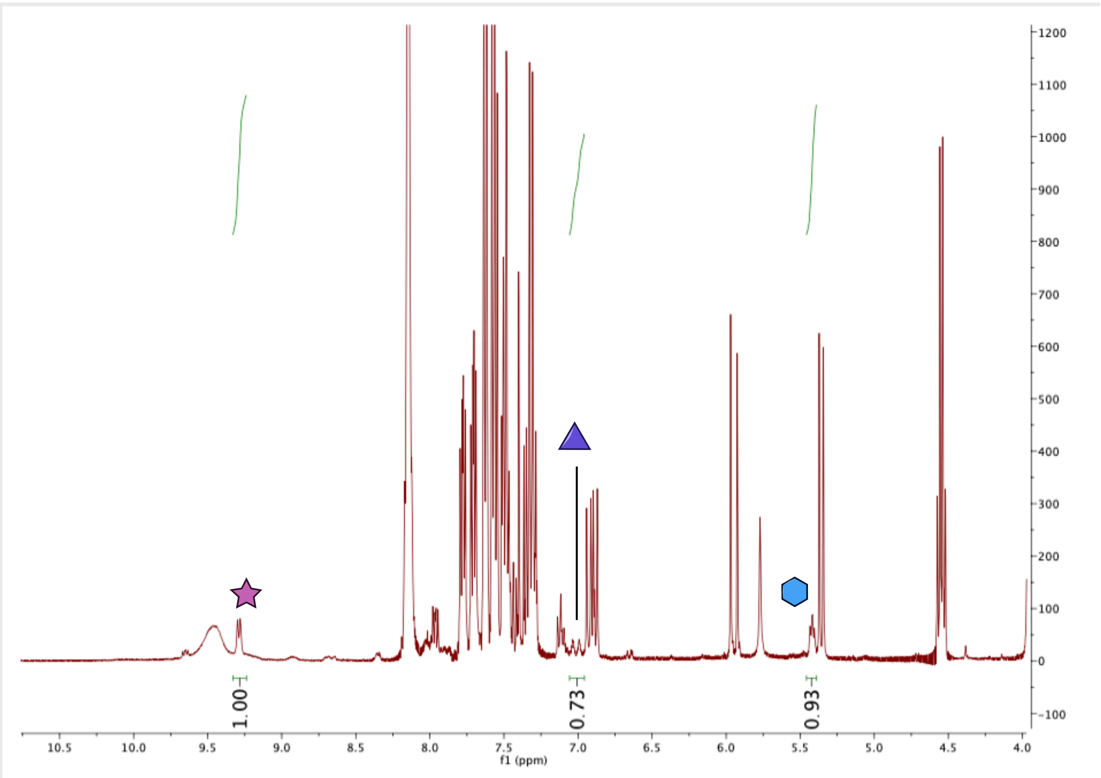

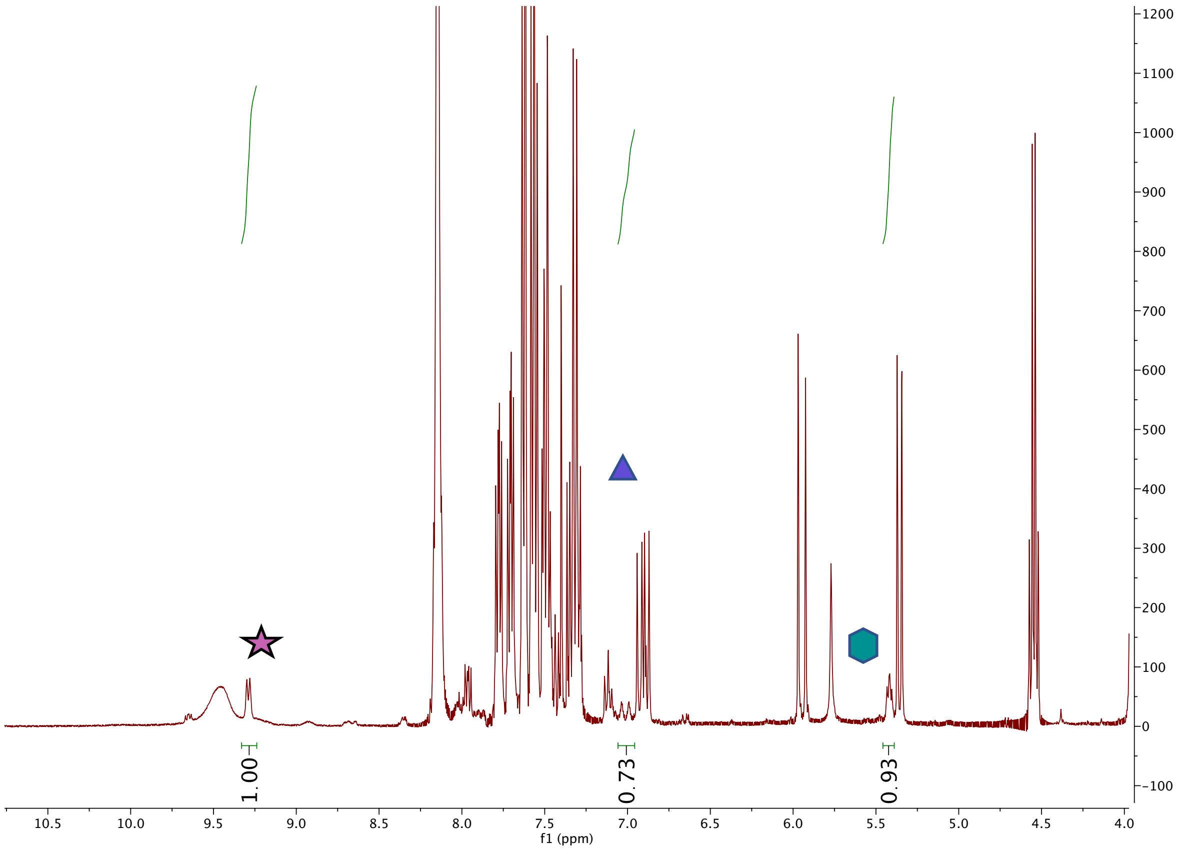

Figure S62: ^{1}H NMR at 25 minutes with proposed resting state signals integrated and highlighted.

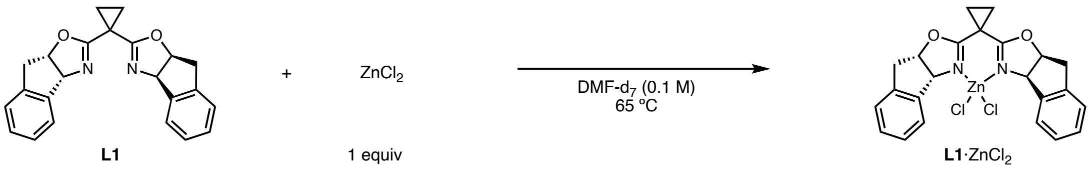

Preparation of a Diamagnetic L1·MX$_2$ ($ZnCl_2$) species for NMR Comparison: In a N_2-filled glovebox (3a*R*,3a'*R*,8a*S*,8a'*S*)-2,2'-(cyclopropane-1,1-diyl)bis(3a,8a-dihydro-8*H*-indeno[1,2-*d*]oxazole) (**L1**) (21.4 mg, 60.0 µmol, 1 equiv) was added to a dry 1 dram vial with a stir bar along with $ZnCl_2$ (8.2 mg, 60.0 µmol, 1 equiv and 600 µL) of DMF-*d*$_7$. The vial was sealed and stirred at 65 °C until homogenous, approximately 15 minutes. The contents of the vial were then added to an NMR tube, sealed with a cap and then analyzed by NMR. The change in chemical shift between free **L1** and **L1**·$ZnCl_2$ was used to see how coordination to a diamagnetic metal would change each ligand proton. ^{1}H NMR (300 MHz, DMF-*d*$_7$) δ 7.89 (dq,

J = 6.7, 1.2 Hz, 2H), 7.43 – 7.23 (m, 6H), 5.85 (dd, J = 8.2, 0.8 Hz, 2H), 5.74 (ddd, J = 8.3, 6.9, 1.7 Hz, 2H), 3.64 – 3.44 (m, 2H), 3.34 (dd, J = 18.2, 1.7 Hz, 2H), 2.12 – 1.84 (m, 4H).

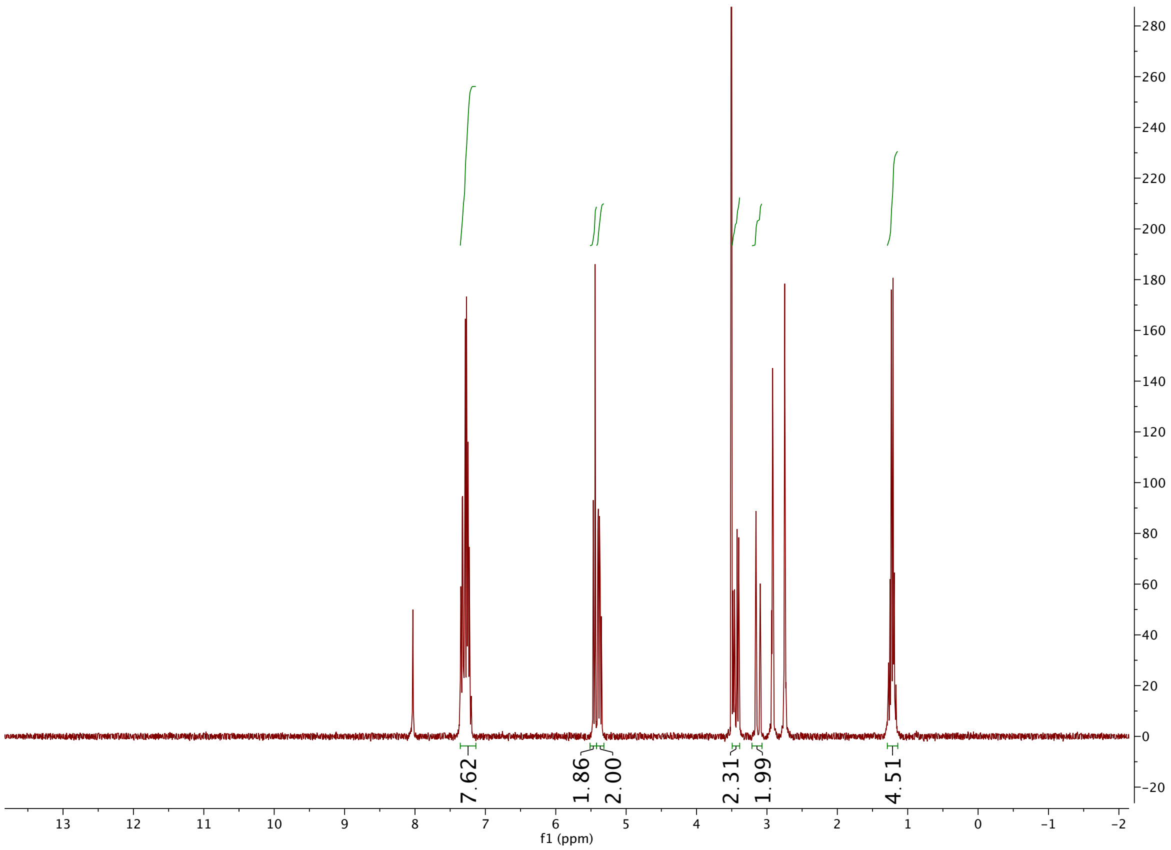

Figure S63: ^{1}H NMR of **L1** in DMF-d$_7$.

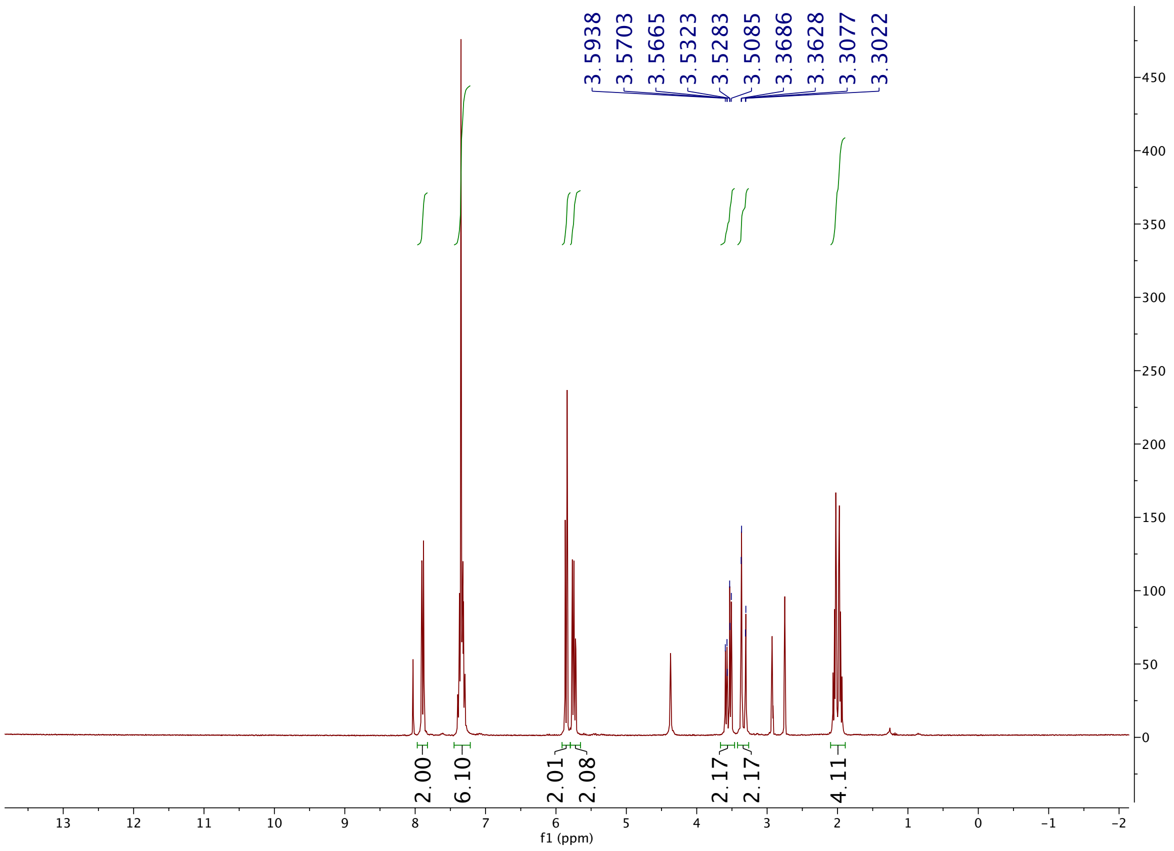

Figure S64: ^{1}H NMR of **L1**·$ZnCl_2$ in DMF-d_7.

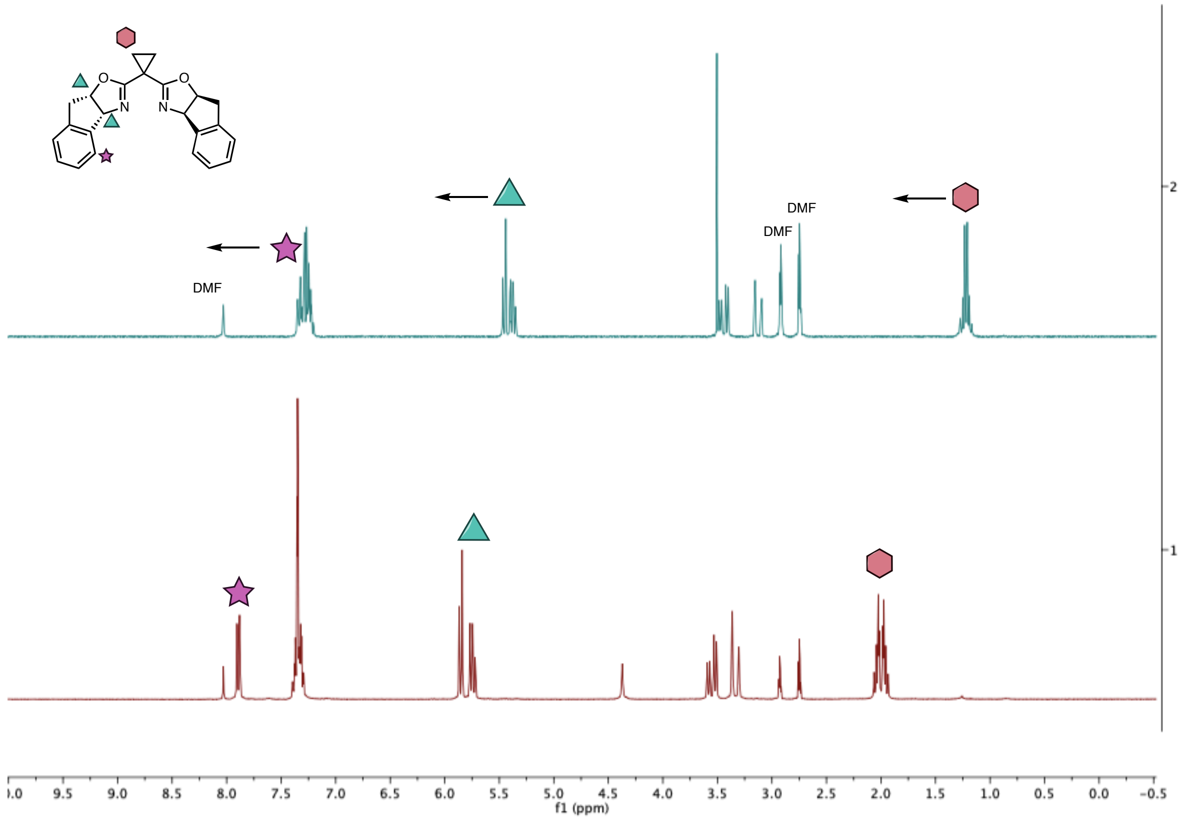

Figure S65: Comparison of **L1** (top) and **L1**·$ZnCl_2$ (bottom) to demonstrate deshielding of certain signals upon coordination to a diamagnetic metal center.

7. Electron Paramagnetic Resonance (EPR) Experiments

7.1. Generation of Ni(I) from Chemical Reduction of L1·NiX_2

Reduction of L1·$NiBr_2$ by TDAE: To an oven-dried 20 mL was added **L1**·$NiBr_2$ (5.7 mg, 0.010 mmol, 1 equiv). The vial was brought into a N_2-filled glovebox and the solid was dissolved in 10 mL of anhydrous DMA. To this vial TDAE (2.33 uL, 0.010 mmol, 1 equiv) was added. The reaction was stirred for 2 h, after which an aliquot was removed by a syringe, filtered, and transferred to an oven-dried EPR tube. The tube was sealed, removed from the glovebox, and then frozen in LN_2 for analysis by EPR at 77 K.

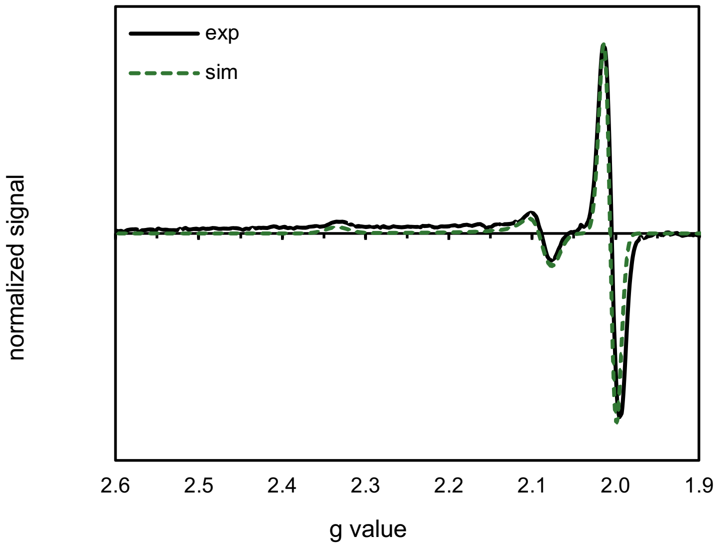

Figure S66: Observed and simulated spectra of reduction of **L1**·$NiBr_2$ by TDAE. The EPR spectra were collected at 9.371 GHz with a micropower of 2 mW, a modulation amplitude of 4 G, and a conversion time of 40.96 ms. The spectrum shown here was averaged over 4 scans. Nickel signals were simulated with g = [2.078, 2.089, 2.335] with a linewidth of 4 mT. $TDAE^{\bullet+}$ signals were simulated with g_{iso} = 2.0069 with a linewidth of 3 mT. The two species exist in a 1:0.78 ratio.

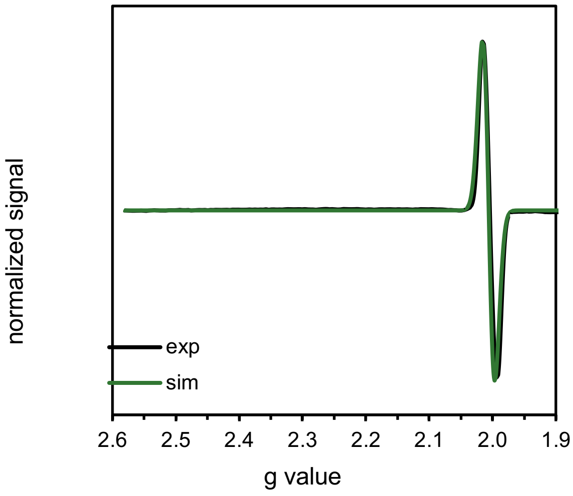

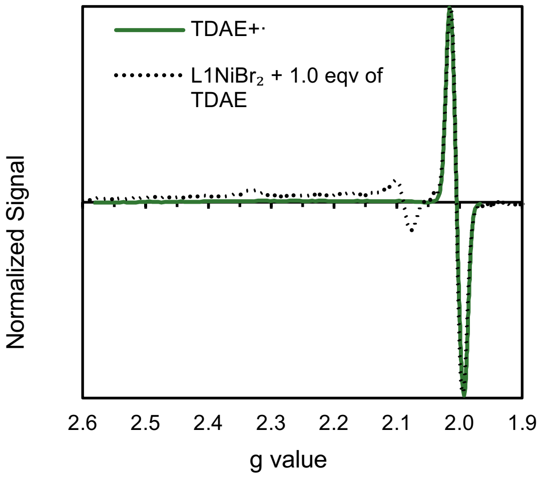

Figure S67: Observed and simulated spectra of [TDAE]Br (left). Overlay of independent [TDAE]Br and **L1**·$NiBr_2$ from Figure S66 (right). The EPR spectra were collected at 9.370 GHz with a micropower of 2 mW, a modulation amplitude of 4 G, and a conversion time of 40.96 ms. The spectrum shown here was averaged over 4 scans. The isotropic signal was simulated with g_{iso} = 2.006 with a linewidth of 3.8 mT prepared by mixing TDAE and isolated

Reduction of L1·$NiBr_2$ by L1·Ni(cod): In a N_2-filled glovebox Ni(cod)$_2$ (8.3 mg, 0.030 mmol, 1 equiv) and **L1** (10.7 mg, 0.030 mmol, 1 equiv) were added to an oven-dried 20 mL scintillation and dissolved in 7.5 mL of anhydrous DMA. The reaction was stirred for 1.5 h for and then **L1**·$NiBr_2$ (17.2 mg, 0.030 mmol, 1 equiv) was added. After 1 min of stirring, a 300 uL aliquot was removed by a syringe and transferred to an oven-dried EPR tube. The tube was sealed, removed from the glovebox, and then frozen in LN_2 for analysis by EPR at 77 K.

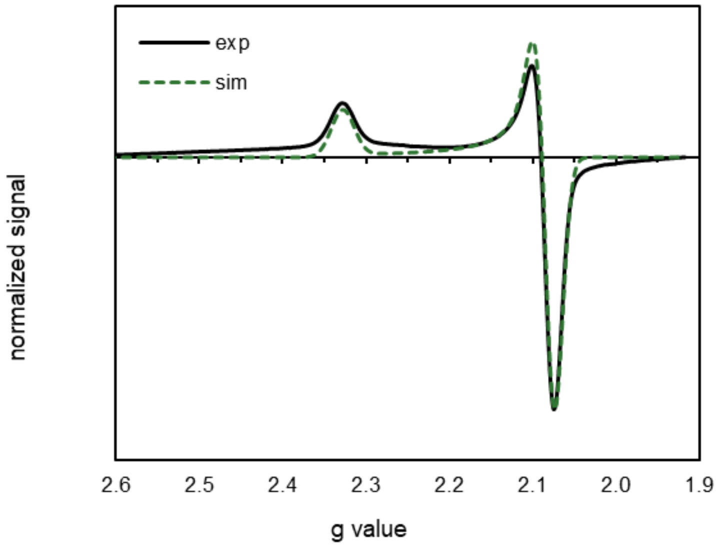

Figure S68: Observed and simulated spectra of reduction of **L1**·$NiBr_2$ by **L1**·Ni(cod). The EPR spectra were collected at 9.392 GHz with a micropower of 2.0 mW, a modulation amplitude of 4 G, and a conversion time of 4 ms. The spectrum shown here was averaged over 9 scans. Nickel signals were simulated with g = [2.075, 2.085, 2.328] with a linewidth of 4 mT.

Reduction of L1·$NiCl_2$ by Zn: For preparation procedure see S7.3

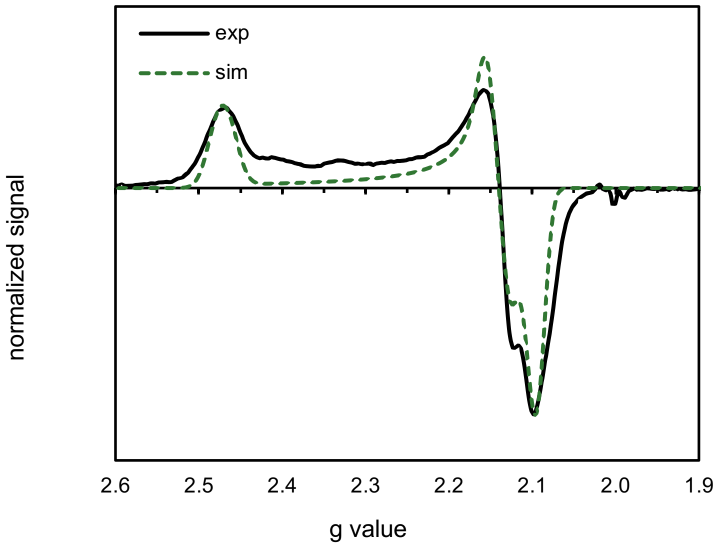

Figure S69: Observed and simulated spectra of reduction of **L1**·$NiCl_2$ by Zn at 2.75 h. The EPR spectra were collected at 9.368 GHz with a micropower 2.0 mW, a modulation amplitude of 4 G, and a conversion time of 40.96 ms. The spectrum shown here was averaged over 9 scans. The nickel signals were simulated with g = [2.095, 2.141, 2.471] with a nitrogen superhyperfine tensor A = [30, 40, 40] MHz and a linewidth of 4 mT.

7.2. Reaction of L1·Ni(cod) with 1a

reagent mixture	solvent	time	Ni species 1				Ni species 2			
			g1	g2	g3	weight	g1	g2	g3	weight
1 equiv **L1**Ni(cod) + **1a**	DMA	1 min	2.078	2.085	2.330	100%		unobserved		
2 equiv **L1**Ni(cod) + **1a**	DMA	1 min	2.078	2.085	2.330	100%		unobserved		

Table S24: Summary of EPR data of **L1**Ni·(cod) and **1a** reaction.

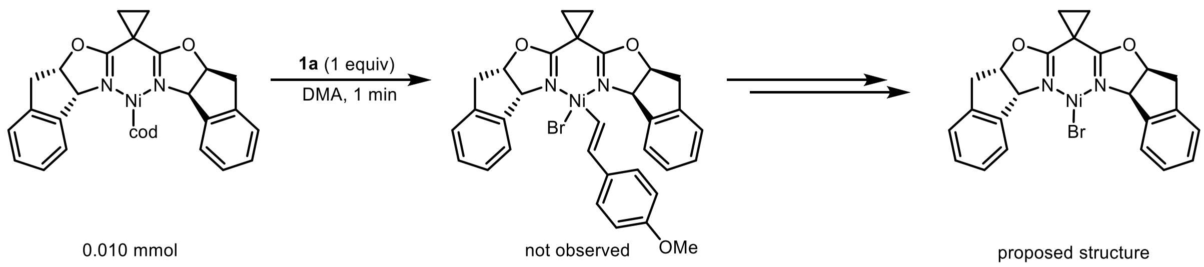

Oxidative addition of 1a by L1·Ni(cod): In a N_2-filled glovebox $Ni(cod)_2$ (2.8 mg, 0.010 mmol, 1 equiv) and **L1** (3.7 mg, 0.010 mmol, 1 equiv) were added to an oven-dried 2-dram vial. The mixture was dissolved in DMA and stirred for 24 h at 30 °C, after which **1a** (2.1 mg, 0.100 mmol, 1 equiv) was added . In a separate vial, 21.4 mg of **1a** was dissolved in 100 uL of anhydrous DMA; 10 uL (0.010 mol, 1 equiv) or 5 uL (0.005 mol, 0.5 equiv) of this 1M stock solution was added **L1**·Ni(cod). The reaction was stirred for 1 min after which an aliquot was

removed and transferred to an oven-dried EPR tube. The tube was sealed, removed from the glovebox, and then frozen in LN_2 for analysis by EPR at 77 K.

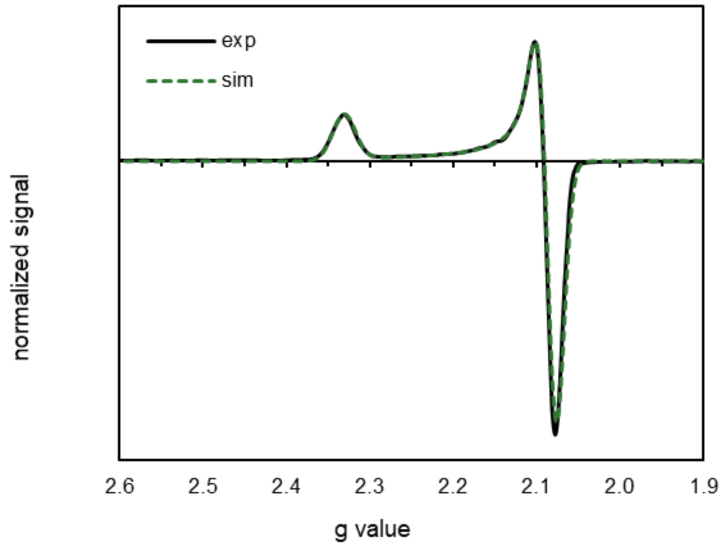

Figure S70: Truncated observed and simulated spectra of the stoichiometric mixture of **L1**·Ni(cod) (1.0 equivalent) and **1a** (1.0 equivalent). The EPR spectrum was collected at 9.511 GHz with a micropower of 6.4 mW, a modulation amplitude of 4 G, and a conversion time of 40.96 ms. No additional signals were observed for g values between 2.60 and 6.80 that would suggest S ≠ ½ species were present. The nickel signals were simulated with g = [2.078, 2.085, 2.330] with a linewidth of 4 mT.

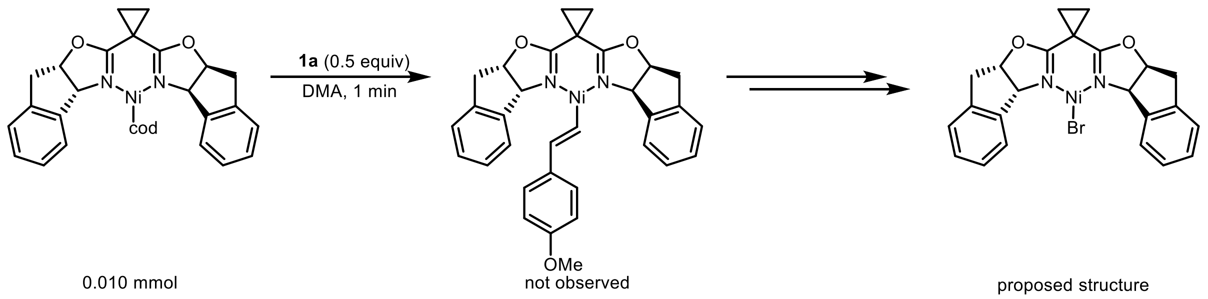

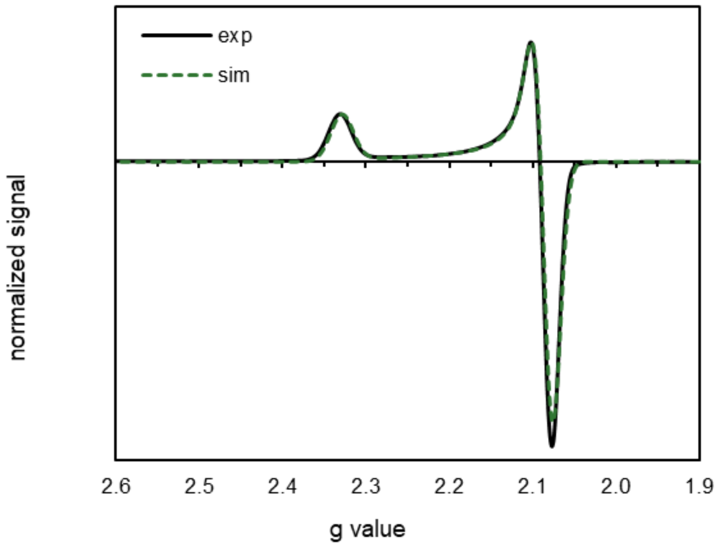

Figure S71: Truncated observed and simulated spectra of the stoichiometric mixture of **L1**Ni(cod) (2.0 equivalent) and **1a** (1.0 equivalent). The EPR spectrum was collected at 9.510 GHz with a micropower of 6.4 mW, a modulation amplitude of 4 G, and a conversion time of 40.96 ms. No additional signals were observed for g values between 2.60 and 6.79 that would suggest S ≠ ½ species were present. The nickel signals were simulated with g = [2.078, 2.085, 2.328] with a linewidth of 4 mT.

7.3. Time Course Reduction of L1·$NiCl_2$ with Zn^0

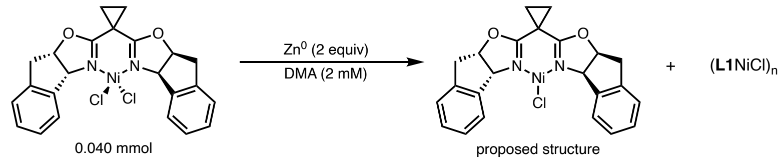

Reduction Reaction and Sample Preparation Procedure: To an oven-dried 50 mL round bottom flask was added Zn^0 powder (5.2 mg, 0.080 mmol, 2 equiv) and **L1**·$NiCl_2$ (19.4 mg, 0.040 mmol, 1 equiv). The flask was then brought into a N_2-filled glovebox where DMA (20 mL, 2 mM) was added. Before the reaction was stirred, a 2 mL aliquot was removed by a syringe fit with a 0.45 μm filter and added to a 10 mm pathlength optical cell and an oven-dried EPR tube. The tube was then removed from the glovebox and frozen in LN_2 for analysis by perpendicular mode EPR at 77 K and parallel mode EPR at 5 K. The optical cell was sealed and removed from the glovebox for analysis by optical spectroscopy. The reaction mixture was then stirred at 1000 rpm to start the reaction and aliquots (same procedure as before) were removed at regular intervals: 0.5 h, 1 h, 1.5 h, 2.75 h, 5 h, 8 h, and 30 h.

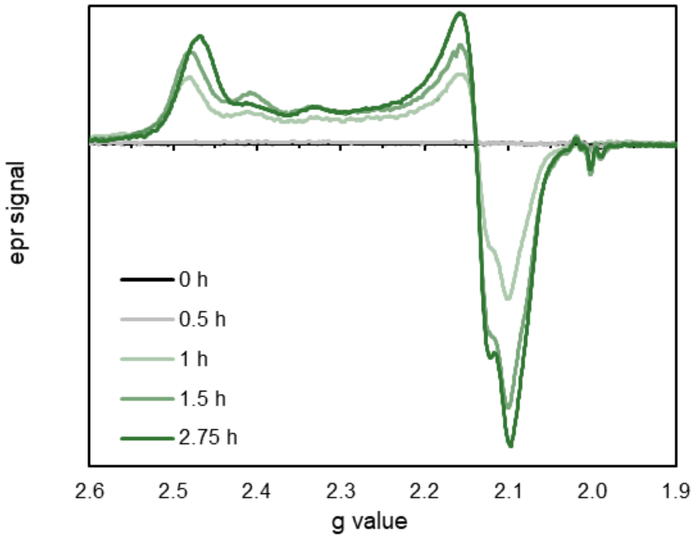

Figure S72: Observed perpendicular mode EPR spectra during the first 2.75 h of reaction. The EPR spectra were taken at 9.368 GHz (0 h), 9.364 GHz (0.5 h), 9.375 GHz (1 h), 9.368 GHz (1.5 h), and 9.368 GHz (2.75 h). Spectrum at each timepoint was averaged over 9 scans. The spectra were collected at microwave power of 2.0 mW with a modulation amplitude of 4 G and conversion time of 40.96 ms.

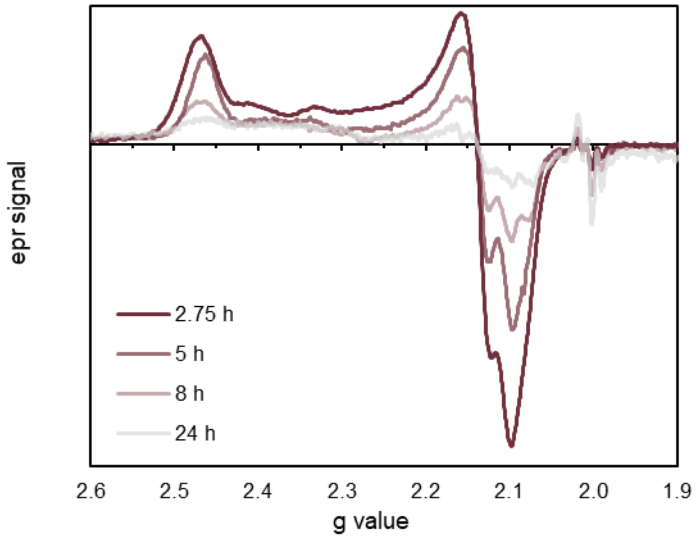

Figure S73: Observed perpendicular mode EPR spectra after 2.75 h of the reaction. The EPR spectra were taken at 9.368 GHz (2.75 h), 9.369 GHz(5 h), 9.367 GHz(8 h), and 9.367 GHz (24 h), Spectrum at each timepoint was averaged over 9 scans. The spectra were collected at microwave power of 2.0 mW with a modulation amplitude of 4 G and conversion time of 40.96 ms.

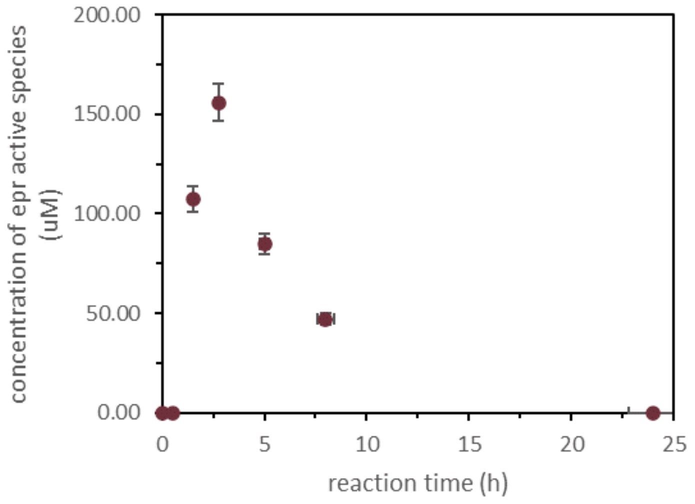

Figure S74: Concentration of EPR active species quantified with an external calibration curve. [9] The integrated EPR intensity at each timepoint were halved to account for the microwave power difference between the calibration curve (0.5 mW) and the experimental condition (2.0 mW). A 6% error bar is shown in accordance with the error analysis.

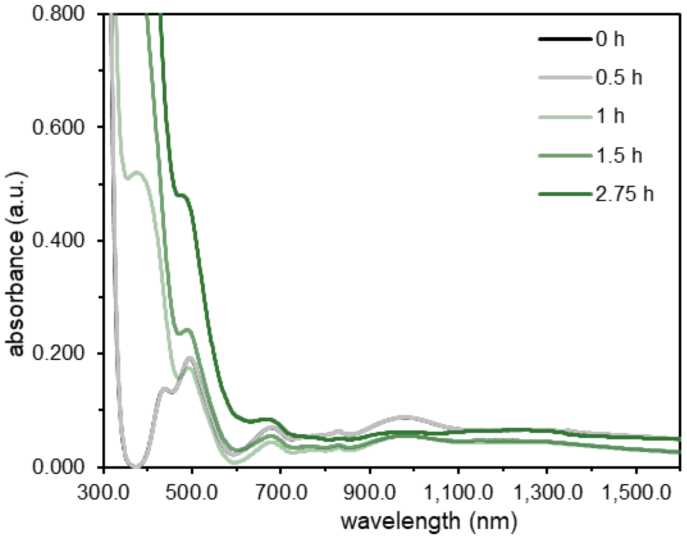

Figure S75: Corresponding optical spectra during the first 2.75 h of the reaction.

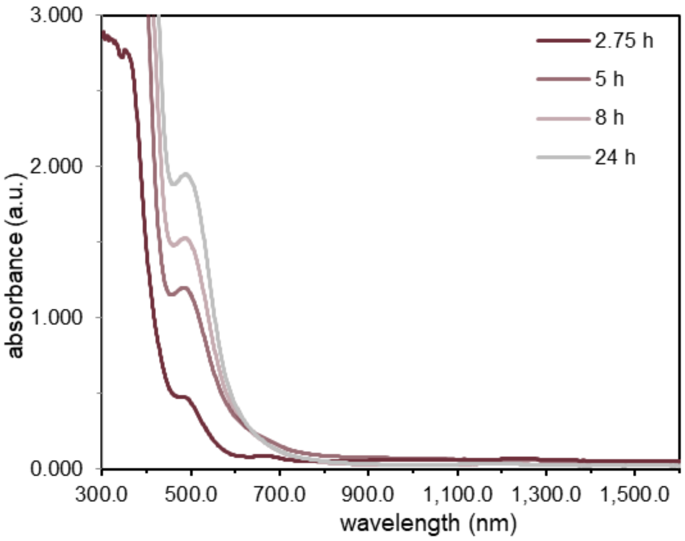

Figure S76: Corresponding optical spectra after the first 2.75 h of the reaction

7.4. Reaction Monitoring with EPR

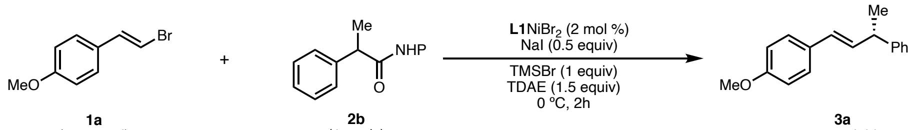

Cross-Coupling Reaction and Sample Preparation Procedure: In a dry 20 mL scintillation vial with a stir bar was added (*E*)-1-(2-bromovinyl)-4-methoxybenzene **1a** (63.9 mg, 0.30 mmol, 1 equiv) 1,3-dioxoisoindolin-2-yl 2-phenylpropanoate **2b** (88.6 mg, 0.30 mmol, 1 equiv), **L1**·$NiBr_2$ (3.5 mg, 6.0 µmol, 0.02 equiv), and NaI (22.5 mg, 0.15 mmol, 0.5 equiv). The vial was then placed under Ar, sealed and brought into a N_2-filled glovebox. The contents of the vial were dissolved in DMA (3.0 mL, 0.1 M) and cooled to 0 ºC in a cold well. Once cooled, TMSBr (39.6 µL, 0.30 mmol, 1 equiv) was added and the reaction was stirred for 5 min before TDAE (105 µL, 0.45 mmol, 1.5 equiv) was added to start the reaction. After 5 minutes, a ~0.4 mL aliquot was removed by syringe fitted with a 0.45 µm filter and transferred to an oven-dried EPR tube. The tube was then rapidly removed from the glove box and frozen in LN_2 for EPR analysis. The same procedure was done at 15 min and 90 min. After the final aliquot was removed, the reaction was quenched with 0.5 mL 1M HCl, further diluted with 3 mL H_2O then extracted three times with 5 mL of Et_2O. The combined organics were then washed with 3 mL 1M LiCl, dried over $MgSO_4$, then filtered and concentrated. The crude mixture was then diluted with 10 mL EtOAc and analyzed by GC-FID to confirm the presence of product **3a** (55% yield).

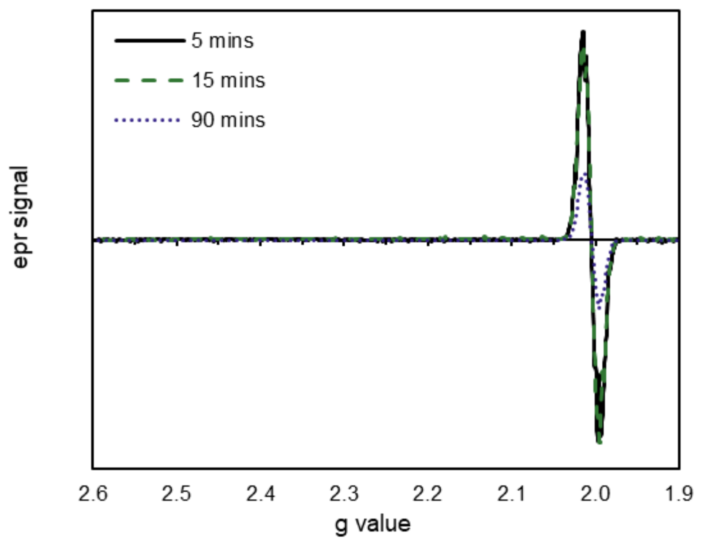

Figure S77: EPR spectra over the course of the reaction. The EPR spectra were taken at 9.360 GHz (5 mins), 9.360 GHz (15 mins), and 9.362GHz (90 mins). Spectrum at each timepoint was averaged over 4 scans. The spectra were collected at a modulation amplitude of 4 G and conversion time of 40.96 ms. The spectra were taken at 0.5 mW (shown here), 2.0 mW, and 8.0 mW, which showed EPR no oversaturation occurred for a microwave power below 2.0 mW.

8. Catalyst Loading Study (Figure 5d)

8.1. Screening Catalysts loadings with TMSBr and TESBr

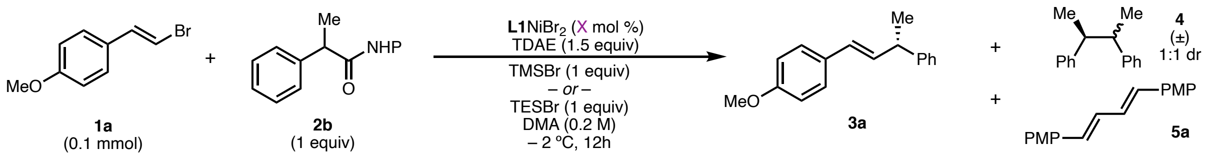

Changing Lewis Acid at Different Catalyst Loadings Cross-Coupling Procedure: In a N_2-filled glovebox to an oven-dried 20 mL scintillation vial was added **1a**, **2b**, *n*-dodecane internal standard and dry DMA to make a 0.4 M stock solution of substrates. In an oven-dried 1-dram vial with a stir bar was added **L1**$NiBr_2$ complex followed by dry DMA to make a 0.08 M catalyst stock solution. Stock solutions of substrates and catalyst were added to oven-dried 1 dram vials with a stir bar then diluted with dry DMA (final reaction concentration 0.2 M). This was done such that there was 6 different catalyst loadings: 20, 10, 5, 2.5, 1.0, 0.5 mol% and done in triplicate for each loading (total 18 reactions for each Lewis acid). Each reaction was then cooled to –2 ºC and stirred at 200 rpm. *Note: Julabo LH45 chiller was set to 0 ºC however we measured a reaction temperature of –2 ºC for these experiments.* Once cooled, either

TMSBr (13.2 μL, 0.10 mmol, 1 equiv) or TESBr (17.2 μL, 0.10 mmol, 1 equiv) was added to each reaction and they were allowed to stir for 10 minutes before TDAE (35 μL, 0.15 mmol, 1.5 equiv) was added to start the reaction. Each reaction was allowed to stir for 12 hours at –2 ºC in a N_2-filled glovebox. Upon completion the reactions were diluted with EtOAc and quenched with 1M HCl. An aliquot of the organic layer was extracted and filtered through MgSO4 then further diluted for GC analysis. For one sample reaction of each Lewis acid/catalyst loading combination the remaining crude was purified by preparative-TLC for chiral SFC analysis to determine ee.

Data analysis: Reaction yields and conversions were determined by GC-FID analysis against *n*-dodecane internal standard. In Figure 5d, Figure S78, and Figure S79 the average of three runs is displayed with the error bars representing the standard deviation of the three runs. Yields of homocoupled products **4** and **5a** are not based on their theoretical yields (0.05 mmol) but instead of the reaction product's theoretical yield (0.10 mmol) to allow for facile comparison of relative mass balance across reactions.

Raw Data:

run #	Ni mol %	**3a** yield (%)	**4** yield (%)	**5** yield (%)
1	20	84	3	0
2	20	84	3	0
3	20	85	3	0
1	10	85	4	0
2	10	84	5	0
3	10	85	4	0
1	5.0	87	4	0
2	5.0	88	4	0
3	5.0	88	4	0
1	2.5	93	3	0
2	2.5	93	3	0
3	2.5	94	3	0
1	1.0	87	5	1
2	1.0	88	5	0
3	1.0	89	5	0
1	0.5	72	14	0
2	0.5	72	14	0
3	0.5	71	14	0

Table S25: Raw data on reactions using TMSBr as a Lewis acid at varying catalyst concentrations.

run #	Ni mol %	**3a** yield (%)	**4** yield (%)	**5** yield (%)
1	20	73	8	13
2	20	74	8	15
3	20	71	8	13
1	10	75	8	12
2	10	75	9	13
3	10	73	9	10
1	5.0	80	7	9
2	5.0	83	7	1
3	5.0	85	7	11
1	2.5	87	5	8
2	2.5	88	5	11
3	2.5	89	5	9
1	1.0	92	3	11
2	1.0	95	3	9
3	1.0	96	3	8
1	0.5	82	9	8
2	0.5	87	3	9
3	0.5	84	9	8

Table S26: Raw data on reactions using TESBr as a Lewis acid at varying catalyst concentrations.

	Ni mol %	**3a** avg yield ± STDEV	**4** avg yield ± STDEV	**5a** avg yield ± STDEV	**3a** ee (%)
TMSBr	20	84 ± 0.4	3 ± 0	0 ± 0	96
	10	85 ± 0.6	4 ± 0.2	1 ± 0	95
	5	88 ± 0.6	4 ± 0	2 ± 0	93
	2.5	93 ± 0.8	3 ± 0	3 ± 0	88
	1	88 ± 0.8	5 ± 0	4 ± 0	82
	0.5	72 ± 0.6	14 ± 0.2	5 ± 0	76
TESBr	20	73 ± 1.2	8 ± 0	14 ± 1.2	95
	10	74 ± 1.4	9 ± 0.2	12 ± 1.2	94
	5	82 ± 2.4	7 ± 0	7 ± 5.4	93
	2.5	88 ± 0.8	5 ± 0	9 ± 1.2	90
	1	94 ± 2.0	3 ± 0	9 ± 1.4	85
	0.5	84 ± 2.6	7 ± 3.6	58± 0.6	78

Table S27: Averaged yield data with standard deviation for each catalyst loading and Lewis Acid. Yields determined by GC-FID analysis versus dodecane internal standard.

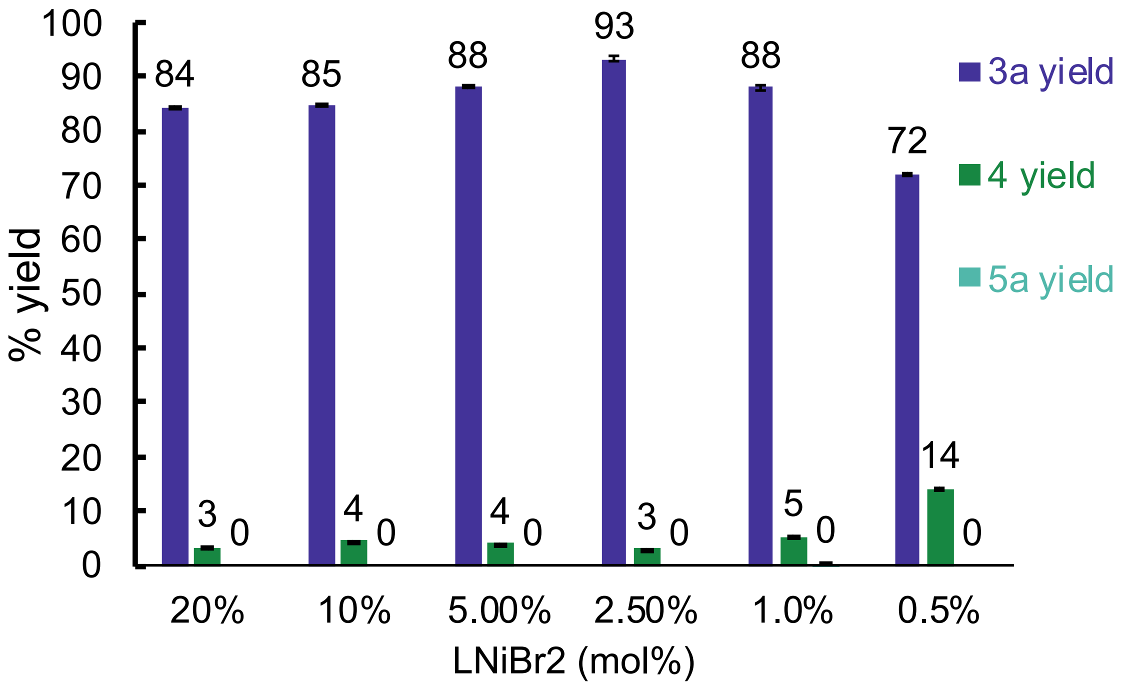

Figure S78: TMSBr reaction data in graphical form similar to main text (Figure 5d).

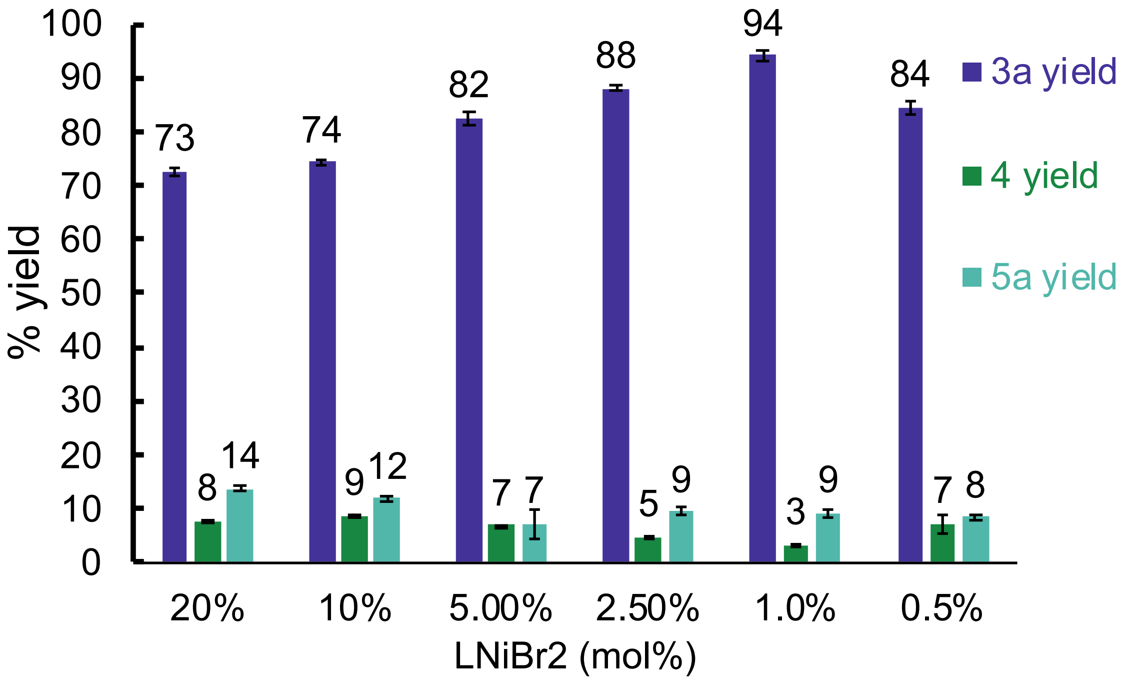

Figure S79: TESBr reaction data in graphical form similar to main text (Figure 5d).

8.2. Scale Up of Optimal TESBr conditions:

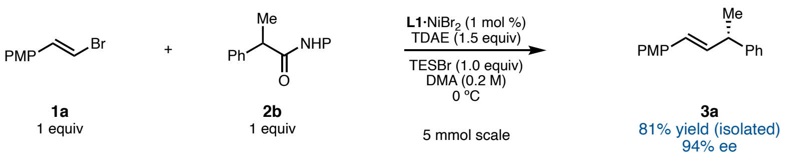

Procedure for 5 mmol scale coupling: An oven-dried 100 mL round bottom flask with a stir bar was cooled under N_2 before (*E*)-1-(2-bromovinyl)-4-methoxybenzene (**1a**, 1.07g, 5.0 mmol, 1 equiv), 1,3-dioxoisoindolin-2-yl 2-phenylpropanoate (**2b**, 1.48g, 5.0 mmol, 1 equiv), and **L1**·$NiBr_2$ (28.7 mg, 0.05 mmol, 0.01 equiv) were added. The flask was then sealed with a septa and inlet needle was inserted. The flask was then evacuated and backfilled with N_2 three times before 25 mL of dry DMA (0.2 M) were added via syringe. The flask was then submerged in an ice bath and the contents were stirred under N_2 as the reaction cooled. To the flask was then added bromotriethylsilane (860 μL, 5 mmol, 1 equiv, measured out in an N_2-filled glovebox) dropwise over the course of 5 minutes. The reaction was then stirred at 0 ºC for 15 min before tetrakis(dimethylamino)ethyene (1.74 mL, 7.5 mmol, 1.5 equiv, measured out in an N_2-filled glovebox) was added dropwise over the course of 5 minutes causing the red solution to turn dark. The reaction was allowed to stir under N_2 at 0 ºC for 15 hours before it was quenched with 75 mL of 1M HCl (*caution: exothermic!*) at 0 ºC then allowed to warm to ambient temperature. The crude was then extracted 3x with 75 mL EtOAc and combined organics were dried over $MgSO_4$, filtered through celite, and concentrated *in vacuo*. The crude was then purified with column chromatography (SiO_2, 75 g) using a solvent gradient of 2% –> 20% PhMe in hexanes to give product **3a** (965 mg, 4.1 mmol, 81% yield) as a colorless oil. The ee was determined to be 94% by chiral SFC analysis of purified material.

9. Computational Data

Unless otherwise noted, all calculations were carried out with the Gaussian 16 package.[10] Geometry optimization and energy calculations were performed with B3LYP-D3.[11] The LANL2DZ basis set[12] with ECP was used for Ni, and the 6-31G(d) basis set[13] was used for other atoms. Frequency analysis was conducted at the same level of theory to verify that the stationary points are minima or saddle points. To ensure that the correct unrestricted wave functions were obtained, a stability test was carried out with the Gaussian keyword *stable = opt*. Single point energies were calculated at the M06[14]/6-311+G(d,p)-SDD[15] level using SMD solvation model[16] (solvent = DMA). Computed structures were visualized using CYLview.[17]

[19]F Chemical shifts were carried out with ORCA 4.2.1 package. [18] Geometry optimizations and frequency calculations were performed with BP86. Chemical shifts calculation were performed with TPSSh and accelerated with RIJCOSX approximation using SMD solvation model (solvent = DMA). All atoms were described with def2-TZVPP basis set.

9.1. DFT-Computed Gibbs Free Energy Barriers for Radical Addition and Reductive Elimination Transition States

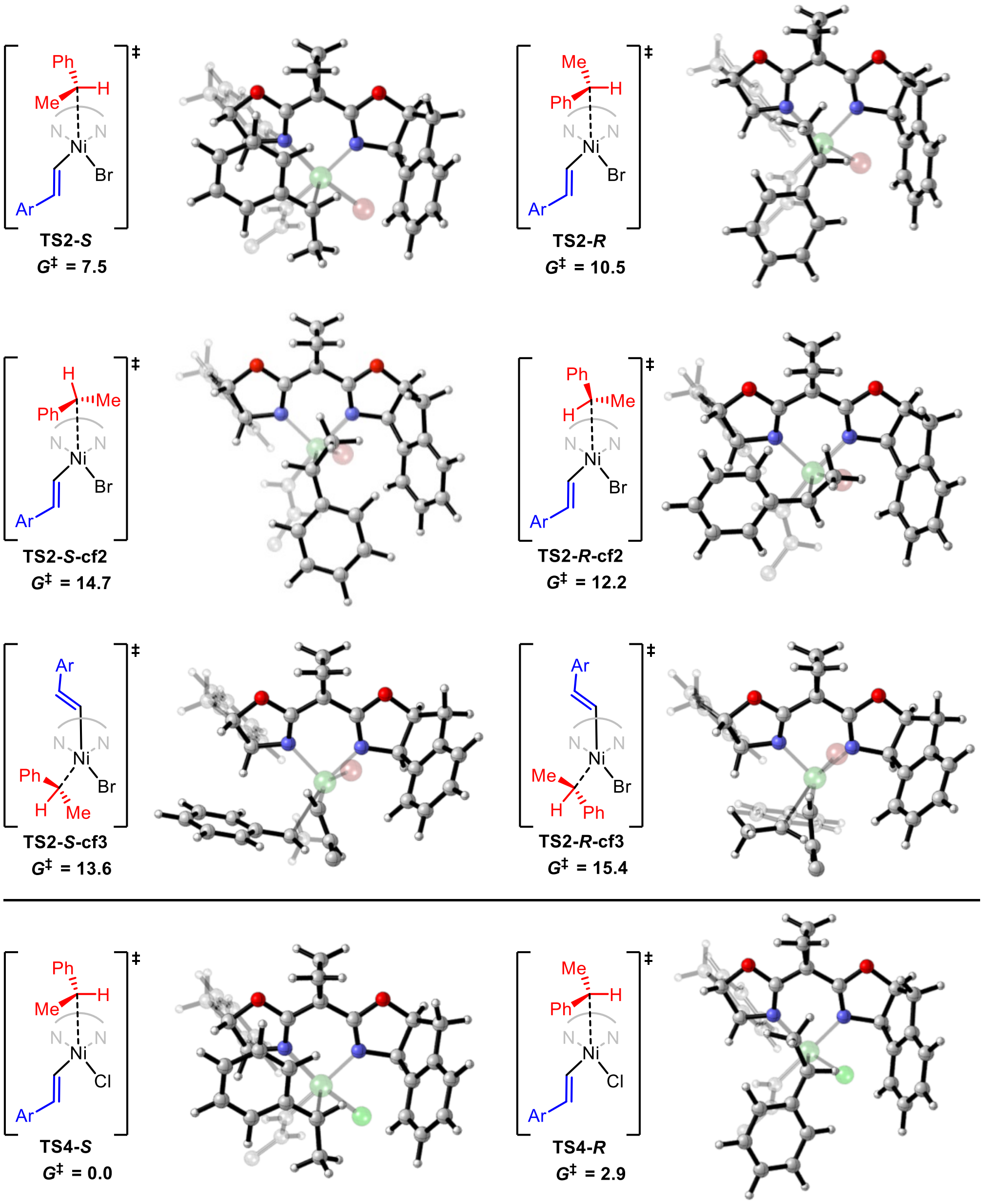

Figure S80. DFT-computed Gibbs free energy barriers for radical addition transition states. Energies in kcal/mol, only the α-carbon of aryl group (*p*-OMe-Ph) is shown for simplicity.

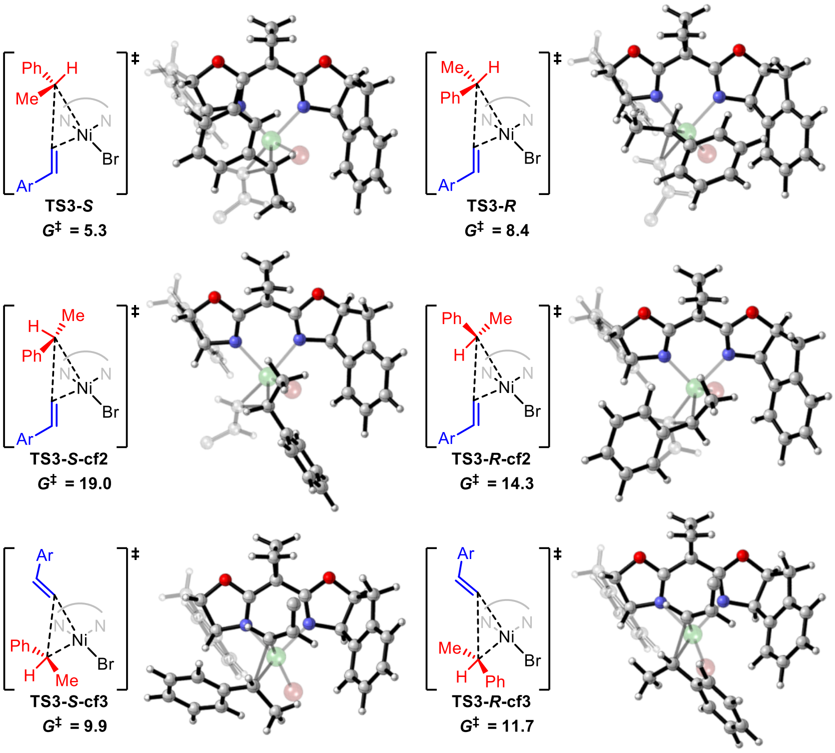

Figure S81. DFT-computed Gibbs free energy barriers for reductive elimination transition states. Energies in kcal/mol, only the α-carbon of aryl group (*p*-OMe-Ph) is shown for simplicity.

9.2. Table of Energies

structures	*ZPE*	*TCH*	*TCG*	*E*	*H*	*G*	Imaginary Frequency
**1a**	0.157103	0.168714	0.118363	-2994.814718	-2994.813774	-2994.864125	—
**10**	0.143410	0.151702	0.111157	-310.086145	-310.085200	-310.125745	—
**3a**	0.305310	0.322803	0.258415	-733.546167	-733.545222	-733.609611	—
**6-Br**	0.387446	0.412098	0.330548	-3888.876796	-3888.875852	-3888.957402	—
**9**	0.545058	0.582077	0.471467	-6883.698127	-6883.697183	-6883.807793	—
**7**	0.544906	0.579268	0.475840	-4312.265954	-4312.265010	-4312.368438	—
**11**	0.693066	0.735906	0.613379	-4622.384353	-4622.383409	-4622.505936	—
**L1·NiBr$_2$**	0.389305	0.415738	0.330948	-6460.314111	-6460.313167	-6460.397957	—
**L1·NiCl$_2$**	0.390313	0.416097	0.334385	-2237.879255	-2237.878311	-2237.960023	—
**TS1**	0.544407	0.580802	0.470800	-6883.686941	-6883.685997	-6883.795998	77.97*i*
**TS2-*S***	0.691161	0.733509	0.612775	-4622.377688	-4622.376744	-4622.497478	86.29*i*
**TS2-*S*-cf2**	0.690994	0.733306	0.613450	-4622.366077	-4622.365133	-4622.484988	99.49*i*
**TS2-*S*-cf3**	0.689948	0.732831	0.609722	-4622.368744	-4622.367800	-4622.490909	43.26*i*
**TS2-*R***	0.690489	0.733056	0.610999	-4622.369068	-4622.368124	-4622.490181	93.39*i*

**TS2-*R*-cf2**	0.691202	0.733535	0.613484	-4622.373867	-4622.372923	-4622.492974	122.69*i*
**TS2-*R*-cf3**	0.690101	0.732833	0.611372	-4622.363507	-4622.362563	-4622.484024	29.39*i*
**TS3-*S***	0.692081	0.734347	0.612426	-4622.378747	-4622.377802	-4622.499724	228.06*i*
**TS3-*S*-cf2**	0.693128	0.735204	0.613722	-4622.360576	-4622.359631	-4622.481113	188.10*i*
**TS3-*S*-cf3**	0.692833	0.734938	0.616204	-4622.384026	-4622.383082	-4622.501816	163.77*i*
**TS3-*R***	0.692106	0.734445	0.612125	-4622.370255	-4622.369311	-4622.491631	250.12*i*
**TS3-*R*-cf2**	0.693082	0.735002	0.614428	-4622.367556	-4622.366612	-4622.487187	213.19*i*
**TS3-*R*-cf3**	0.692876	0.734861	0.616167	-4622.379267	-4622.378323	-4622.497017	195.93*i*
**TS4-*S***	0.691521	0.733615	0.613914	-2511.159973	-2511.159029	-2511.278730	91.24*i*
**TS4-*R***	0.690833	0.733155	0.611956	-2511.150744	-2511.149799	-2511.270998	102.88*i*

Table S28. Zero-point correction (ZPE), thermal correction to enthalpy (TCH), thermal correction to Gibbs free energy (TCG), energies (E), enthalpies (H), and Gibbs free energies (G) (in Hartree) of the structures calculated at the M06/6-311+G(d,p)-SDD-SMD(DMA)//B3LYP-D3/6-31G*-LANL2DZ level of theory.

9.3. Cartesian Coordinates for Calculated Species

1a

```
Br      3.88090800   -0.05446600   -0.00003200
C       2.04318100    0.44922200    0.00055600
C       1.05264300   -0.45014700   -0.00077900
H       1.91071500    1.52387800    0.00210800
H       1.32134700   -1.50469200   -0.00207000
C      -0.38593400   -0.16251700   -0.00053200
C      -1.29087000   -1.23408600   -0.00023400
C      -0.92482900    1.14221900   -0.00060600
C      -2.67281500   -1.03722800    0.00012800
H      -0.90780400   -2.25189200   -0.00022900
C      -2.29264800    1.35456200   -0.00024800
H      -0.26441000    2.00430200   -0.00106700
C      -3.18250600    0.26592200    0.00014900
H      -3.33165300   -1.89772700    0.00037100
H      -2.70717900    2.35770000   -0.00033600
O      -4.50597500    0.58767300    0.00046800
C      -5.45497000   -0.46821100    0.00085400
H      -6.43595400    0.01042300    0.00109800
H      -5.35854200   -1.09783700   -0.89418800
H      -5.35801300   -1.09766100    0.89596200
```

10

```
C       1.83322100   -0.65199900    0.00004500
H       2.08025400   -1.71111700    0.00002500
C       0.46218600   -0.30019600    0.00000800
C      -0.54340000   -1.31146300   -0.00004900
C       0.02037200    1.05542000    0.00002500
C      -1.89146700   -0.98836800   -0.00008600
H      -0.23452900   -2.35444100   -0.00006300
C      -1.33233400    1.36765200   -0.00001200
H       0.75473700    1.85543900    0.00006800
```

C	-2.30004300	0.35380700	-0.00006800
H	-2.63543400	-1.78104500	-0.00012900
H	-1.64206100	2.40984800	0.00000200
H	-3.35692000	0.60491000	-0.00009600
C	2.95955100	0.33474300	0.00010600
H	2.92767900	0.99432600	0.88074100
H	3.92999900	-0.16984900	0.00014200
H	2.92775500	0.99434700	-0.88051600

3a

C	-0.89419600	1.51166900	-0.25111200
C	-0.15813300	0.39239600	-0.18067500
H	-0.39834100	2.48271600	-0.27730500
H	-0.67988500	-0.56313700	-0.16658600
C	1.30641100	0.29100500	-0.12049800
C	1.90139600	-0.97895400	-0.09917500
C	2.17066300	1.40603900	-0.07970400
C	3.28615800	-1.15298300	-0.04467500
H	1.26489100	-1.86058800	-0.12772600
C	3.54627000	1.25264500	-0.02497100
H	1.75879600	2.41092300	-0.08770300
C	4.11939100	-0.03019900	-0.00767100
H	3.69536400	-2.15666900	-0.03153000
H	4.20849300	2.11234000	0.00722700
O	5.48282900	-0.06751800	0.04718400
C	6.11699800	-1.33581400	0.06792300
H	7.18939400	-1.13548200	0.11138700
H	5.82198500	-1.92183900	0.94946400
H	5.89322300	-1.91542500	-0.83845200
C	-3.76795400	-1.61797800	1.24498500
C	-3.09639200	-0.40683900	1.08072400
C	-3.12430200	0.27252900	-0.14728000
C	-3.83962100	-0.30001600	-1.20522200
C	-4.51513700	-1.51275800	-1.04629100
C	-4.48253500	-2.17559600	0.18098000
H	-3.73292100	-2.12811700	2.20427700
H	-2.53161600	0.01210800	1.90912000
H	-3.86867400	0.21005400	-2.16565900
H	-5.06563600	-1.93737500	-1.88189200
H	-5.00711600	-3.11878800	0.30870500
C	-2.40640000	1.60109200	-0.32528000
H	-2.65336600	1.97333900	-1.33257200
C	-2.92710700	2.66358000	0.67098300
H	-2.70693900	2.37690400	1.70482300
H	-2.45217700	3.63420100	0.48451900
H	-4.01117200	2.78605500	0.57418800

6-Br

C	-0.36197700	-3.82183400	0.64792100
C	0.00000000	-2.47192800	-0.00001700
C	0.36197200	-3.82181600	-0.64799500
H	0.18960700	-4.06808200	1.54864200
H	-1.41774200	-4.06938300	0.65315900
H	-0.18961400	-4.06803400	-1.54872400
H	1.41773600	-4.06936800	-0.65324100
C	-1.10716600	-1.70895600	-0.61880500
C	-3.06015700	-1.54459400	-1.76366800
C	-2.53247600	-0.14513900	-1.32829900
H	-3.01912600	-1.73277600	-2.83888600
H	-2.37605000	0.55621300	-2.15238100
C	1.10716800	-1.70897900	0.61879400

C	3.06017000	-1.54465700	1.76364500
C	2.53248400	-0.14518800	1.32833100
H	3.01915100	-1.73287600	2.83885700
H	2.37606300	0.55613500	2.15244000
O	-2.10340100	-2.46199700	-1.14717300
O	2.10340700	-2.46203800	1.14712900
N	-1.22318600	-0.43422100	-0.70416200
N	1.22319200	-0.43424600	0.70418900
C	-4.46409300	-1.72539100	-1.15867200
C	4.46410100	-1.72543000	1.15862800
C	-3.54941100	0.33992800	-0.31837500
C	-3.48867700	1.49828700	0.45643100
C	-4.60775700	-0.56504900	-0.19674200
C	-4.51932700	1.73749600	1.36871600
H	-2.64978600	2.18386500	0.36262900
C	-5.63598400	-0.32103700	0.71362800
C	-5.58356300	0.83571300	1.49640300
H	-4.49123300	2.63128600	1.98564800
H	-6.46422000	-1.01821700	0.81551500
H	-6.37708300	1.03668000	2.21147900
H	-5.22433100	-1.68760200	-1.94973800
H	-4.55216900	-2.70631900	-0.67813200
C	3.54940800	0.33991900	0.31841600
C	3.48866000	1.49830400	-0.45635000
C	4.60775600	-0.56505100	0.19674100
C	4.51930200	1.73755000	-1.36863500
H	2.64976400	2.18387300	-0.36252200
C	5.63597300	-0.32100200	-0.71362900
C	5.58354100	0.83577600	-1.49636200
H	4.49119700	2.63136000	-1.98553800
H	6.46421000	-1.01817500	-0.81554900
H	6.37705400	1.03677100	-2.21143800
H	5.22434700	-1.68767000	1.94968900
H	4.55217300	-2.70633900	0.67805000
Ni	0.00001800	0.98322500	0.00002100
Br	-0.00000700	3.30195300	-0.00002900

9

C	0.20675000	-2.54672700	3.05661000
C	0.03498500	-1.58269300	1.87478600
C	1.19881500	-2.59681300	1.95455000
H	0.49681700	-2.08791500	3.99491300
H	-0.53276200	-3.33647300	3.12341000
H	1.15151600	-3.41383200	1.24289500
H	2.18238300	-2.16494000	2.10339200
C	-1.02381500	-1.88678400	0.88607300
C	-2.39906900	-3.30249000	-0.23413400
C	-2.52780200	-1.84666600	-0.77356500
H	-1.94855800	-4.00772800	-0.93569000
H	-2.27520000	-1.72498300	-1.82946800
C	0.39108200	-0.16529000	2.09780700
C	1.61222800	1.47096700	3.04715000
C	0.61588700	2.06680800	2.01173600
H	1.51882700	1.88684600	4.05026800
H	-0.20560100	2.65989000	2.42130500
O	-1.44839600	-3.16904600	0.87296800
O	1.22156200	0.06828800	3.14127300
N	-1.52886800	-1.09422700	0.01645900
N	0.02388000	0.84961900	1.41041100
C	-3.78662500	-3.74120000	0.27310900
C	3.03277600	1.62805700	2.44868000

C	-3.94785300	-1.44736400	-0.45393200
C	-4.54915500	-0.21142200	-0.68368000
C	-4.63897500	-2.49450200	0.16188100
C	-5.87288400	-0.03575500	-0.27509200
H	-3.98994500	0.59831800	-1.13912700
C	-5.96260800	-2.31747500	0.56360500
C	-6.57318500	-1.07942900	0.34257500
H	-6.35636400	0.92430500	-0.43015400
H	-6.51133900	-3.12545000	1.04167100
H	-7.60262700	-0.92561900	0.65570200
H	-4.17874300	-4.55679000	-0.34780800
H	-3.71251500	-4.12758500	1.29622500
C	1.48661700	2.83756100	1.05038700
C	1.07592300	3.67256600	0.01147900
C	2.83987900	2.57987500	1.28819700
C	2.05449200	4.23853100	-0.81063000
H	0.01718700	3.84637600	-0.15953700
C	3.81485300	3.15802500	0.47559400
C	3.41245700	3.98228900	-0.58069000
H	1.75755200	4.88249400	-1.63333300
H	4.87015500	2.96486800	0.65237600
H	4.16202600	4.43189300	-1.22636000
H	3.75170700	1.99769700	3.18823000
H	3.39722700	0.65175700	2.10364100
Ni	-0.92707600	0.83769700	-0.40361100
C	0.83294900	0.37386400	-1.23372600
C	1.36680000	-0.84896800	-1.26035100
H	1.36873700	1.30987700	-1.36783000
H	0.69918200	-1.70847900	-1.25223700
C	2.79813400	-1.17381100	-1.18318900
C	3.20396200	-2.51663100	-1.18074700
C	3.80213800	-0.19179000	-1.03335800
C	4.54430300	-2.88656200	-1.04412500
H	2.45386300	-3.29677800	-1.29386700
C	5.13421400	-0.54335000	-0.89371900
H	3.53290300	0.85912500	-1.01500600
C	5.52095200	-1.89516200	-0.89761100
H	4.80906100	-3.93766700	-1.05440700
H	5.90493400	0.21273800	-0.77831600
O	6.85661100	-2.12784700	-0.74797000
C	7.30923700	-3.47240100	-0.76347400
H	8.39296400	-3.42633900	-0.64070000
H	6.87577200	-4.05496200	0.06142100
H	7.07274400	-3.96747300	-1.71530300
Br	-1.32480800	0.84804800	-2.78048400
Br	-2.49495900	2.56254800	0.39800900

7

C	-3.45641300	3.36847900	-0.98383400
C	-2.66854600	2.04467700	-1.00132200
C	-3.53683000	2.50615100	-2.18818400
H	-4.30326500	3.38899400	-0.30678800
H	-2.85835700	4.27160800	-1.02792200
H	-2.98697800	2.80395000	-3.07468200
H	-4.43920700	1.92925900	-2.35828100
C	-1.20617000	2.13914900	-1.15655300
C	0.68871900	3.11618700	-1.93015500
C	0.99951800	1.99017500	-0.91734500
H	0.90072700	2.87416100	-2.97495400
H	1.73024400	1.25570500	-1.25560600
C	-3.18988600	0.93986300	-0.17270400

C	-4.85197600	-0.19178200	0.86671700
C	-3.49898100	-0.94150700	0.97129300
H	-5.19273100	0.27843000	1.79271000
H	-3.19176300	-1.21613900	1.98086600
O	-0.76827000	3.23463800	-1.81353300
O	-4.53586500	0.89705500	-0.06265500
N	-0.31176000	1.32645900	-0.71209000
N	-2.51442500	0.02584900	0.42316000
C	1.36886600	4.39452300	-1.42567600
C	-5.87072400	-1.14659200	0.23234900
C	1.44608500	2.74305500	0.32116100
C	1.61854400	2.24676400	1.61277600
C	1.63440600	4.09959200	0.03517500
C	2.00526900	3.13511600	2.61959000
H	1.43627600	1.19938800	1.82532800
C	2.02233400	4.98330700	1.04101700
C	2.20996600	4.49088200	2.33578700
H	2.14197000	2.76883500	3.63322700
H	2.16900100	6.03897300	0.82581300
H	2.50870600	5.16900700	3.13084200
H	2.30132200	4.55945700	-1.98193400
H	0.73428300	5.27176300	-1.59281600
C	-3.68524300	-2.13743600	0.05516500
C	-2.72381500	-3.05549000	-0.36784300
C	-5.00971700	-2.22524900	-0.38778500
C	-3.11084300	-4.06542300	-1.25254200
H	-1.70382800	-2.97994500	-0.00614400
C	-5.39374800	-3.23510900	-1.26914000
C	-4.43411200	-4.15446000	-1.70168700
H	-2.37536800	-4.78897300	-1.59350200
H	-6.42176300	-3.30627100	-1.61647300
H	-4.71837500	-4.94528700	-2.39103200
H	-6.52877500	-1.55687200	1.00996600
H	-6.51006600	-0.61959700	-0.48457700
Ni	-0.50635000	-0.30348800	0.37724300
C	1.21759400	-0.85976400	-0.14389600
C	2.36331000	-1.05674100	0.52538300
H	1.21232300	-0.98116700	-1.23799800
H	2.34159100	-0.97215500	1.61168800
C	3.68756900	-1.38278500	-0.03341400
C	4.77353900	-1.57263100	0.83417500
C	3.94746000	-1.50339200	-1.41538900
C	6.05765200	-1.87079800	0.37084200
H	4.61030500	-1.48841300	1.90633700
C	5.21449300	-1.79923300	-1.89371100
H	3.13807800	-1.36609600	-2.12728500
C	6.28459400	-1.98595000	-1.00402000
H	6.86073800	-2.00966600	1.08598200
H	5.40797700	-1.89336400	-2.95824300
O	7.49343300	-2.27094700	-1.58046000
C	8.60330300	-2.48026700	-0.72594200
H	9.45168400	-2.69609900	-1.37910300
H	8.82825100	-1.58771100	-0.12474400
H	8.44144700	-3.33210200	-0.05028200
Br	-0.50214400	-1.74640000	2.22145000

11

C	4.00627100	3.10774600	-0.42997400
C	3.06837500	1.96969500	0.01765400
C	4.15037600	2.55854000	0.94114300
H	4.74434800	2.84057200	-1.17742200

H	3.53272900	4.07521300	-0.55267000
H	3.77753800	3.14072800	1.77610400
H	4.99176600	1.90680100	1.15087100
C	1.66789000	2.35000700	0.30527900
C	0.10029800	3.75994300	1.13717900
C	-0.53839300	2.50240200	0.49004700
H	0.05338300	3.77848000	2.22825100
H	-1.20425400	1.96133200	1.16359700
C	3.33469200	0.63008000	-0.55254300
C	4.72640800	-0.97971200	-1.29225000
C	3.24752800	-1.40728500	-1.46991500
H	5.31432300	-1.00672400	-2.20991200
H	2.87750000	-1.42542000	-2.49794200
O	1.51331000	3.60688800	0.78885500
O	4.63421900	0.41637200	-0.89497300
N	0.61316200	1.64368100	0.15391800
N	2.51035100	-0.32556400	-0.76796600
C	-0.50604300	4.99286200	0.44903800
C	5.31534500	-1.87242800	-0.16936300
C	-1.22369300	3.03146500	-0.75070500
C	-1.78126300	2.29376400	-1.79426800
C	-1.18063900	4.42894800	-0.78480000
C	-2.32288700	2.98935000	-2.87799700
H	-1.76256400	1.20869900	-1.77613300
C	-1.72540500	5.11922800	-1.86701000
C	-2.29991400	4.38927500	-2.91190900
H	-2.75450300	2.43487100	-3.70645000
H	-1.69716800	6.20573100	-1.90399500
H	-2.72329400	4.91445400	-3.76427200
H	-1.22890000	5.47536900	1.12047600
H	0.26761800	5.73588800	0.22520000
C	3.15611500	-2.74374200	-0.78491700
C	2.09825500	-3.64928000	-0.82643600
C	4.31062500	-2.99953800	-0.03756800
C	2.20649000	-4.82900000	-0.08472000
H	1.21462600	-3.41922800	-1.41603700
C	4.41609000	-4.17745100	0.70042900
C	3.35371800	-5.08767400	0.67502200
H	1.39390400	-5.54997600	-0.09754900
H	5.30844600	-4.39086500	1.28380800
H	3.42405000	-6.00974800	1.24592900
H	6.32338100	-2.22755600	-0.41050500
H	5.39592200	-1.28739000	0.75622300
Ni	0.37394900	-0.51206900	-0.36861400
C	-1.47083400	-0.64491500	0.17951300
C	-2.44002300	-1.34902500	-0.41843200
H	-1.68423300	-0.04707100	1.06676400
H	-2.19063900	-1.92639600	-1.30655100
C	-3.85739000	-1.39096200	-0.01669500
C	-4.80051500	-1.96938400	-0.87891300
C	-4.33540300	-0.87892800	1.20821900
C	-6.15917200	-2.03229000	-0.56069100
H	-4.46386500	-2.37634900	-1.82963300
C	-5.67996000	-0.93147300	1.53997100
H	-3.63715900	-0.43844200	1.91477400
C	-6.60708800	-1.50776900	0.65633300
H	-6.84821200	-2.48742600	-1.26313600
H	-6.04455100	-0.53972200	2.48483200
O	-7.90679000	-1.51079500	1.08103200
C	-8.88488900	-2.08167900	0.22905600
H	-9.83800100	-1.97836200	0.75201000

H	-8.94165000	-1.55506900	-0.73395600
H	-8.68864400	-3.14681900	0.04191400
C	0.71852900	-1.43197500	1.56529900
H	1.73763300	-1.70412200	1.28227200
C	0.71604800	-0.35795100	2.57388800
C	1.88987900	0.39168000	2.80284500
C	-0.42907600	-0.00759300	3.32005700
C	1.91152400	1.46178300	3.69077400
H	2.78975400	0.12634600	2.25428000
C	-0.41228200	1.07143700	4.20518000
H	-1.33795300	-0.58990900	3.21209300
C	0.75268100	1.82009000	4.39028400
H	2.83368000	2.01632500	3.84613200
H	-1.31249000	1.32151200	4.76119600
H	0.76646000	2.65370900	5.08729400
C	-0.11050500	-2.67720100	1.82296800
H	-0.11115300	-3.32244100	0.94142400
H	0.32641700	-3.24542000	2.65885200
H	-1.15237700	-2.45879000	2.06445000
Br	0.05704200	-1.05193200	-2.80568000

L1·NiBr$_2$

C	0.35281100	-4.05239100	-0.65177700
C	-0.00013400	-2.70088800	0.00001300
C	-0.35326200	-4.05231400	0.65186400
H	-0.21169000	-4.29370300	-1.54590800
H	1.40866000	-4.29806600	-0.67366300
H	0.21120400	-4.29366300	1.54600700
H	-1.40914600	-4.29784200	0.67376300
C	1.10618800	-1.93484000	0.60579700
C	3.08896300	-1.75477500	1.69404700
C	2.54537800	-0.35989500	1.28288300
H	3.06155700	-1.95980700	2.76632300
H	2.42435000	0.35224900	2.09963700
C	-1.10635800	-1.93472200	-0.60579600
C	-3.08902800	-1.75444900	-1.69419300
C	-2.54538600	-0.35964000	-1.28287600
H	-3.06148000	-1.95943200	-2.76647500
H	-2.42432000	0.35258100	-2.09955700
O	2.12640500	-2.67546300	1.07689300
O	-2.12663700	-2.67523700	-1.07693100
N	1.20169600	-0.65967400	0.72599800
N	-1.20172900	-0.65954600	-0.72599400
C	4.47793200	-1.92368200	1.05843500
C	-4.47809300	-1.92327800	-1.05878300
C	3.50385000	0.10838900	0.20798400
C	3.40038500	1.24656700	-0.59231000
C	4.56102600	-0.79421500	0.05383800
C	4.37780900	1.46286200	-1.56675500
H	2.57687600	1.94162100	-0.46077800
C	5.53802000	-0.57207400	-0.91589400
C	5.43696900	0.56115600	-1.72801400
H	4.31078200	2.33923800	-2.20491400
H	6.36378200	-1.26826300	-1.04134900
H	6.18861900	0.74382000	-2.49142300
H	5.25424300	-1.83256200	1.82931600
H	4.58347600	-2.92005600	0.61476900
C	-3.50388000	0.10856900	-0.20795400
C	-3.40036300	1.24660500	0.59254000
C	-4.56115600	-0.79395600	-0.05402200
C	-4.37783100	1.46283000	1.56695600

H	-2.57678300	1.94160900	0.46118700
C	-5.53819400	-0.57188300	0.91568200
C	-5.43709000	0.56120200	1.72799600
H	-4.31076100	2.33909600	2.20526300
H	-6.36402900	-1.26801600	1.04096500
H	-6.18877700	0.74381800	2.49138000
H	-5.25429200	-1.83194800	-1.82975200
H	-4.58381600	-2.91970400	-0.61527500
Ni	0.00005300	0.77347900	0.00001300
Br	-0.60168700	2.35892200	-1.66454900
Br	0.60199600	2.35881200	1.66461900

L1·NiCl$_2$

C	0.36934700	3.67764800	0.64258800
C	0.00000100	2.32595500	0.00000000
C	-0.36934600	3.67764100	-0.64260100
H	-0.17237100	3.91868200	1.55077700
H	1.42551800	3.92296700	0.63776900
H	0.17237100	3.91866800	-1.55079200
H	-1.42551800	3.92295900	-0.63778400
C	1.09063800	1.55889300	-0.63047100
C	3.04198500	1.37425300	-1.77216800
C	2.52420300	-0.01632400	-1.31750100
H	2.97370800	1.56203300	-2.84586200
H	2.39777600	-0.75012700	-2.11317000
C	-1.09063600	1.55889700	0.63047700
C	-3.04198700	1.37426400	1.77216700
C	-2.52420400	-0.01631400	1.31750900
H	-2.97371400	1.56205000	2.84586000
H	-2.39778000	-0.75011300	2.11318300
O	2.09531600	2.29789400	-1.13382400
O	-2.09531400	2.29790200	1.13382200
N	1.18788800	0.28228600	-0.74021100
N	-1.18788900	0.28229100	0.74022000
C	4.44989400	1.56501900	-1.18871300
C	-4.44989300	1.56502700	1.18870500
C	3.51063200	-0.44384900	-0.24985100
C	3.43503000	-1.55628900	0.58899900
C	4.56515000	0.46973700	-0.15032000
C	4.43788000	-1.73471100	1.54536000
H	2.61538800	-2.26205000	0.49551000
C	5.56698100	0.28560300	0.80180900
C	5.49408000	-0.82146100	1.65198300
H	4.39445600	-2.59166900	2.21140600
H	6.39045800	0.99085500	0.88432000
H	6.26565400	-0.97493500	2.40182100
H	5.20011200	1.44661000	-1.98147600
H	4.56967100	2.57594200	-0.78328300
C	-3.51062700	-0.44384400	0.24985700
C	-3.43501700	-1.55628800	-0.58898700
C	-4.56514300	0.46974100	0.15031400
C	-4.43786000	-1.73471500	-1.54535500
H	-2.61537500	-2.26204700	-0.49549000
C	-5.56696800	0.28560300	-0.80182100
C	-5.49406000	-0.82146500	-1.65198900
H	-4.39443100	-2.59167400	-2.21139800
H	-6.39044400	0.99085500	-0.88434100
H	-6.26562700	-0.97494200	-2.40183300
H	-5.20011400	1.44661500	1.98146500
H	-4.56967300	2.57595000	0.78327400
Ni	-0.00000200	-1.14356200	0.00000300

Cl	-0.71978600	-2.66568700	1.48657600
Cl	0.71975700	-2.66569100	-1.48657800

TS1

C	-1.05584700	0.89662900	-3.79898300
C	-0.92083500	1.17557100	-2.29343800
C	-1.06769600	2.30559400	-3.32646300
H	-0.16620700	0.51693600	-4.28908200
H	-1.99528800	0.44884900	-4.10598300
H	-2.00856000	2.84351200	-3.30064600
H	-0.17975200	2.90975700	-3.48015400
C	-2.09530300	0.83643400	-1.46137000
C	-4.29566400	0.90548800	-0.94148600
C	-3.51430500	-0.02258700	0.02688400
H	-4.59199000	1.86533200	-0.51392300
H	-3.63456200	0.22184900	1.08420100
C	0.41473600	1.05373700	-1.67121800
C	2.66920900	0.85510800	-1.71649500
C	2.14510400	1.10600700	-0.28042400
H	2.98683100	-0.17258200	-1.91186600
H	2.49463200	0.37043000	0.44594000
O	-3.28871500	1.20140000	-1.96844900
O	1.46357000	1.08268800	-2.52378900
N	-2.09915500	0.19721600	-0.34813100
N	0.67950300	0.99420600	-0.41813200
C	-5.44190700	0.09126400	-1.55083300
C	3.72885000	1.92119100	-2.01636800
C	-3.99803800	-1.41188500	-0.34834300
C	-3.50273900	-2.64360700	0.07883900
C	-5.05237000	-1.34284600	-1.26646300
C	-4.07447500	-3.81104700	-0.43326900
H	-2.69797700	-2.68765900	0.80437500
C	-5.62274600	-2.50862700	-1.77612800
C	-5.12515800	-3.74506400	-1.35621100
H	-3.70012100	-4.77862100	-0.11001500
H	-6.44092900	-2.45778200	-2.49048300
H	-5.55868000	-4.66163200	-1.74753900
H	-6.38671300	0.35341000	-1.05667800
H	-5.56293400	0.31617000	-2.61610300
C	2.60104300	2.51909500	0.02464300
C	2.22385600	3.33003400	1.09353800
C	3.48199100	2.97826000	-0.96083800
C	2.76054300	4.61793600	1.16938500
H	1.48841300	2.98993300	1.81581200
C	4.01768500	4.26278800	-0.88054000
C	3.65324700	5.07902500	0.19418300
H	2.46940700	5.27051000	1.98761100
H	4.70094500	4.62772300	-1.64372300
H	4.05865700	6.08488700	0.26687600
H	4.73044300	1.48068300	-1.92230800
H	3.63604500	2.29146900	-3.04328800
Ni	-0.62992500	0.39158400	1.09701700
C	0.14520600	-1.46090200	1.05306000
C	1.40732100	-1.61682700	1.50717100
H	-0.31299900	-2.04962100	0.26152600
H	1.68174300	-1.10086300	2.42747200
C	2.49157700	-2.32559200	0.82522800
C	3.74667800	-2.44072900	1.44634600
C	2.36658500	-2.85002800	-0.48193600
C	4.82896100	-3.06044800	0.82009200
H	3.87940700	-2.03847100	2.44795200

C	3.43208400	-3.46857700	-1.11546300
H	1.42078100	-2.76087600	-1.00897100
C	4.67635700	-3.58135200	-0.47045400
H	5.77552200	-3.12906600	1.34371200
H	3.33380500	-3.87313600	-2.11815400
O	5.65806800	-4.20007800	-1.18629800
C	6.93347900	-4.35406700	-0.58259200
H	7.55201700	-4.87442800	-1.31620500
H	7.38965200	-3.38248300	-0.34759100
H	6.87577700	-4.95503500	0.33512900
Br	-1.44118600	-1.23197500	2.80236100
Br	-1.30366700	2.56382000	1.96385100

TS2-*S*

C	3.06533600	3.75139600	-0.25459700
C	2.43773300	2.38254800	0.08018700
C	3.34577300	3.13862400	1.06689800
H	3.84222100	3.73508700	-1.00992800
H	2.36947800	4.58111900	-0.30266600
H	2.84254900	3.53642500	1.94074400
H	4.32116600	2.69577400	1.23789300
C	0.98949600	2.38723100	0.34882200
C	-0.90790000	3.32868100	1.14922800
C	-1.20355200	2.05954800	0.31764600
H	-1.01878000	3.20317500	2.22812000
H	-1.83818800	1.33816600	0.82819300
C	3.01699600	1.19812400	-0.58757000
C	4.71129500	0.16140800	-1.67453800
C	3.49998200	-0.79599300	-1.47785700
H	4.88489600	0.46227000	-2.71024600
H	3.07288700	-1.19479400	-2.39845100
O	0.52167700	3.52713300	0.89863900
O	4.30538100	1.36373500	-0.95864400
N	0.13497500	1.46911400	0.07213000
N	2.46071500	0.06827000	-0.83920100
C	-1.71954400	4.47901300	0.54038100
C	5.94182600	-0.46804400	-1.00032700
C	-1.81501400	2.60062400	-0.95816300
C	-2.06009600	1.91699000	-2.14808600
C	-2.08142200	3.96888000	-0.83883600
C	-2.59813500	2.62800800	-3.22388600
H	-1.82825000	0.86058600	-2.22544100
C	-2.61980300	4.67550500	-1.91329000
C	-2.87849700	3.99502200	-3.10681700
H	-2.79664300	2.11477300	-4.16077900
H	-2.82906800	5.73923700	-1.82895300
H	-3.29487300	4.53427700	-3.95368700
H	-2.61429200	4.66205300	1.15040800
H	-1.14110900	5.40943500	0.53072400
C	4.04047400	-1.88375500	-0.57030500
C	3.37611200	-3.01122400	-0.08701500
C	5.38561300	-1.66010800	-0.25579000
C	4.05976700	-3.88257900	0.76377800
H	2.35079300	-3.19728000	-0.38330500
C	6.06861400	-2.53117300	0.59287900
C	5.39348000	-3.63875900	1.11226200
H	3.54949600	-4.75863800	1.15459900
H	7.11341300	-2.35751100	0.83923700
H	5.91280700	-4.32347200	1.77762700
H	6.67311400	-0.78228400	-1.75590000
H	6.44273400	0.26409600	-0.35662100

Ni	0.44699700	-0.43770500	-0.57422900
C	-1.34020500	-0.88513600	-0.08926900
C	-2.37633600	-1.35969400	-0.79547900
H	-1.50812900	-0.62470400	0.95860100
H	-2.21484800	-1.65504500	-1.83081300
C	-3.75781400	-1.53355200	-0.30880400
C	-4.72552000	-2.08282400	-1.16277400
C	-4.18447600	-1.16739000	0.98628500
C	-6.05207300	-2.27175300	-0.76606700
H	-4.43240400	-2.37656000	-2.16825500
C	-5.49578300	-1.34795200	1.39760200
H	-3.47324100	-0.73300000	1.68389900
C	-6.44479700	-1.90323100	0.52425000
H	-6.75881300	-2.70301700	-1.46618000
H	-5.81838800	-1.06543100	2.39544400
O	-7.71013800	-2.03640100	1.02945800
C	-8.70447200	-2.59637600	0.19038300
H	-9.62325400	-2.61462100	0.78063000
H	-8.86601900	-1.98803700	-0.71091500
H	-8.44975300	-3.62154800	-0.11369100
C	0.96679000	-1.54179300	1.75743500
H	1.98701400	-1.52659300	1.38724500
C	0.62295100	-0.48967300	2.66278100
C	1.54986900	0.56303300	2.90315300
C	-0.62920900	-0.40880200	3.33182800
C	1.24074800	1.62863500	3.73492800
H	2.51855300	0.51957200	2.41134200
C	-0.93846000	0.67044400	4.15425700
H	-1.35556900	-1.20472400	3.20600700
C	-0.01266400	1.70002100	4.36291900
H	1.97704600	2.40938300	3.90913800
H	-1.90633100	0.70466400	4.64852400
H	-0.25278200	2.53250400	5.01856300
C	0.26399300	-2.86369700	1.67067800
H	0.35700400	-3.28125500	0.66461400
H	0.70717600	-3.57850200	2.38380900
H	-0.80484800	-2.79535300	1.88649100
Br	0.56273400	-2.22066500	-2.18652600

TS2-*S*-cf2

C	-5.24604100	0.93263800	-1.09771400
C	-3.78020100	0.46035300	-1.07308800
C	-4.72742500	0.15407600	-2.24963500
H	-5.90476600	0.43784500	-0.39323900
H	-5.38939800	2.00062000	-1.21509500
H	-4.50794100	0.67868100	-3.17351100
H	-5.02398900	-0.88431700	-2.35061300
C	-2.74655500	1.49274900	-1.29434100
C	-1.98700200	3.42406000	-2.20753000
C	-0.96486100	2.81826100	-1.20851700
H	-1.70266200	3.34862100	-3.26044300
H	0.03323200	2.68154400	-1.62838100
C	-3.45812900	-0.65564400	-0.15661300
C	-3.95584800	-2.58543100	0.89349500
C	-2.56383100	-2.06153000	1.33506700
H	-4.65722600	-2.72712100	1.71631600
H	-2.51102600	-1.67969700	2.35673600
O	-3.15146000	2.55628200	-2.02782300
O	-4.48810900	-1.50756000	0.07301600
N	-1.53755500	1.49752100	-0.86628200
N	-2.34852700	-0.89515100	0.43738200

C	-2.29907900	4.85454200	-1.74476600
C	-3.71530200	-3.88040700	0.08024000
C	-0.99473400	3.77315600	-0.03206800
C	-0.40792700	3.60279400	1.22151300
C	-1.76991900	4.89979400	-0.32721500
C	-0.60415400	4.59756500	2.18260700
H	0.15652200	2.70512100	1.45229500
C	-1.95947700	5.89195900	0.63363600
C	-1.36958100	5.73318900	1.89111600
H	-0.16606000	4.47731500	3.16917200
H	-2.56087000	6.77063600	0.41295500
H	-1.51413000	6.49578700	2.65203700
H	-1.78308600	5.57546900	-2.39295200
H	-3.37165200	5.06429800	-1.82287900
C	-1.63701400	-3.22968500	1.11404200
C	-0.32240600	-3.37122400	1.55443700
C	-2.29174100	-4.26218300	0.43149700
C	0.33339800	-4.58010100	1.30880200
H	0.16271500	-2.55238700	2.07634500
C	-1.63083300	-5.46401400	0.17928500
C	-0.31473600	-5.61749800	0.62868500
H	1.35947900	-4.70970400	1.63921900
H	-2.13241000	-6.27485600	-0.34340400
H	0.20868400	-6.55261500	0.44675600
H	-4.44056000	-4.66386700	0.32549000
H	-3.82651700	-3.66274500	-0.99024600
Ni	-0.57037800	0.09954800	0.34237500
C	1.19758600	0.77276100	0.08725800
C	2.29005800	0.49311700	0.81753900
H	1.34097700	1.32648500	-0.84872600
H	2.16264300	-0.06577100	1.74245300
C	3.68876100	0.77871900	0.45664600
C	4.72233100	0.11693400	1.13625400
C	4.06738100	1.64744600	-0.58872200
C	6.06653300	0.27757500	0.79072700
H	4.46646400	-0.55885700	1.94895800
C	5.39590500	1.81982100	-0.94714600
H	3.30220700	2.20003800	-1.12725200
C	6.41049900	1.13107300	-0.26287700
H	6.82529300	-0.26400500	1.34449200
H	5.68159500	2.49075000	-1.75189700
O	7.68691800	1.36894400	-0.69721200
C	8.74819700	0.70512800	-0.03467800
H	9.66530500	1.03134600	-0.53002100
H	8.79619600	0.97591900	1.02958500
H	8.66069100	-0.38751700	-0.11963100
C	0.02816900	-1.10197600	-1.99384200
H	0.16064700	-0.07561300	-2.32286600
C	1.23175600	-1.87556800	-1.83920300
C	2.50292300	-1.25761600	-1.95384800
C	1.20378100	-3.26829100	-1.58674900
C	3.67706700	-1.97925900	-1.79379100
H	2.55788800	-0.19373500	-2.15525100
C	2.38227300	-3.99083100	-1.43611600
H	0.25316500	-3.77895600	-1.48118200
C	3.62487900	-3.35413400	-1.53162100
H	4.63222900	-1.46575800	-1.85857900
H	2.33047900	-5.05709900	-1.23090100
H	4.54247300	-3.92228100	-1.40350700
C	-1.26706300	-1.75529500	-2.38832200
H	-2.06450400	-1.01734700	-2.49361900

H	-1.15810800	-2.26936900	-3.35697800
H	-1.59331200	-2.50707600	-1.66326800
Br	-0.44406200	0.11836600	2.80762700

TS2-*S*-cf3

C	-0.47501300	2.80575700	3.30454100
C	-0.91695400	2.30951000	1.91624500
C	-1.67712900	3.41112500	2.67567000
H	-0.61024400	2.10347700	4.11993700
H	0.44560300	3.37997800	3.32015500
H	-1.59936900	4.40606100	2.25314900
H	-2.65391400	3.12822000	3.05175500
C	-0.06998900	2.72342100	0.77504700
C	0.96474700	4.28006000	-0.49971100
C	1.27756800	2.84038900	-0.99239300
H	0.27193000	4.83662200	-1.13485400
H	1.02455900	2.66503100	-2.04073700
C	-1.60267300	1.00403500	1.83219500
C	-2.85384100	-0.67757300	2.69557100
C	-2.66246100	-0.82548600	1.16991900
H	-2.26255700	-1.36870200	3.30234700
H	-2.30112200	-1.80473600	0.86803200
O	0.26562000	4.03419900	0.76312200
O	-2.31380200	0.66269700	2.93519900
N	0.39620500	1.98574700	-0.16097800
N	-1.65885000	0.20273300	0.83144800
C	2.29793000	4.99243100	-0.23850800
C	-4.36105700	-0.72438000	2.97740900
C	2.74952200	2.66870500	-0.66665800
C	3.51999100	1.51158000	-0.75572400
C	3.30765900	3.86638300	-0.20588000
C	4.85776700	1.55627900	-0.35721700
H	3.07449600	0.58488600	-1.09092100
C	4.64576200	3.91554300	0.18410600
C	5.41685900	2.75155600	0.11079700
H	5.45586500	0.65005600	-0.40177800
H	5.08274300	4.84365900	0.54515300
H	6.45803600	2.77564800	0.42216100
H	2.51020000	5.69618700	-1.05429700
H	2.25490200	5.57750700	0.68685900
C	-4.02804700	-0.48532300	0.60918600
C	-4.36323900	-0.19793700	-0.71238300
C	-4.98323300	-0.43032100	1.62852400
C	-5.69301400	0.11018000	-1.00868400
H	-3.59734900	-0.16207700	-1.48013800
C	-6.31143000	-0.12717600	1.33040600
C	-6.66179100	0.13471300	0.00272400
H	-5.97284900	0.34671600	-2.03153200
H	-7.06062600	-0.08201100	2.11725600
H	-7.69214900	0.37777400	-0.24355300
H	-4.63263300	-1.72489200	3.34003100
H	-4.64241300	-0.00964800	3.75859600
Ni	-0.43574700	0.13162200	-0.87840800
C	1.01519800	-0.96171900	-0.10574800
C	2.04267300	-1.55781600	-0.73849400
H	1.08599800	-0.87082900	0.98616300
H	2.02095000	-1.62319100	-1.83192500
C	3.30171100	-2.06014300	-0.15285600
C	4.40947000	-2.29128700	-0.98127800
C	3.47755000	-2.28007300	1.22896200
C	5.64727600	-2.70041800	-0.47553000

H	4.30862500	-2.13483400	-2.05347700
C	4.69504400	-2.69143000	1.74810300
H	2.63940900	-2.12598700	1.90267900
C	5.79557200	-2.90077600	0.90085500
H	6.47385700	-2.86011500	-1.15876900
H	4.82606800	-2.86218700	2.81248500
O	6.95042700	-3.29973900	1.51595100
C	8.09042500	-3.52921400	0.70660600
H	8.88821400	-3.83347200	1.38742200
H	7.91470300	-4.33011000	-0.02542300
H	8.39880500	-2.61958000	0.17185900
C	-1.03792700	-2.22098700	-2.20683500
H	0.04302000	-2.27980200	-2.19687400
C	-1.71452400	-3.03305200	-1.22790200
C	-3.11634700	-3.24115700	-1.25635500
C	-0.97686400	-3.66936900	-0.19176800
C	-3.74802900	-4.01123400	-0.28423800
H	-3.71289300	-2.77234000	-2.03052200
C	-1.61287700	-4.44456000	0.76839600
H	0.09515400	-3.51032100	-0.14714500
C	-3.00571700	-4.61388200	0.73658000
H	-4.82702800	-4.13561600	-0.31873700
H	-1.02567800	-4.91938500	1.55049200
H	-3.50239100	-5.21473700	1.49369900
C	-1.67313000	-1.85503000	-3.51482600
H	-1.82654100	-2.76770300	-4.11535800
H	-2.64517800	-1.36835500	-3.39782000
H	-1.04290800	-1.16617700	-4.07804600
Br	-1.53281300	1.39671500	-2.66981400

TS2-*R*

C	4.22818600	2.95283600	1.33414200
C	3.18356600	1.83386900	1.16632000
C	4.10022600	1.93023200	2.40160700
H	5.07893700	2.89766400	0.66480300
H	3.82535500	3.94320900	1.51214100
H	3.60416200	2.20321200	3.32702500
H	4.86238600	1.16238400	2.47959900
C	1.77025100	2.21715000	1.34853600
C	0.11011700	3.47711000	2.24200700
C	-0.41614800	2.54064400	1.12569800
H	-0.16215500	3.18544500	3.25996600
H	-1.27687800	1.93439100	1.40867100
C	3.49150800	0.77343100	0.17975100
C	4.93449400	-0.58545600	-0.89311200
C	3.47790700	-0.84540200	-1.37080100
H	5.62177100	-0.30810000	-1.69291400
H	3.25789500	-0.54356700	-2.39652100
O	1.56199400	3.30125300	2.13029500
O	4.81611300	0.57575500	-0.02482100
N	0.73657800	1.65885700	0.83299700
N	2.67228000	0.03861900	-0.47876200
C	-0.28571700	4.91117200	1.86837500
C	5.39312200	-1.83634700	-0.10543400
C	-0.70272700	3.48572200	-0.02532200
C	-0.97249000	3.15932200	-1.35400500
C	-0.60495100	4.81774300	0.39145000
C	-1.16281400	4.20186000	-2.26460100
H	-1.00363000	2.12333900	-1.67264500
C	-0.79959500	5.85488700	-0.51944800
C	-1.08251100	5.53726400	-1.85116500

H	-1.36596300	3.96896300	-3.30606200
H	-0.72454500	6.89263900	-0.20340200
H	-1.23144800	6.33543600	-2.57379900
H	-1.16319500	5.21540600	2.45476500
H	0.51901200	5.61618500	2.10438400
C	3.26929600	-2.32335300	-1.13831600
C	2.20105500	-3.11743300	-1.55822600
C	4.32682600	-2.86790800	-0.40232700
C	2.19214700	-4.46753900	-1.19878300
H	1.39258300	-2.67481000	-2.13125200
C	4.31576900	-4.21613100	-0.04584700
C	3.23669500	-5.01137600	-0.44166400
H	1.36478300	-5.09966900	-1.50898300
H	5.13444400	-4.64494800	0.52696700
H	3.21393500	-6.06268300	-0.16740200
H	6.39207300	-2.16756300	-0.41130400
H	5.45139800	-1.59514100	0.96366900
Ni	0.60934800	0.05817200	-0.42880600
C	-1.26167500	-0.14690900	-0.10097900
C	-2.30371300	-0.26818400	-0.93807100
H	-1.45843900	-0.17467300	0.97684300
H	-2.11876000	-0.22913000	-2.00981100
C	-3.71622900	-0.45603400	-0.56020800
C	-4.71807700	-0.32025600	-1.53241900
C	-4.13104800	-0.79107200	0.74594100
C	-6.07359800	-0.48741600	-1.23479700
H	-4.43151900	-0.07317900	-2.55243700
C	-5.47048800	-0.96335500	1.05828600
H	-3.38703700	-0.94706500	1.51952100
C	-6.45672600	-0.80942500	0.07101600
H	-6.80902200	-0.36785300	-2.02264900
H	-5.78361800	-1.23278900	2.06286500
O	-7.74855400	-1.00341700	0.48551600
C	-8.77977700	-0.87281500	-0.47553600
H	-9.71489600	-1.06644500	0.05488000
H	-8.81114300	0.13889800	-0.90479500
H	-8.67034700	-1.60084200	-1.29210200
C	0.88829200	-1.80576800	1.50093000
C	-0.37012000	-2.44243300	1.75272300
C	-1.08484900	-2.26467100	2.96391900
C	-0.95457200	-3.27636200	0.76343700
C	-2.30006700	-2.90993500	3.18178100
H	-0.67330000	-1.62885600	3.74232200
C	-2.16997700	-3.90583200	0.98144600
H	-0.44032400	-3.39050200	-0.18582300
C	-2.85072300	-3.73342600	2.19559900
H	-2.82491300	-2.76483300	4.12287200
H	-2.60319900	-4.52581600	0.20140200
H	-3.80739800	-4.22071100	2.35989900
C	1.62165000	-1.05690700	2.57420500
H	1.05265700	-0.19133100	2.93714000
H	1.81814500	-1.69938800	3.44725100
H	2.58358700	-0.69295700	2.20896000
H	1.47579600	-2.20432600	0.68354400
Br	0.49764500	-0.21368200	-2.86300700

TS2-*R*-cf2

C	4.61806900	2.25812300	0.60197100
C	3.37474000	1.35362400	0.64547200
C	4.29028600	1.55676400	1.86886400
H	5.44315100	1.88693400	0.00490800

H	4.41891800	3.32128300	0.53226300
H	3.85892600	2.13038000	2.68246400
H	4.88486800	0.69611100	2.15601400
C	2.05899600	2.02838300	0.67508300
C	0.68984600	3.75653800	1.20908500
C	-0.05381500	2.66422000	0.39603400
H	0.38796300	3.81603300	2.25498200
H	-0.93675300	2.28253700	0.91035600
C	3.46816500	0.05391000	-0.05514600
C	4.62186600	-1.72761000	-0.84650200
C	3.10916500	-1.84143100	-1.19080400
H	5.27264500	-1.64899100	-1.71859400
H	2.87001600	-1.80945500	-2.25507300
O	2.07253900	3.27406100	1.20366000
O	4.72152900	-0.45018600	-0.14762200
N	0.93544500	1.57407900	0.26011200
N	2.51551400	-0.62810000	-0.57145400
C	0.56148300	5.08104800	0.43954500
C	4.98595400	-2.91163700	0.07805700
C	-0.34392100	3.32173500	-0.93700200
C	-0.84236800	2.72537500	-2.09574100
C	0.02679100	4.67051900	-0.91626000
C	-0.98611000	3.51596600	-3.23862000
H	-1.07740900	1.66569200	-2.11962800
C	-0.12241500	5.45711100	-2.05785600
C	-0.63468700	4.87114800	-3.21899200
H	-1.36378400	3.06709900	-4.15281600
H	0.16242500	6.50657500	-2.04935000
H	-0.75077500	5.47140600	-4.11769200
H	-0.13327900	5.75106400	0.96307000
H	1.52680100	5.59747700	0.39026200
C	2.65934500	-3.11805700	-0.52467600
C	1.38197600	-3.67852900	-0.54102500
C	3.69683300	-3.69415100	0.21430000
C	1.15048200	-4.82762800	0.21864400
H	0.59602800	-3.21165400	-1.12667500
C	3.46430500	-4.84563000	0.96673800
C	2.18288700	-5.40455900	0.96928600
H	0.16119600	-5.27683500	0.22658200
H	4.26320200	-5.29992700	1.54778900
H	1.98761800	-6.29760200	1.55704300
H	5.78994800	-3.51832300	-0.35582900
H	5.35451400	-2.53511000	1.03985500
Ni	0.50392500	-0.26841200	-0.58224000
C	-1.39415800	-0.17808200	-0.41733900
C	-2.29751800	-1.09707200	-0.79584600
H	-1.74718000	0.68600700	0.14846000
H	-1.95679300	-1.95601700	-1.37243900
C	-3.73975200	-1.09323200	-0.49147600
C	-4.51997900	-2.21156300	-0.82307800
C	-4.40250700	-0.01969300	0.14397200
C	-5.88545300	-2.28244900	-0.53578300
H	-4.04510100	-3.05418800	-1.32098500
C	-5.75652700	-0.07285300	0.43721800
H	-3.84391600	0.87236200	0.40975000
C	-6.51276200	-1.20754500	0.10240300
H	-6.44066600	-3.17132700	-0.81374000
H	-6.26135800	0.75804600	0.92132400
O	-7.83865800	-1.15769700	0.43765300
C	-8.65030600	-2.27053500	0.10684700
H	-9.65660000	-2.02426100	0.45256300

H	-8.67392100	-2.44829600	-0.97761300
H	-8.30953500	-3.18655200	0.61015300
C	0.25062300	-1.10137500	1.94414400
C	-0.48815300	-0.07298100	2.63343100
C	0.15497900	0.94275700	3.38612900
C	-1.90626200	-0.04586000	2.59081300
C	-0.57573500	1.93430300	4.03365400
H	1.23818400	0.95268800	3.45754000
C	-2.63176500	0.95239800	3.23201700
H	-2.43019400	-0.81768600	2.03871900
C	-1.97378100	1.95617300	3.95281200
H	-0.05393100	2.69536700	4.60985100
H	-3.71666000	0.94223600	3.16853300
H	-2.54078600	2.73429100	4.45635000
C	1.64870400	-1.44437500	2.37497900
H	2.33135000	-0.58990400	2.31643900
H	1.65008100	-1.77365600	3.42690400
H	2.06438800	-2.24862800	1.77109900
H	-0.33284800	-1.90584700	1.50794100
Br	0.34383600	-1.00613000	-2.91029500

TS2-*R*-cf3

C	-0.39683000	4.14318800	2.26550300
C	-0.78091500	2.98313200	1.33267000
C	-1.71213200	4.18486200	1.57599000
H	-0.37001900	3.90835100	3.32371900
H	0.39652400	4.78695900	1.90093500
H	-1.84023600	4.85211400	0.73126000
H	-2.60572100	3.97051800	2.15238200
C	-0.06979300	2.91798100	0.03741100
C	0.72092400	3.83054300	-1.87658400
C	1.08255800	2.32239800	-1.77055100
H	-0.05688700	4.05837400	-2.60804300
H	0.72242700	1.72320100	-2.61079100
C	-1.20771800	1.70897200	1.94754900
C	-1.94680000	0.44661200	3.68114700
C	-1.92642300	-0.35010400	2.35224100
H	-1.15470600	0.18505300	4.38782500
H	-1.37581400	-1.29114200	2.40546000
O	0.14560200	4.11264600	-0.55909400
O	-1.66286300	1.80683200	3.22125300
N	0.37392000	1.87387700	-0.55354200
N	-1.25088200	0.54944100	1.40261200
C	2.02633400	4.61495000	-2.07226800
C	-3.36398400	0.34121900	4.26341600
C	2.58838100	2.32243000	-1.59858000
C	3.41813800	1.24057300	-1.30754100
C	3.10887900	3.61463600	-1.72804000
C	4.78584600	1.46994300	-1.13632900
H	3.00192600	0.24690900	-1.18621500
C	4.47508500	3.84216000	-1.56360300
C	5.31033300	2.76208200	-1.26363600
H	5.43779400	0.63758500	-0.88729400
H	4.88494200	4.84470800	-1.66146700
H	6.37564500	2.92789200	-1.12534000
H	2.10817300	4.94971300	-3.11479700
H	2.04003200	5.51354100	-1.44548200
C	-3.39414100	-0.51543300	2.01621600
C	-3.94736900	-0.91427200	0.80213700
C	-4.20042800	-0.12575000	3.09053600
C	-5.33854000	-0.97345200	0.69201500

H	-3.31722900	-1.12128700	-0.05427100
C	-5.58899100	-0.18211800	2.97824000
C	-6.15236600	-0.61952800	1.77481200
H	-5.78214200	-1.28426800	-0.24948500
H	-6.22550800	0.12017500	3.80637800
H	-7.23365100	-0.66534100	1.67431600
H	-3.37125800	-0.38521800	5.08709800
H	-3.69007000	1.30093500	4.67919700
Ni	-0.44571300	-0.10150200	-0.41657600
C	1.21958500	-0.85395800	0.35949800
C	2.03015900	-1.88034100	0.03954700
H	1.61439900	-0.14369500	1.09862100
H	1.68696600	-2.63337300	-0.67641600
C	3.40269300	-2.13623400	0.52196100
C	4.01924000	-3.36837900	0.25904100
C	4.16664800	-1.17382200	1.21587700
C	5.32262200	-3.65500100	0.67278500
H	3.46362600	-4.13049600	-0.28387700
C	5.46302800	-1.43894200	1.63152000
H	3.74111200	-0.19401600	1.40941500
C	6.05386700	-2.68489500	1.36657700
H	5.75084300	-4.62507400	0.44659600
H	6.04914600	-0.69223700	2.15908400
O	7.33613900	-2.84170900	1.81705600
C	7.98386900	-4.07644700	1.56556200
H	8.98147100	-3.98858200	2.00114400
H	7.45569100	-4.91635600	2.03865300
H	8.07588000	-4.27519300	0.48838300
C	-1.13227000	-2.76119200	-0.84380000
C	-2.23205400	-2.94461900	-1.74655400
C	-3.48212800	-3.46899200	-1.32767600
C	-2.08275100	-2.62492000	-3.12075800
C	-4.51933600	-3.65802100	-2.23376100
H	-3.63573900	-3.72489100	-0.28471400
C	-3.11796900	-2.82456000	-4.02248100
H	-1.14425100	-2.19575100	-3.45639800
C	-4.34500100	-3.33862600	-3.58558100
H	-5.46735400	-4.06297300	-1.88849400
H	-2.97806300	-2.56559100	-5.06832300
H	-5.15789000	-3.48742900	-4.29119600
C	-1.13460900	-3.33844800	0.54209000
H	-0.23945100	-3.03099600	1.08454000
H	-2.01838100	-3.03171800	1.11378700
H	-1.15755900	-4.43991500	0.50251600
H	-0.16642400	-2.59456400	-1.30825900
Br	-2.03898400	0.56242800	-2.14637500

TS3-*S*

C	3.69301300	3.47518800	-0.64044600
C	2.87608200	2.25666700	-0.17715000
C	3.92036200	2.94410700	0.72701600
H	4.43656200	3.27539700	-1.40339000
H	3.13306400	4.39676300	-0.75072200
H	3.51486200	3.48957400	1.57189700
H	4.82198000	2.37165600	0.91641400
C	1.45774900	2.49811700	0.16155300
C	-0.19782200	3.78491000	1.02321800
C	-0.73611800	2.42898700	0.49739800
H	-0.19403500	3.87388400	2.11195800
H	-1.26959600	1.86585300	1.26365500
C	3.25586800	0.94044400	-0.73613700

C	4.78422300	-0.52787400	-1.51229000
C	3.35974000	-1.15431800	-1.50721500
H	5.25528700	-0.49973600	-2.49548600
H	2.93117000	-1.36118900	-2.49015000
O	1.20101500	3.75145500	0.60763900
O	4.55551700	0.85508800	-1.12218100
N	0.47562100	1.67761900	0.10248900
N	2.52177600	-0.09744700	-0.88128300
C	-0.96701000	4.90318800	0.29980900
C	5.62889000	-1.28380400	-0.45721800
C	-1.58860300	2.79807900	-0.69582600
C	-2.17483900	1.93858700	-1.62485000
C	-1.69215000	4.18723200	-0.82118900
C	-2.89000200	2.49917900	-2.68584600
H	-2.05641000	0.86300500	-1.53653500
C	-2.40870600	4.74273800	-1.88065000
C	-3.00873900	3.88890200	-2.81085000
H	-3.34940800	1.84773300	-3.42369900
H	-2.49507500	5.82149100	-1.98718600
H	-3.56639400	4.30904500	-3.64383700
H	-1.66980100	5.38357500	0.99325700
H	-0.28108100	5.68259900	-0.05073300
C	3.49787000	-2.39400700	-0.65732800
C	2.55069200	-3.39721400	-0.45393200
C	4.75389000	-2.44848200	-0.04481900
C	2.87091500	-4.45234700	0.40420000
H	1.58905000	-3.33620300	-0.95520500
C	5.07262500	-3.50450000	0.80862100
C	4.11978800	-4.50232000	1.03538700
H	2.14435800	-5.24050700	0.58090700
H	6.04766700	-3.55660900	1.28717600
H	4.35576300	-5.32973300	1.69947500
H	6.58988200	-1.61358800	-0.86897500
H	5.85648300	-0.61339800	0.38120700
Ni	0.42576000	-0.37968900	-0.47077600
C	-1.27355900	-0.77182900	0.38256000
C	-2.17501400	-1.65809200	-0.08327500
H	-1.60953400	0.00650800	1.06209400
H	-1.83287500	-2.44363500	-0.75318900
C	-3.61979700	-1.62129300	0.16580100
C	-4.47489600	-2.40903400	-0.62223700
C	-4.22055200	-0.82454700	1.16598300
C	-5.85975900	-2.40649900	-0.44667700
H	-4.04381200	-3.03327800	-1.40127700
C	-5.59317900	-0.81021300	1.35276100
H	-3.59706800	-0.21535500	1.81475200
C	-6.42851900	-1.60004600	0.54549700
H	-6.47622200	-3.03032000	-1.08396400
H	-6.05153000	-0.20036000	2.12546600
O	-7.76582900	-1.51305500	0.81439500
C	-8.65554000	-2.29021100	0.03105200
H	-9.65749100	-2.07125700	0.40607000
H	-8.60039900	-2.02080800	-1.03297500
H	-8.45639400	-3.36582400	0.13753800
C	0.48889000	-1.39203200	1.45009400
H	1.49064700	-1.55089700	1.04096500
C	0.55803400	-0.36160700	2.52330000
C	1.71927700	0.42047300	2.66771800
C	-0.51852800	-0.09739600	3.39023400
C	1.79408700	1.44464200	3.60801800
H	2.56106300	0.22613600	2.00850700

C	-0.44964600	0.93221800	4.33132400
H	-1.41946500	-0.69993100	3.32367900
C	0.70198600	1.71548200	4.43995900
H	2.70509100	2.03203200	3.69510200
H	-1.29822800	1.11971600	4.98452600
H	0.75581600	2.51402900	5.17517200
C	-0.06050700	-2.76299600	1.82618000
H	-0.15124900	-3.38671100	0.93307800
H	0.63863300	-3.25577600	2.51691300
H	-1.04422100	-2.71641400	2.29845600
Br	0.13717800	-1.58531800	-2.63128400

TS3-*S*-cf2

C	-2.89739700	4.22721900	-1.47020700
C	-2.20981200	2.89088400	-1.17188100
C	-3.01969300	3.13035500	-2.46483400
H	-3.75603200	4.46049800	-0.85300000
H	-2.23774500	5.05614600	-1.69585300
H	-2.44138700	3.18603400	-3.38205900
H	-3.95751800	2.58843100	-2.53598900
C	-0.73637200	2.84988600	-1.30260700
C	1.26192000	3.72151800	-1.92966900
C	1.43185600	2.41222500	-1.11350900
H	1.50400100	3.63716300	-2.99240400
H	2.05028600	1.66450800	-1.61186900
C	-2.83676200	2.01614700	-0.14575000
C	-4.57168800	1.46317700	1.19924400
C	-3.38913200	0.45827300	1.35751200
H	-4.80127100	2.03092800	2.10261800
H	-2.92078600	0.43383100	2.34357500
O	-0.17719200	3.95804500	-1.84554200
O	-4.07600200	2.42357600	0.22572800
N	0.05050300	1.90012700	-0.95414500
N	-2.36549600	0.95718400	0.39599000
C	2.04043500	4.82875800	-1.20017900
C	-5.78754600	0.69136000	0.63905400
C	2.01914000	2.87164800	0.20406900
C	2.20669900	2.11728600	1.36143200
C	2.33076000	4.23430600	0.16189700
C	2.72611000	2.75671400	2.48948100
H	1.92341600	1.07061500	1.39790500
C	2.85435300	4.86746100	1.28843400
C	3.05067500	4.11836800	2.45250100
H	2.86575900	2.18950100	3.40513900
H	3.09963600	5.92660400	1.26624000
H	3.45177000	4.60100900	3.33997900
H	2.96516700	5.05622400	-1.74704500
H	1.45531400	5.75426900	-1.16105100
C	-3.98161700	-0.87068500	0.95341200
C	-3.35917300	-2.11501200	0.98328800
C	-5.29123500	-0.72904600	0.48457600
C	-4.04421300	-3.22299400	0.48016000
H	-2.35711900	-2.19945600	1.38214400
C	-5.98279900	-1.83702400	-0.00469700
C	-5.34510100	-3.08201000	-0.01707800
H	-3.55175200	-4.19064100	0.46314000
H	-7.00225900	-1.73736800	-0.37019100
H	-5.86951800	-3.94989500	-0.40870700
H	-6.63931400	0.74698500	1.32831700
H	-6.11392400	1.14289800	-0.30552100
Ni	-0.39367400	-0.02339300	0.03594900

C	1.23964400	-0.77853200	-0.68959600
C	2.10209600	-1.52012700	0.03201000
H	1.61113000	-0.24898900	-1.56455900
H	1.74414200	-2.03257200	0.91882800
C	3.54944300	-1.59267500	-0.20006500
C	4.39357000	-1.98885700	0.84988500
C	4.15998300	-1.26863600	-1.43088200
C	5.78067200	-2.04325100	0.70550400
H	3.95231400	-2.24984500	1.80882000
C	5.53593200	-1.31693900	-1.59048100
H	3.54105600	-0.99708400	-2.28179300
C	6.36143200	-1.70071300	-0.52114000
H	6.38984200	-2.35068700	1.54787100
H	6.00330900	-1.07590600	-2.54048400
O	7.70231200	-1.71595200	-0.78462600
C	8.58241800	-2.10871800	0.25504200
H	9.58896600	-2.05174700	-0.16453500
H	8.51282300	-1.43605800	1.12131900
H	8.38584400	-3.13819700	0.58559900
C	-0.54069500	-1.50446700	-1.57300400
H	0.16019300	-1.46196700	-2.40846700
C	-0.71253300	-2.95930700	-1.18811500
C	-1.06919800	-3.86676800	-2.20141000
C	-0.53320500	-3.46093000	0.10543300
C	-1.26274000	-5.22031100	-1.92736400
H	-1.19420000	-3.50970000	-3.22146700
C	-0.72308000	-4.81660600	0.38462900
H	-0.26751600	-2.78159600	0.90624500
C	-1.09312100	-5.70319300	-0.62721700
H	-1.54013700	-5.89822700	-2.73088600
H	-0.58048200	-5.17384400	1.40138100
H	-1.23969600	-6.75807800	-0.40981400
C	-1.84614600	-0.86747500	-2.10495600
H	-1.69563000	0.18052300	-2.38450900
H	-2.15452900	-1.40839700	-3.01217300
H	-2.66378200	-0.93059600	-1.38780500
Br	-0.25568000	-0.60870500	2.48313100

TS3-*S*-cf3

C	1.60857000	-2.54903300	1.37114900
C	0.45608400	-1.58616000	1.73603300
C	0.92419800	-2.69686000	2.68052600
H	1.39308400	-3.25793300	0.57879500
H	2.59555800	-2.10101300	1.32391400
H	1.43953300	-2.36367100	3.57377200
H	0.23480600	-3.52211700	2.81399800
C	0.84843900	-0.19497600	2.06413500
C	2.11495300	1.34816100	3.11904800
C	1.11042400	2.02252300	2.12996200
H	1.98589300	1.64637100	4.16034700
H	0.34693000	2.65230700	2.59502400
C	-0.82874700	-1.78209800	1.02153600
C	-2.52079900	-3.06716500	0.23160000
C	-2.74840700	-1.56535900	-0.08143500
H	-2.24566200	-3.67713000	-0.63334700
H	-2.93456300	-1.36100900	-1.13283400
O	1.77557800	-0.06607900	3.04881300
O	-1.34984600	-3.03018200	1.10846800
N	0.43043400	0.87276600	1.50354000
N	-1.48158100	-0.92305900	0.32670500
C	3.54341800	1.60966400	2.58521400

C	-3.75393200	-3.57872100	0.99076500
C	1.98903100	2.78958000	1.16722000
C	1.58452900	3.61896800	0.12119500
C	3.34263300	2.54398700	1.41295700
C	2.56416500	4.18922100	-0.69684300
H	0.52693200	3.79343900	-0.05257900
C	4.31917300	3.12546900	0.60331000
C	3.92182100	3.94288300	-0.45941900
H	2.26699300	4.83181600	-1.52110000
H	5.37480500	2.94305600	0.79101600
H	4.67360100	4.39446300	-1.10119500
H	4.19200100	2.04169700	3.35642100
H	3.99745800	0.66011800	2.27506300
C	-3.91607700	-1.18392000	0.80300000
C	-4.42212200	0.09220300	1.04322400
C	-4.46931500	-2.31331400	1.41466300
C	-5.51027700	0.22010400	1.91019400
H	-3.95305900	0.96497900	0.59953100
C	-5.55747400	-2.18243000	2.27697100
C	-6.07604400	-0.90719200	2.51919900
H	-5.91424300	1.20699600	2.11895100
H	-5.99297200	-3.05529100	2.75774700
H	-6.92140500	-0.78960800	3.19241000
H	-4.37570300	-4.18823500	0.32151500
H	-3.45979700	-4.22051300	1.82873700
Ni	-0.60763200	0.90738200	-0.43140800
C	0.75665700	-0.11212200	-1.37031700
C	2.06214200	0.20656700	-1.29386200
H	0.44153900	-1.13633400	-1.57876300
H	2.34412900	1.23946600	-1.09828800
C	3.16677200	-0.76356400	-1.28685200
C	4.39369300	-0.41232100	-0.70032000
C	3.04760000	-2.07930400	-1.78528500
C	5.44663700	-1.32336400	-0.57772500
H	4.52334400	0.59894300	-0.32468400
C	4.08317200	-2.99422800	-1.67229700
H	2.12750400	-2.38372400	-2.27664000
C	5.29290500	-2.62833500	-1.05910500
H	6.37268100	-1.00322500	-0.11319200
H	3.98869600	-4.00361800	-2.06136000
O	6.24436800	-3.60754400	-0.99311000
C	7.49245400	-3.28591300	-0.40344500
H	8.09480800	-4.19502200	-0.45700900
H	8.00349800	-2.48041900	-0.94922300
H	7.37968800	-2.98848100	0.64878200
C	-0.62444500	0.98065600	-2.69355300
H	0.10413900	0.45654400	-3.30561700
C	-1.95964500	0.36373000	-2.91852400
C	-3.17126100	0.98607900	-2.55725900
C	-2.02547400	-0.90869900	-3.52157900
C	-4.39181900	0.35262100	-2.78310100
H	-3.14412200	1.94508400	-2.05254700
C	-3.24829100	-1.54142700	-3.74780300
H	-1.10168700	-1.40160000	-3.81762000
C	-4.44050900	-0.91355400	-3.37624900
H	-5.31137900	0.84635700	-2.48081600
H	-3.27017400	-2.52141200	-4.21842600
H	-5.39543100	-1.40328900	-3.54651500
C	-0.43061000	2.47816000	-2.88724500
H	-0.54848000	2.72445500	-3.95315500
H	-1.13123600	3.07842300	-2.30603900

H	0.58436600	2.76127400	-2.58680700
Br	-2.00361500	2.85830100	0.30308500

TS3-*R*

C	4.83539800	-0.06231100	1.96008100
C	3.42062200	-0.08523600	1.37450400
C	3.75071000	-0.69697900	2.75256000
H	5.55674300	-0.70154300	1.46516500
H	5.19929100	0.90961500	2.27162000
H	3.34909600	-0.16807600	3.61103100
H	3.70560600	-1.78036200	2.80809000
C	2.67159200	1.19678900	1.36864300
C	2.23540200	3.28681300	2.13504700
C	1.33770700	2.91643100	0.92095300
H	1.71828600	3.32121600	3.09753200
H	0.26757200	3.03479000	1.10777000
C	3.15207300	-1.03188000	0.25989400
C	3.67326500	-2.73899900	-1.11892100
C	2.31015400	-2.10070400	-1.51119800
H	4.42201000	-2.71347200	-1.91123300
H	2.30482700	-1.53486900	-2.44683000
O	3.15787800	2.15196700	2.19867600
O	4.17084000	-1.87158100	-0.05615000
N	1.63145900	1.49130500	0.68058500
N	2.07608300	-1.12379000	-0.42435600
C	2.98431100	4.58125700	1.78256900
C	3.37588700	-4.17382700	-0.61909600
C	1.84463500	3.80671900	-0.19337700
C	1.50398700	3.75275300	-1.54395000
C	2.78391500	4.72375400	0.28857100
C	2.11891200	4.65616100	-2.41459700
H	0.81493100	2.99840700	-1.91409600
C	3.39328100	5.62488200	-0.58380800
C	3.05170600	5.58635800	-1.93876500
H	1.87555300	4.62703900	-3.47293900
H	4.12551400	6.34156400	-0.21963900
H	3.52170100	6.28040000	-2.63076500
H	2.54727000	5.42570300	2.33192400
H	4.03649500	4.51565100	2.08097900
C	1.34258000	-3.25512200	-1.53441900
C	0.02288300	-3.24892800	-1.98335500
C	1.94343600	-4.42089800	-1.04824900
C	-0.69617400	-4.44592200	-1.94327900
H	-0.41584200	-2.32820200	-2.35803600
C	1.22501000	-5.61596300	-1.01695200
C	-0.09861200	-5.61983900	-1.46965400
H	-1.72661100	-4.46586400	-2.28643700
H	1.68529400	-6.53197000	-0.65404200
H	-0.66901900	-6.54487100	-1.45143900
H	4.07610500	-4.90638800	-1.03596200
H	3.48846100	-4.21130600	0.47244300
Ni	0.27450100	0.06457800	-0.23961900
C	-1.44634600	0.67227100	0.38217800
C	-2.60835800	0.09474800	0.02235800
H	-1.42958000	1.69473600	0.76332200
H	-2.58824200	-0.96102300	-0.24730300
C	-3.93541600	0.71335300	-0.02553400
C	-5.06961300	-0.10004700	-0.17888300
C	-4.15126000	2.10372300	0.09109500
C	-6.36336600	0.42506500	-0.19913300
H	-4.93631300	-1.17541800	-0.27374000

C	-5.42839500	2.64037200	0.07433300
H	-3.29954700	2.77241200	0.17871700
C	-6.54911400	1.80561900	-0.06917400
H	-7.20685000	-0.24591100	-0.31677700
H	-5.59156700	3.71060000	0.15804300
O	-7.76190200	2.43807700	-0.07502700
C	-8.92472300	1.64683800	-0.24479900
H	-9.76752000	2.34118300	-0.22947700
H	-8.91469200	1.11099600	-1.20438800
H	-9.04279700	0.91751700	0.56925500
C	-0.36751400	-0.53696300	1.84984500
C	-1.07281300	-1.84490000	1.90449200
C	-2.40893200	-1.96567600	2.32882900
C	-0.38572400	-3.01883400	1.55088900
C	-3.02676300	-3.21361400	2.40229900
H	-2.97272700	-1.07641100	2.58928200
C	-0.99859400	-4.26762900	1.62994000
H	0.63881900	-2.94402700	1.19798100
C	-2.32485600	-4.37093300	2.05573200
H	-4.06043500	-3.28093000	2.73237000
H	-0.44158800	-5.15413900	1.34514300
H	-2.80726100	-5.34311200	2.11546500
C	-0.67607200	0.45426800	2.96469400
H	-1.72929200	0.74528500	2.99840900
H	-0.41951600	0.00946400	3.93688500
H	-0.08188800	1.36572800	2.84493600
H	0.70992300	-0.72205600	1.85322900
Br	0.14901400	0.43718100	-2.69357000

TS3-*R*-cf2

C	4.43857400	2.96426800	0.15677300
C	3.37114100	1.87788600	0.37296000
C	4.39798100	2.25552700	1.46056500
H	5.22419700	2.70916400	-0.54490900
H	4.06877600	3.98177200	0.10838100
H	3.99643800	2.77779200	2.32268300
H	5.15359400	1.50567300	1.66898100
C	1.98151900	2.34334300	0.58561100
C	0.45378300	3.87631500	1.27157900
C	-0.22378000	2.63294600	0.63725400
H	0.32593200	3.96163300	2.35378200
H	-0.95454400	2.16115700	1.29651000
C	3.58335600	0.59080000	-0.33179500
C	4.90160400	-1.01478300	-1.22279300
C	3.39437500	-1.35255500	-1.41956400
H	5.46244200	-0.91419200	-2.15274600
H	3.03499900	-1.34154300	-2.45103100
O	1.87414000	3.61629900	1.04045800
O	4.88385800	0.30712100	-0.60824300
N	0.89232200	1.69276300	0.40055900
N	2.70070200	-0.25342500	-0.70497900
C	-0.01827700	5.11301100	0.48944900
C	5.49587400	-2.07893900	-0.26797100
C	-0.81316000	3.14800400	-0.65805900
C	-1.37774000	2.40497500	-1.69484100
C	-0.67271800	4.53675400	-0.74895000
C	-1.82140800	3.08646500	-2.83073900
H	-1.43314300	1.32191400	-1.64570000
C	-1.12128200	5.21335100	-1.88244100
C	-1.69949400	4.47828800	-2.92166900
H	-2.25124600	2.52367700	-3.65408800

H	-1.01653700	6.29276100	-1.96232200
H	-2.04750000	4.99237300	-3.81383200
H	-0.73192500	5.68726800	1.09499000
H	0.82394300	5.77892400	0.27055200
C	3.21253200	-2.69084300	-0.74625400
C	2.05843500	-3.47431000	-0.71191400
C	4.37162700	-3.07312500	-0.06483000
C	2.07528100	-4.64952800	0.04323500
H	1.17616000	-3.15399200	-1.25877900
C	4.38658100	-4.24965200	0.68465000
C	3.22874000	-5.03134800	0.74024000
H	1.18679300	-5.27371700	0.08705100
H	5.28264600	-4.55637100	1.21888600
H	3.22721700	-5.94806900	1.32415200
H	6.38846400	-2.54904900	-0.69767700
H	5.80813800	-1.60196000	0.66924900
Ni	0.58434700	-0.27805000	-0.35984400
C	-1.22083300	-0.54730800	0.30921300
C	-2.05372500	-1.50483300	-0.15440100
H	-1.64671600	0.34776600	0.75671500
H	-1.61869500	-2.39843100	-0.59889400
C	-3.51224000	-1.41175600	-0.19900200
C	-4.23830400	-2.22655900	-1.08450900
C	-4.25212700	-0.53771200	0.62974800
C	-5.62974800	-2.17208800	-1.16842400
H	-3.69614600	-2.90803100	-1.73567000
C	-5.63479900	-0.47371500	0.55693700
H	-3.73349400	0.07645400	1.35943300
C	-6.33791000	-1.28702800	-0.34605000
H	-6.14411800	-2.81579500	-1.87299900
H	-6.20238600	0.19288700	1.19928500
O	-7.69812500	-1.14604100	-0.33597200
C	-8.45757000	-1.93852400	-1.23225900
H	-9.50191300	-1.66633800	-1.06516100
H	-8.19335400	-1.73375700	-2.27921000
H	-8.32803100	-3.01218000	-1.03546200
C	0.13812400	-1.23442500	1.72532600
C	-0.72379000	-0.65805200	2.80860800
C	-0.39373300	0.55573100	3.43688500
C	-1.90375100	-1.30589700	3.21323800
C	-1.21511800	1.10391600	4.42351000
H	0.51069500	1.08013000	3.14419700
C	-2.72040500	-0.76641200	4.20545000
H	-2.19276300	-2.22792900	2.71704600
C	-2.38343200	0.44612700	4.81395400
H	-0.93838100	2.04574600	4.89181200
H	-3.62812300	-1.28885100	4.49559900
H	-3.02322700	0.87177700	5.58214700
C	1.63385500	-1.14848500	2.08596600
H	2.02259100	-0.12789500	2.08838500
H	1.76349500	-1.55237000	3.10179500
H	2.24334800	-1.74146100	1.40717900
H	-0.11552600	-2.27493700	1.52977100
Br	0.21479500	-0.98657100	-2.75061600

TS3-*R*-cf3

C	0.67802800	-3.36424500	0.30548600
C	-0.45266800	-2.44665000	0.82329600
C	-0.28022500	-3.88784100	1.31137200
H	0.58820200	-3.68251700	-0.72756200
H	1.68177000	-3.08418800	0.60791300

H	0.05309900	-3.99525300	2.33692400
H	-1.03961500	-4.58939500	0.98678000
C	-0.04226100	-1.35572100	1.73933700
C	1.02847400	-0.53305200	3.55100400
C	0.37018200	0.61113300	2.71458000
H	0.64544300	-0.62168900	4.56864700
H	-0.42654900	1.16349300	3.22017500
C	-1.56734800	-2.16201100	-0.11069900
C	-3.14829800	-2.78624800	-1.61148100
C	-3.20170800	-1.26275000	-1.31468400
H	-2.74762600	-3.05158200	-2.59403800
H	-3.16281400	-0.63874800	-2.20995400
O	0.62679300	-1.74807600	2.85318600
O	-2.18194600	-3.25740900	-0.62231500
N	-0.23005400	-0.10467700	1.57204500
N	-2.00061000	-1.01531700	-0.49296500
C	2.56125100	-0.33587200	3.49262000
C	-4.54210900	-3.36556500	-1.32927700
C	1.52733800	1.49408700	2.30757500
C	1.47383200	2.68096500	1.57749900
C	2.74736500	0.95062500	2.71883800
C	2.67362900	3.31445800	1.24351600
H	0.51724300	3.08583500	1.26131200
C	3.94394700	1.59181000	2.39635300
C	3.89923600	2.77237100	1.64797600
H	2.64783800	4.22358900	0.65334000
H	4.89784300	1.17837300	2.71630200
H	4.82517300	3.27460900	1.38012500
H	2.99745000	-0.28182000	4.49739100
H	3.02281100	-1.19288600	2.98656300
C	-4.48964900	-1.09266500	-0.53500600
C	-4.92903100	0.04111500	0.14630000
C	-5.22943300	-2.27971900	-0.52928100
C	-6.14570200	-0.02967500	0.82973400
H	-4.31798400	0.93876500	0.18069300
C	-6.44384600	-2.34548700	0.15267900
C	-6.89878700	-1.21047700	0.82987800
H	-6.50346600	0.83967200	1.37440600
H	-7.02456400	-3.26476500	0.16447400
H	-7.84180800	-1.24860700	1.36910300
H	-5.06245600	-3.56163400	-2.27635500
H	-4.46769200	-4.32198200	-0.80012400
Ni	-0.81363800	0.82244300	-0.30445500
C	0.56866000	0.01088100	-1.38804700
C	1.85613000	0.08275200	-1.00429600
H	0.20906700	-0.82334800	-1.99268100
H	2.18302500	0.94165200	-0.42129000
C	2.84390000	-0.99192900	-1.16986300
C	3.92364500	-1.08142500	-0.27610400
C	2.73617300	-1.99967600	-2.15249200
C	4.83854100	-2.13691400	-0.32308100
H	4.04147700	-0.31102100	0.48120200
C	3.63748900	-3.05185700	-2.21316100
H	1.93536900	-1.94774900	-2.88530300
C	4.69488100	-3.13646300	-1.29241500
H	5.65269400	-2.16403600	0.39265800
H	3.55520500	-3.82221100	-2.97405300
O	5.52063100	-4.21652700	-1.43532100
C	6.61865300	-4.33860800	-0.54741100
H	7.14038100	-5.25313900	-0.83661100
H	7.30710700	-3.48633100	-0.63385600

H	6.28826100	-4.42597000	0.49737500
C	-0.35258600	1.75059200	-2.31844200
H	0.09851600	1.17612400	-3.12570100
Br	-2.20814600	2.51441200	0.88022800
C	0.48380800	2.96056000	-2.06102500
C	1.82004400	2.98102000	-2.50439500
C	-0.02564300	4.11865900	-1.44668100
C	2.61316600	4.11747800	-2.35805400
H	2.23401800	2.09220500	-2.97248300
C	0.76755200	5.25729100	-1.30279500
H	-1.03684900	4.11690200	-1.05824400
C	2.08738500	5.26735700	-1.76273300
H	3.63936600	4.10771400	-2.71676500
H	0.34833100	6.14163400	-0.82967000
H	2.69947800	6.15949600	-1.65627200
C	-1.82012300	1.98835600	-2.69192200
H	-1.85601300	2.60690100	-3.60203600
H	-2.31254600	1.03876300	-2.92192700
H	-2.39475400	2.48641500	-1.90980800

TS4-*S*

C	3.23208300	3.56651500	-0.17821100
C	2.55391200	2.20444100	0.07091700
C	3.51707700	2.84573900	1.08674200
H	3.98959300	3.57442900	-0.95313900
H	2.57258900	4.42613900	-0.14592700
H	3.05250600	3.19710200	2.00103200
H	4.47586200	2.35053700	1.19817400
C	1.11377100	2.24595700	0.38011200
C	-0.71817100	3.20386400	1.30393600
C	-1.09123600	2.00074700	0.40711500
H	-0.79969400	3.01482900	2.37595600
H	-1.72930900	1.27068400	0.90054000
C	3.06518200	1.04870000	-0.69427900
C	4.66968200	0.02202600	-1.91729100
C	3.43259300	-0.90272600	-1.72229800
H	4.80580900	0.38580700	-2.93836800
H	2.95499100	-1.23593900	-2.64382500
O	0.70893600	3.36358700	1.01776000
O	4.34442400	1.18830900	-1.10469400
N	0.21487000	1.38084500	0.07243100
N	2.45476800	-0.04007100	-0.99375300
C	-1.50207400	4.42061900	0.79630200
C	5.90451400	-0.69422700	-1.34678500
C	-1.73461600	2.64154600	-0.80555100
C	-2.06259100	2.04116500	-2.02019700
C	-1.93972200	4.00923100	-0.59366300
C	-2.61770200	2.83633400	-3.02612600
H	-1.88337400	0.98193500	-2.16772200
C	-2.49513500	4.80004800	-1.59856200
C	-2.83416300	4.20392400	-2.81673100
H	-2.88079500	2.38837800	-3.98043500
H	-2.65699100	5.86382100	-1.44165900
H	-3.26490800	4.80923400	-3.61008000
H	-2.36368000	4.60448100	1.45203600
H	-0.88417400	5.32515700	0.81655300
C	3.96863200	-2.05511900	-0.89514600
C	3.27947800	-3.17638300	-0.43267900
C	5.33501800	-1.90043800	-0.63560600
C	3.96494500	-4.11490500	0.34199500
H	2.23407200	-3.30441600	-0.68591600

C	6.01952100	-2.83882000	0.13664600
C	5.32294400	-3.94264200	0.63529800
H	3.43697400	-4.98767000	0.71637200
H	7.08098500	-2.71918800	0.34027800
H	5.84331300	-4.67958600	1.24143400
H	6.57823700	-0.99754700	-2.15839000
H	6.47274900	-0.02046900	-0.69533900
Ni	0.44027400	-0.49214000	-0.69145900
C	-1.35322000	-0.91633000	-0.21843700
C	-2.36855400	-1.37681600	-0.96268000
H	-1.53956800	-0.68850300	0.83398800
H	-2.17617100	-1.63860200	-2.00184000
C	-3.75998400	-1.57751300	-0.51671800
C	-4.70841100	-2.07799800	-1.42084900
C	-4.21420700	-1.28694300	0.78805400
C	-6.04305900	-2.28862400	-1.06413100
H	-4.39343300	-2.31452300	-2.43475900
C	-5.53379000	-1.49004800	1.16024100
H	-3.51774400	-0.89510200	1.52468700
C	-6.46359500	-1.99364800	0.23626400
H	-6.73418700	-2.67951800	-1.80249400
H	-5.87762200	-1.26608900	2.16581500
O	-7.73934400	-2.15483200	0.70589600
C	-8.71530600	-2.66469300	-0.18503700
H	-9.64665700	-2.71538600	0.38321800
H	-8.85725500	-2.00568100	-1.05341100
H	-8.45420500	-3.67118200	-0.54160500
C	0.94402300	-1.73548000	1.55048900
H	1.96024300	-1.73528800	1.16805400
C	0.65416500	-0.73047400	2.52700400
C	1.62530000	0.26729900	2.82041600
C	-0.58353200	-0.64627100	3.22144200
C	1.37209600	1.28469500	3.72823600
H	2.58407700	0.22008600	2.30974400
C	-0.83628000	0.38463000	4.12167600
H	-1.34324300	-1.40238500	3.05368600
C	0.13319500	1.36042500	4.38374000
H	2.14142800	2.02310300	3.94065400
H	-1.79422700	0.42232600	4.63471300
H	-0.06293000	2.15391200	5.09962500
C	0.19776500	-3.02887900	1.40754300
H	0.26571900	-3.39825400	0.38110300
H	0.62499300	-3.79140100	2.07995300
H	-0.86582200	-2.93708900	1.63995500
Cl	0.51419300	-2.14139600	-2.26255300

TS4-*R*

C	4.04933100	3.12538100	1.27529700
C	3.06849300	1.95975600	1.05542800
C	3.97254100	2.05562000	2.30067500
H	4.90526300	3.14363200	0.61092700
H	3.59313500	4.08443400	1.49116600
H	3.45822500	2.26281100	3.23331400
H	4.77414900	1.32648000	2.35327700
C	1.63589200	2.26096300	1.23724400
C	-0.09550500	3.43748400	2.11060000
C	-0.56063800	2.47560500	0.98961000
H	-0.36883700	3.13619200	3.12532900
H	-1.39677900	1.83097400	1.25987200
C	3.43702200	0.95398700	0.03153900
C	4.96489000	-0.18572300	-1.18220900

C	3.51832000	-0.64131300	-1.54156700
H	5.51435200	0.24808700	-2.01883700
H	3.19334400	-0.40563900	-2.55624800
O	1.36428600	3.33015600	2.01956100
O	4.76455400	0.88863400	-0.22054900
N	0.63672900	1.64993500	0.71247500
N	2.66546100	0.16088800	-0.61677700
C	-0.55236100	4.85081400	1.72618900
C	5.69422100	-1.38366300	-0.53319400
C	-0.87090700	3.40320600	-0.16906400
C	-1.09958600	3.06073800	-1.50152400
C	-0.84146300	4.73937600	0.24413500
C	-1.32123900	4.09021200	-2.41995000
H	-1.08471000	2.02173600	-1.81241500
C	-1.06626900	5.76351900	-0.67460900
C	-1.30963800	5.42919700	-2.01002200
H	-1.49680600	3.84593100	-3.46385900
H	-1.04448400	6.80466500	-0.36168600
H	-1.48199800	6.21740000	-2.73830500
H	-1.45324100	5.11441200	2.29648000
H	0.21393300	5.59368500	1.97354600
C	3.51911700	-2.12893400	-1.26498700
C	2.51451300	-3.05627700	-1.54783100
C	4.71719900	-2.53038000	-0.66546600
C	2.71136200	-4.38952800	-1.18068000
H	1.60006200	-2.72638700	-2.02986000
C	4.91290200	-3.86289200	-0.30211300
C	3.89776200	-4.78929900	-0.55413900
H	1.93387800	-5.12120000	-1.38194200
H	5.84309400	-4.17875400	0.16412800
H	4.03604600	-5.82981300	-0.27249400
H	6.64414600	-1.59541000	-1.03818100
H	5.93559900	-1.14898300	0.51092800
Ni	0.59428100	0.04388500	-0.53437800
C	-1.26358700	-0.23003600	-0.19873100
C	-2.29302900	-0.35155700	-1.04972600
H	-1.46475600	-0.28733800	0.87669600
H	-2.09740600	-0.28710800	-2.11858700
C	-3.70465300	-0.57052200	-0.68387800
C	-4.71294500	-0.35053100	-1.63338300
C	-4.10903900	-1.01772500	0.59157700
C	-6.06662800	-0.53847800	-1.33953300
H	-4.43367900	-0.01853300	-2.63107900
C	-5.44685200	-1.21152200	0.89924100
H	-3.35794600	-1.24590300	1.34009600
C	-6.44032300	-0.96925300	-0.06258300
H	-6.80802900	-0.35066600	-2.10823800
H	-5.75278600	-1.56810500	1.87856600
O	-7.72973700	-1.19360800	0.34456300
C	-8.76762800	-0.98349400	-0.59481100
H	-9.69914800	-1.21976100	-0.07542700
H	-8.80115200	0.06026800	-0.93886200
H	-8.66495500	-1.64160300	-1.46953200
C	0.94623400	-1.79843300	1.32873700
C	-0.26221600	-2.54276400	1.53836500
C	-1.00842000	-2.46014100	2.74020600
C	-0.76345600	-3.38671700	0.51319200
C	-2.17499000	-3.20207200	2.91403700
H	-0.65990800	-1.82136000	3.54644700
C	-1.93124400	-4.11295300	0.68728900
H	-0.23051600	-3.42573400	-0.43181400

C	-2.64477200	-4.03174500	1.89213400
H	-2.72503400	-3.12787100	3.84904000
H	-2.30183900	-4.73818800	-0.12044700
H	-3.56383100	-4.59574000	2.02280000
C	1.59031600	-1.01557900	2.43498700
H	0.93761200	-0.21684900	2.81001100
H	1.83337500	-1.66079800	3.29448100
H	2.51915000	-0.55403200	2.09481700
H	1.59073000	-2.14468000	0.53059100
Cl	0.49161100	-0.53365900	-2.76197000

C_6F_6 (ORCA)

C	-2.08035480745116	-0.00000002082751	-1.58571637452826
C	-3.23769074609703	-0.35506623649578	-0.88678634286898
C	-0.92301885233540	0.35506620501065	-0.88678633289870
F	-4.34757496386184	-0.69560642286598	-1.55705193271356
F	0.18686535087026	0.69560647574205	-1.55705190979329
C	-3.23769072197718	-0.35506626792560	0.51107006734975
C	-0.92301888078694	0.35506626628939	0.51107007660397
F	-4.34757492081340	-0.69560645694073	1.18133567640156
F	0.18686527613664	0.69560653123027	1.18133569163507
C	-2.08035478998591	-0.00000002292387	1.21000011999390
F	-2.08035478968096	-0.00000002759632	2.55054875632649
F	-2.08035483801706	-0.00000002269656	-2.92626500750795

1b

C	-0.60660232044200	-0.64433995736758	-3.38005900922667
H	-1.60460746635295	-0.65333316008541	-2.93305121239365
C	0.45328983433487	-0.63003599455311	-2.55907618308245
H	1.49371646233862	-0.61917515448373	-2.87727047533960
C	-0.57195750470224	-0.64861128117865	-4.84562063603251
C	0.61998802181281	-0.64566072383544	-5.60004674907194
C	-1.79395229994265	-0.65623905033384	-5.54712355152329
C	0.59517064787864	-0.64949435993566	-6.99133310043017
C	-1.83877360137507	-0.66018199574130	-6.94122267777344
C	-0.63805521806712	-0.65660166165618	-7.64189706791389
H	1.58733125793119	-0.64084111737458	-5.09619226931526
H	-2.72959761049744	-0.65893299398530	-4.98438732683538
H	1.51428622431806	-0.64739411187043	-7.57720370736237
H	-2.78463544871623	-0.66600376119840	-7.48253959334636
F	-0.66382938969596	-0.66046915172425	-8.99889893159037
Br	0.28753780217748	-0.62612448467614	-0.66915781976260

5b

C	-4.11532733969400	-1.12491356946914	-6.01429941298077
H	-5.16322602983630	-1.18831310673920	-5.69956900909472
C	-3.16762685914312	-1.13905534301137	-5.03808195186337
H	-2.10787415739093	-1.07707981623321	-5.30788545720844
C	-3.91045792977807	-1.03391764636984	-7.45326825019406
C	-2.63517196350950	-0.94763086729187	-8.05638453755352
C	-5.03578889837609	-1.03059708961062	-8.30621104514578
C	-2.48924585320593	-0.86218979261019	-9.43718523379085
C	-4.90926778262523	-0.94565654158294	-9.69165106403804
C	-3.63208872736911	-0.86244512329972	-10.23619868915133
H	-1.73841991553155	-0.94745158212351	-7.43570092957264
H	-6.03294651870383	-1.09641924344777	-7.86583450438134
H	-1.50595499406426	-0.79571102427353	-9.90305274209395
H	-5.78036122167198	-0.94331164978294	-10.34674289253264
F	-3.49132406175592	-0.77912663157127	-11.58447816915054
C	-2.51469059029440	-1.24576756800706	-2.66099915450661
H	-1.46687926140760	-1.18133021087182	-2.97581356345228

C	-3.46240715910281	-1.23201887654516	-3.63721103848032
H	-4.52215243116646	-1.29415602968902	-3.36740962017595
C	-2.71942537499200	-1.33692986135368	-1.22203428823827
C	-3.99440254918885	-1.42969757739758	-0.61920347627904
C	-1.59432007195093	-1.33379279785311	-0.36880889762441
C	-4.14025890510305	-1.51467685159697	0.76162695209982
C	-1.72077805437147	-1.41813833281390	1.01667612550167
C	-2.99765036725211	-1.50753157476434	1.56095297035080
H	-4.89091017520987	-1.43587163754732	-1.24020875430780
H	-0.59740181833295	-1.26311777973175	-0.80897429389443
H	-5.12328860004276	-1.58630054772655	1.22728282564892
H	-0.84986377820378	-1.41537981423140	1.67200496308725
F	-3.13833900772507	-1.59044516645319	2.90926760902267

7b low spin (ORCA)

Ni	-0.00448447947817	0.00164020935805	0.00239302745841
Br	1.48051895103094	-1.06498553032033	-1.42227277162176
O	-2.14618384754168	2.70992481822853	2.28296153762120
O	2.37980000208120	1.45487800242610	3.14553552778769
N	-1.22320307906186	1.24516125844987	0.82001397365091
N	1.38187625645077	0.45142569725855	1.37268370607038
C	-2.50473685992759	1.69215636182839	0.20028167496512
H	-3.02703521838075	0.81954243180769	-0.21284803429169
C	-2.31414275829491	2.79291707793566	-0.82891167485076
C	-1.51232164190342	2.78354092305962	-1.97151518676158
H	-0.91049815291267	1.90705015537969	-2.21296422085385
C	-1.48667183784918	3.92329934054902	-2.78164286180547
H	-0.86400414733098	3.93421369639543	-3.67748163198082
C	-2.24859325449193	5.05089471093451	-2.45132495367893
H	-2.21699304159929	5.93275922013676	-3.09340087627570
C	-3.04370247594705	5.05993212985855	-1.30037856197006
H	-3.62841865175398	5.94527500807122	-1.04169202149398
C	-3.07032191595432	3.92462134312011	-0.48855181182245
C	-3.84960356218091	3.69579574776801	0.78758163630582
H	-4.92295813349767	3.54333522451468	0.58491826850845
H	-3.77594304049303	4.52241849465255	1.50957617890282
C	-3.24782198837848	2.40494676146387	1.34996429789153
H	-3.96181548987140	1.77984856842228	1.90421276940742
C	-1.09373624482642	1.93610966048983	1.91190877407963
C	0.08827487863875	2.02707353287255	2.78263295351118
C	0.30730255664132	3.36135515193982	3.52746537385254
H	1.34653079630200	3.67037733750601	3.61840043281037
H	-0.41234425523186	4.14546701019410	3.30285053411278
C	-0.14793530388374	2.18122249430214	4.30349516773825
H	-1.18964241399308	2.13532039477542	4.61554073715783
H	0.56860777104095	1.66629142186091	4.94026945293758
C	1.28394818055181	1.27319317310105	2.36563742651106
C	3.48191174327627	0.72368410573533	2.48920495801151
H	4.12422097702651	1.48580470131008	2.02662190639825
C	4.20274200542984	-0.17332887910662	3.49859326451102
H	5.27190389476995	-0.23974757835717	3.23630663075593
H	4.15016485558222	0.24321584348415	4.51544988063502
C	3.51466753766513	-1.51073413328666	3.34409244178004
C	3.63740994066102	-2.64517637862000	4.14876463050819
H	4.27231145295736	-2.63258666009796	5.03739448769345
C	2.93467878923394	-3.80286202606502	3.79970833654877
H	3.02093633812608	-4.69635418451876	4.42049587820722
C	2.12461490652130	-3.82420395829027	2.65754694487539
H	1.58689261393112	-4.73596305778894	2.39252422867949
C	1.99959201633266	-2.68990406879442	1.84907993232279
H	1.38061955905140	-2.70181397824566	0.95098959516874

C	2.69685644850178	-1.53271201153393	2.20372943294103
C	2.72663367802005	-0.18761704582790	1.49388927054575
H	3.16825451112758	-0.25449746077197	0.49010899285342
C	-1.40149009875006	-0.78861801427904	-0.96238725294244
H	-2.03510631596574	-1.28764677439133	-0.20524543892123
C	-1.75652521743068	-0.86523505167258	-2.25838195017227
H	-1.10501207355006	-0.40857308973478	-3.01166561912331
C	-2.95112365620622	-1.51914280490975	-2.81650139221344
C	-3.85728499891261	-2.28306114369508	-2.04982337266299
C	-3.22151252707195	-1.39017338112351	-4.19468295364675
C	-4.97922286924958	-2.87842821603045	-2.62231397369321
C	-4.33992211595134	-1.97850977092046	-4.78764052083420
C	-5.20574311712741	-2.71388056534712	-3.98709027461802
H	-3.67377801332535	-2.42693252758972	-0.98369370994579
H	-2.53258188490982	-0.81230109787859	-4.81459548781304
H	-5.67387381635059	-3.47346317041941	-2.02849829659437
H	-4.54200225250226	-1.87474016712144	-5.85400827132554
F	-6.30308490686389	-3.29527128245169	-4.55036414280337

10. References

[1] W. C. Still, M. Kahn, A. Mitra, *J. Org. Chem.* **1978**, *43*, 2923–2925.
[2] Stoll, S.; Schweiger, A. Easyspin, a comprehensive software package for spectral simulation and analysis in EPR. *J. Magn. Reson.* **2006**, *178*, 42–45.
[3] L. Hofstra, J. Synthesis of Chiral Bisoxazoline Ligands: (3aR,3a'R,8aS,8a'S)-2,2'-(Cyclopropane-1,1-Diyl)Bis(3a,8a-Dihydro-8H-Indeno[1,2-d]Oxazole). *Org. Synth.* **2020**, *97*, 172–188.
[4] Hofstra, J. L.; Cherney, A. H.; Ordner, C. M.; Reisman, S. E. Synthesis of Enantioenriched Allylic Silanes via Nickel-Catalyzed Reductive Cross-Coupling. *J. Am. Chem. Soc.* **2018**, *140* (1), 139–142.
[5] Suzuki, N.; Hofstra, J. L.; Poremba, K. E.; Reisman, S. E. Nickel-Catalyzed Enantioselective Cross-Coupling of N-Hydroxyphthalimide Esters with Vinyl Bromides. *Org. Lett.* **2017**, *19* (8), 2150–2153.
[6] Bull, J. A.; Mousseau, J. J.; Charette, A. B. Convenient One-Pot Synthesis of (E)-β-Aryl Vinyl Halides from Benzyl Bromides and Dihalomethanes. *Org. Lett.* **2008**, *10*, 5485–5488.
[7] Everson, D. A.; Jones, B. A.; Weix, D. J. Replacing Conventional Carbon Nucleophiles with Electrophiles: Nickel-Catalyzed Reductive Alkylation of Aryl Bromides and Chlorides. *J. Am. Chem. Soc.* **2012**, *134* (14), 6146–6159.
[8] Preliminary kinetic modeling studies done with COPASI software package: S. Hoops, S. Sahle, R. Gauges, C. Lee, J. Pahle, N. Simus, M. Singhal, L. Xu, P. Mendes, U. Kummer, *Bioinformatics* **2006**, *22*, 3067–3074.
[9] Jette, C.I.; Tong, Z.J.; Hadt, R.G.; Stoltz, B.M. Copper-catalyzed enantoselective allylic alkylation with a butyrolactone-derived silyl ketene acetal. *Angew. Chem. Int. Ed.* **2019**, *59*, 2033–2038.
[10] Frisch, M. J.; Trucks, G. W.; Schlegel, H. B.; Scuseria, G. E.; Robb, M. A.; Cheeseman, J. R.; Scalmani, G.; Barone, V.; Petersson, G. A.; Nakatsuji, H.; Li, X.; Caricato, M.; Marenich, A. V.; Bloino, J.; Janesko, B. G.; Gomperts, R.; Mennucci, B.; Hratchian, H. P.; Ortiz, J. V.; Izmaylov, A. F.; Sonnenberg, J. L.; Williams-Young, D.; Ding, F.; Lipparini, F.; Egidi, F.; Goings, J.; Peng, B.; Petrone, A.; Henderson, T.; Ranasinghe, D.; Zakrzewski, V. G.; Gao, J.; Rega, N.; Zheng, G.; Liang, W.; Hada, M.; Ehara, M.; Toyota, K.; Fukuda, R.; Hasegawa, J.; Ishida, M.; Nakajima, T.; Honda, Y.; Kitao, O.; Nakai, H.; Vreven, T.; Throssell, K.; Montgomery, J. A., Jr.; Peralta, J. E.; Ogliaro, F.; Bearpark, M. J.; Heyd, J. J.; Brothers, E. N.; Kudin, K. N.; Staroverov, V. N.; Keith, T. A.; Kobayashi, R.; Normand, J.; Raghavachari, K.; Rendell, A. P.; Burant, J. C.; Iyengar, S. S.; Tomasi, J.; Cossi, M.; Millam, J. M.; Klene, M.; Adamo, C.; Cammi, R.; Ochterski, J. W.; Martin, R. L.; Morokuma, K.; Farkas, O.; Foresman, J. B.; Fox, D. J. Gaussian 16, Revision C.01; Gaussian, Inc.: Wallingford, CT, 2019.
[11] (a) Grimme, S.; Antony, J.; Ehrlich, S.; Krieg, H. A Consistent and Accurate Ab Initio Parametrization of Density Functional Dispersion Correction (DFT-D) for the 94 Elements H-Pu. *J. Chem. Phys.* **2010**, *132*, 154104. (b) Grimme, S.; Ehrlich, S.; Goerigk, L. Effect of the Damping Function in Dispersion Corrected Density Functional Theory. *J. Comput. Chem.* **2011**, *32*, 1456. (c) Witte, J.; Mardirossian, N.; Neaton, J. B.; Head-Gordon, M. Assessing DFT-D3 Damping Functions Across Widely Used Density Functionals: Can We Do Better? *J. Chem. Theory Comput.* **2017**, *13*, 2043.
[12] (a) Hay, P. J.; Wadt, W. R. Ab Initio Effective Core Potentials for Molecular Calculations. Potentials for K to Au Including the Outermost Core Orbitals. *J. Chem. Phys.* **1985**, *82*, 299. (b) Ehlers, A. W.; Böhme, M.; Dapprich, S.; Gobbi, A.; Höllwarth, A.; Jonas, V.; Köhler, K. F.; Stegmann, R.; Veldkamp, A.; Frenking, G. A Set of f-Polarization Functions for Pseudo-Potential Basis Sets of the Transition Metals Sc-Cu, Y-Ag and La-Au. *Chem. Phys. Lett.* **1993**, *208*, 111. (c) Roy, L. E.; Hay, P. J.; Martin, R. L. Revised Basis Sets for the LANL Effective Core Potentials. *J. Chem. Theory Comput.* **2008**, *4*, 1029.
[13] (a) Ditchfield, R.; Hehre, W. J.; Pople, J. A. Self-Consistent Molecular-Orbital Methods. IX. An Extended Gaussian-Type Basis for Molecular-Orbital Studies of Organic Molecules. *J. Chem. Phys.* **1971**, *54*, 724. (b) Hehre, W. J.; Ditchfield, R.; Pople, J. A. Self-Consistent Molecular Orbital Methods. XII. Further Extensions of Gaussian-Type Basis Sets for Use in Molecular Orbital Studies of Organic Molecules. *J. Chem. Phys.* **1972**, *56*, 2257. (c) Hariharan, P. C.; Pople, J. A. The Influence of Polarization Functions on Molecular Orbital Hydrogenation Energies. *Theoret. Chim. Acta.* **1973**, *28*, 213.
[14] (a) Zhao, Y.; Truhlar, D. G. The M06 Suite of Density Functionals for Main Group Thermochemistry, Thermochemical Kinetics, Noncovalent Interactions, Excited States, and Transition Elements: Two New Functionals and Systematic Testing of Four M06-class Functionals and 12 other Functionals. *Theor. Chem. Acc.* **2008**, *120*, 215. (b) Zhao, Y.; Truhlar, D. G. Density Functionals with Broad Applicability in Chemistry. *Acc. Chem. Res*. **2008**, *41*, 157.
[15] Dolg, M.; Wedig, U.; Stoll, H.; Preuss, H. Energy-Adjusted Ab Initio Pseudopotentials for the First Row Transition Elements. *J. Chem. Phys.* **1987**, *86*, 866.
[16] Marenich, A. V.; Cramer, C. J.; Truhlar, D. G. Universal Solvation Model Based on Solute Electron Density and on a Continuum Model of the Solvent Defined by the Bulk Dielectric Constant and Atomic Surface Tensions. J. *Phys. Chem. B* **2009**, *113*, 6378.
[17] Legault, C. Y. CYLview, 1.0b; Université de Sherbrooke: Canada, 2009. http://www.cylview.org.
[18] Neese, F.; Wennnmohs, F.; Becker, U.; Riplinger, C.; The ORCA quantum chemistry program package. *J. Chem. Phys.* **2020**, *152*, 224108
